# Explosive Magnetotail Activity

**DOI:** 10.1007/s11214-019-0599-5

**Published:** 2019-05-16

**Authors:** Mikhail Sitnov, Joachim Birn, Banafsheh Ferdousi, Evgeny Gordeev, Yuri Khotyaintsev, Viacheslav Merkin, Tetsuo Motoba, Antonius Otto, Evgeny Panov, Philip Pritchett, Fulvia Pucci, Joachim Raeder, Andrei Runov, Victor Sergeev, Marco Velli, Xuzhi Zhou

**Affiliations:** 1 The Johns Hopkins University Applied Physics Laboratory, Laurel, MD USA; 2 Space Science Institute, Boulder, CO USA; 3 University of California Los Angeles, Los Angeles, CA 90095 USA; 4 Earth’s Physics Department, Saint Petersburg State University, St. Petersburg, Russia; 5 Swedish Institute of Space Physics, Uppsala, Sweden; 6 University of Alaska Fairbanks, Fairbanks, AK USA; 7 Space Research Institute, Austrian Academy of Sciences, Graz, Austria; 8 Department of Physics and Astronomy, University of California, Los Angeles, CA USA; 9 National Institute for Fusion Science, National Institutes of Natural Sciences, Toki, 509-5292 Japan; 10 Princeton Plasma Physics Laboratory, Princeton University, Princeton, NJ USA; 11 Institute for the Study of Earth, Oceans and Space, University of New Hampshire, Durham, NH USA; 12 Institute of Geophysics and Planetary Physics, University of California, Los Angeles, CA USA; 13 School of Earth and Space Sciences, Peking University, Beijing, 100871 China

**Keywords:** Magnetotail, Magnetic reconnection, Current sheet thinning, $B_{z}$ hump, Tearing instability, Ballooning/interchange instability, Flapping motions, Auroral beads/rays, Bursty bulk flows, Dipolarization fronts, Flux tube oscillations, Plasma micro-instabilities, Particle acceleration, Supra-arcade downflows, Laboratory reconnection experiments

## Abstract

Modes and manifestations of the explosive activity in the Earth’s magnetotail, as well as its onset mechanisms and key pre-onset conditions are reviewed. Two mechanisms for the generation of the pre-onset current sheet are discussed, namely magnetic flux addition to the tail lobes, or other high-latitude perturbations, and magnetic flux evacuation from the near-Earth tail associated with dayside reconnection. Reconnection onset may require stretching and thinning of the sheet down to electron scales. It may also start in thicker sheets in regions with a tailward gradient of the equatorial magnetic field $B_{z}$; in this case it begins as an ideal-MHD instability followed by the generation of bursty bulk flows and dipolarization fronts. Indeed, remote sensing and global MHD modeling show the formation of tail regions with increased $B_{z}$, prone to magnetic reconnection, ballooning/interchange and flapping instabilities. While interchange instability may also develop in such thicker sheets, it may grow more slowly compared to tearing and cause secondary reconnection locally in the dawn-dusk direction. Post-onset transients include bursty flows and dipolarization fronts, micro-instabilities of lower-hybrid-drift and whistler waves, as well as damped global flux tube oscillations in the near-Earth region. They convert the stretched tail magnetic field energy into bulk plasma acceleration and collisionless heating, excitation of a broad spectrum of plasma waves, and collisional dissipation in the ionosphere. Collisionless heating involves ion reflection from fronts, Fermi, betatron as well as other, non-adiabatic, mechanisms. Ionospheric manifestations of some of these magnetotail phenomena are discussed. Explosive plasma phenomena observed in the laboratory, the solar corona and solar wind are also discussed.

## Introduction

The Earth’s magnetosphere provides a global magnetic shield protecting life on our planet from the hazardous flow of solar wind plasma. This shield is not perfect: solar wind particles and interplanetary magnetic field flux may penetrate inside the magnetosphere and accumulate there, causing magnetic storms and substorms (Kamide et al. 1998). In contrast to storms, which are directly associated with large-scale solar wind disturbances, substorms often start suddenly, expanding within minutes after an hour-long preparatory or “growth” phase (McPherron 1970). It is known (Sergeev et al. 2012a; Angelopoulos et al. 2013) that the energy for such substorm explosions is accumulated in the Earth’s magnetotail, the night-side region where magnetic field lines of the Earth’s dipole field are stretched in the anti-sunward direction due to interaction with the solar wind flow past the magnetosphere. During the substorm expansion phase the highly stretched tail magnetic field becomes rapidly more dipolar. The mechanism behind this explosive dipolarization remains one of the major mysteries of magnetospheric physics.

Explosive energy release occurs at many different scales, and therefore observation methods, theories and models need to account for that. In particular, rapid dipolarizations are not limited to substorms and include pseudobreakups and dipolarization fronts (DFs) (e.g., Nakamura et al. 2002b) within bursty bulk flows (BBFs) (e.g., Ohtani et al. 2004; Angelopoulos et al. 2013) that occur on smaller time scales. The fast flows brake on approach to the near-Earth region (Shiokawa et al. 1997) and the dipolarized flux tubes may exhibit oscillations around their equilibrium position (Chen and Wolf 1999; Kepko and Kivelson 1999), damped due to the dissipation in the ionosphere.

The transition from slow to explosive evolution suggests that a plasma instability is at play. However, understanding the mechanisms of the explosive magnetotail activity ultimately requires an integrated investigation of the pre-onset conditions for the explosive instability, its onset mechanisms, modes of activity and their manifestations in the magnetosphere and ionosphere. Such an all-encompassing view of the explosive magnetic activity has not yet been reached by the scientific community. However, major strides have been made in recent years in understanding of various pieces of this puzzle and, in some cases, their interactions. The goal of this paper is to synthesize the knowledge on this major research topic in magnetospheric physics as it stands today.

In Sect. 2 we describe observations and models of the magnetotail evolution prior to its explosive reconfigurations and the resulting features that may be critical for the subsequent plasma instabilities. The evolution includes thinning of the tail current sheet (CS) down to the kinetic scale, comparable to the ion inertial length $d_{i}$, to form thin current sheets or TCSs (e.g., Sergeev et al. 2011). It also includes tailward stretching of the magnetic field lines (e.g., Petrukovich et al. 2007) and more complex redistributions of magnetic flux, such as the formation of a local minimum in the equatorial magnetic field and accumulation of magnetic flux further in the tail (Sergeev et al. 2018). Models of the slow evolution before the onset of activity include open magnetic flux accumulation (OMFA) due to the addition of flux reconnected at the magnetopause to the tail lobes (Birn and Schindler 2002) and earthward magnetospheric convection, as well as closed magnetic flux depletion (CMFD) due to the evacuation of the flux from the near-Earth tail by convection toward dayside after the start of reconnection at the subsolar magnetopause (e.g., Otto et al. 2015). Models of TCSs include conventional local (Grad-Shafranov-type) equilibria, with the current density expressed as a function of the vector potential at the same point (Schindler and Birn 2002), and nonlocal models, where the current density depends on either the local magnetic field or on the vector potential integrated over ion orbits (e.g., Sitnov et al. 2003).

In Sect. 3 we describe key mechanisms responsible for the transition from the slow evolution of the tail to its rapid (but not necessarily global) reconfiguration. Magnetotail dipolarizations are accompanied by fast earthward plasma flows (McPherron et al. 2011). These bursty flows were interpreted as reconnection ejecta coming from new X-lines (Russell and McPherron 1973; Baker et al. 1996). For a long time kinetic modeling of such tail reconnection regimes (Pritchett and Coroniti 1995; Hesse and Schindler 2001) involved squeezing the tail current sheet (using external driving) down to electron gyroradius scales resulting in demagnetization of electrons. We will refer to these reconnection regimes as Electron Demagnetization-Mediated Reconnection or EDMR thereafter. More recent studies revealed that magnetotail reconnection instabilities may also start directly from generation of fast flows followed by the formation of a new X-line because of the “magnetic flux starvation” effect (Sitnov et al. 2013; Bessho and Bhattacharjee 2014; Pritchett 2015). In contrast to EDMR, reconnection in this regime arises as a result of the development of an instability similar to the long-sought ion tearing instability (Schindler 1974), which only requires demagnetization of ions. Therefore we refer to this reconnection regime Ion Demagnetization-Mediated Reconnection or IDMR. In contrast to EDMR, IDMR may start as an ideal-MHD instability and hence develop spontaneously already on MHD scales (Merkin et al. 2015; Birn et al. 2018).

The concept of spontaneous magnetic reconnection (Galeev 1984; Treumann and Baumjohann 2015) has always been central to the discussions of the magnetotail explosion mechanisms (Pellat et al. 1991; Hesse and Schindler 2001; Sitnov et al. 2002). A unique opportunity to reveal the inner workings of magnetic reconnection, including the mechanisms of collisionless (Landau) dissipation, appeared after the launch in 2015 of the Magnetospheric MultiScale (MMS) mission (Burch et al. 2016). While MMS observations are in progress and are still awaiting a dedicated and comprehensive theoretical review and interpretation, we ventured to outline below some of their key findings relevant to the explosive magnetotail activity.

In addition to magnetic reconnection, magnetotail activity also includes ballooning/interchange (B/I) and flapping instabilities. B/I motions bring flux tubes with reduced content of plasma from the depths of the tail toward the planet, similar to air bubbles in water lifted to the surface by Archimedes (buoyancy) force (Pontius and Wolf 1990). The development of the B/I instability in regions with tailward gradients of the equatorial magnetic field $B_{z}$ may preclude the formation of such regions and hence the development of reconnection in the IDMR regime. At the same time, one can expect the formation of new X-lines in the trails of the B/I fingers (Pritchett and Coroniti 2013). Flapping motions represent global oscillations of the tail current sheet as a whole like a flapping flag. Flapping waves propagate from the midnight meridian toward the dawn and dusk flanks, i.e., normal to the solar wind propagation direction (Sergeev et al. 2004, 2006). Flapping instabilities can be reproduced in some magnetohydrodynamic (MHD) (Korovinskiy et al. 2013) and kinetic particle-in-cell (PIC) (Pritchett and Coroniti 2001; Sitnov et al. 2014) simulations.

At the end of Sect. 3, we describe ionospheric signatures of the magnetotail activity before, at and after its onset. They include auroral streamers, beads, undulating arcs, equatorward and poleward boundary expansions, as well as their substructures (e.g. Motoba et al. 2012; Nishimura et al. 2016).

In Sect. 4 we describe observations of magnetotail dynamics, simulations of magnetotail transients, micro-instabilities, some features of particle distributions during explosive magnetotail activity and the damped oscillations of the dipolarized flux tubes in the near-Earth region. Mesoscale earthward transients largely known as BBFs have sharp (on the order of the ion inertial scale $d_{i}$) DF boundaries at their leading edges (Runov et al. 2009). These boundaries mark a rapid transition between downstream and upstream plasma properties, such as ion and electron temperatures. The corresponding sharp plasma and field gradients may become sources of micro-instabilities, such as the lower-hybrid drift and mirror instabilities (Khotyaintsev et al. 2011) that transfer the energy to small scales via collisionless Landau dissipation. Details of particle interaction with DFs are investigated particularly successfully using test-particle tracing in MHD and ad hoc DF electromagnetic field models (Birn et al. 2012, and refs. therein). Braking of the dipolarizing flux tubes near Earth has been suggested to result in the build-up of the substorm current wedge (Shiokawa et al. 1997; Birn et al. 1999; Liu et al. 2014a; Kepko et al. 2015). It may also cause their damped oscillations which represent one of the main sinks of energy in the tail due to dissipation in collisional plasmas of the ionosphere (Panov et al. 2016).

Similar explosive activity is observed in magnetotails of other planets and their moons. However, since comparative properties of magnetotails in the solar system have recently been reviewed (e.g., Keiling et al. 2015, and refs. therein) we forgo this interesting topic and include instead in Sect. 5 the discussion of similar explosive activity in the solar corona and some laboratory experiments.

Each (sub)section ends with a quick summary of the takeaways (Key points) as well as with a brief list of Open questions which by no means is exclusive, but reflects the opinions of the authors of this review. The main features of the explosive magnetotail activity discussed below are finally summarized in Sect. 6.

## Observations and Modeling of Pre-onset Features

A crucial problem in the evolution towards onset of magnetotail activity is not only the characterization of configurations that are potentially unstable but also the identification of the conditions that lead from a quasi-stable, gradual evolution toward a fast, unstable or explosive, evolution. It has been known for a long time that, in general, a southward component of the interplanetary magnetic field favors entry of solar wind particles, magnetic flux and energy into the magnetosphere, via frontside reconnection, which leads to transport and accumulation of magnetic flux and energy in the tail. However, the exact conditions that cause a change in stability properties and the identification of the onset instability are insufficiently analyzed. It is fairly well documented, both observationally and through theory and simulations, that the formation of a localized TCS, embedded in a wider tail plasma sheet, plays a crucial role in the initiation of activity. More recently the importance of the magnetic flux redistribution in the closed field line region has been recognized. Below in this section we discuss observations and theoretical models of TCS formation and flux redistribution, as well as other aspects of the pre-onset tail current sheet modification, including TCS observations and models.

### Observations

The tail current sheet is a high-beta region near magnetotail equatorial plane, where the main (tail-aligned) magnetic field component $B_{x}$ changes its sign. Typical CS thickness is a few $\mbox{R}_{E}$, and the lobe magnetic field strength decreases downtail from $\sim40\mbox{--}60~\mbox{nT}$ at $\sim12\mbox{--}15\,\mbox{R}_{E}$ to $\sim10~\mbox{nT}$ at $\sim60\,\mbox{R}_{E}$ (Slavin et al. 1985). Transition from dipole-like to tail-like configuration occurs between $9\mbox{ and }12\,\mbox{R}_{E}$ (Ohtani and Motoba 2017). In the equatorial plane of the tail-like region the magnetic field component $B_{z}$ normal to the CS decreases on average from ∼15 nT at $\sim12\,\mbox{R}_{E}$ to $\sim 0.5~\mbox{nT}$ at $\sim 60\,\mbox{R}_{E}$ (Behannon 1970; Ohtani and Motoba 2017). The typical current density in the quiet-time CS is a few $\mbox{nA}/\mbox{m}^{2}$.

The magnetotail configuration is known to change considerably during 0.5–1.5 hour-long substorm growth phase (e.g., Baker et al. 1996) when the open magnetic flux provided by the dayside reconnection is loaded into the magnetotail. Major changes include a decrease in the $B_{z}$ component as well as current sheet thinning and restructuring, including the formation of a TCS embedded into a thicker plasma sheet. The details of this process have been studied using multi-probe missions CLUSTER and THEMIS (e.g., Nakamura et al. 2002a; Runov et al. 2003, 2005, 2006, 2012; Sergeev et al. 2003, 2011; Petrukovich et al. 2007, 2013; Saito et al. 2011; Artemyev et al. 2016b), and an example is given below.

**Fig. 1:**
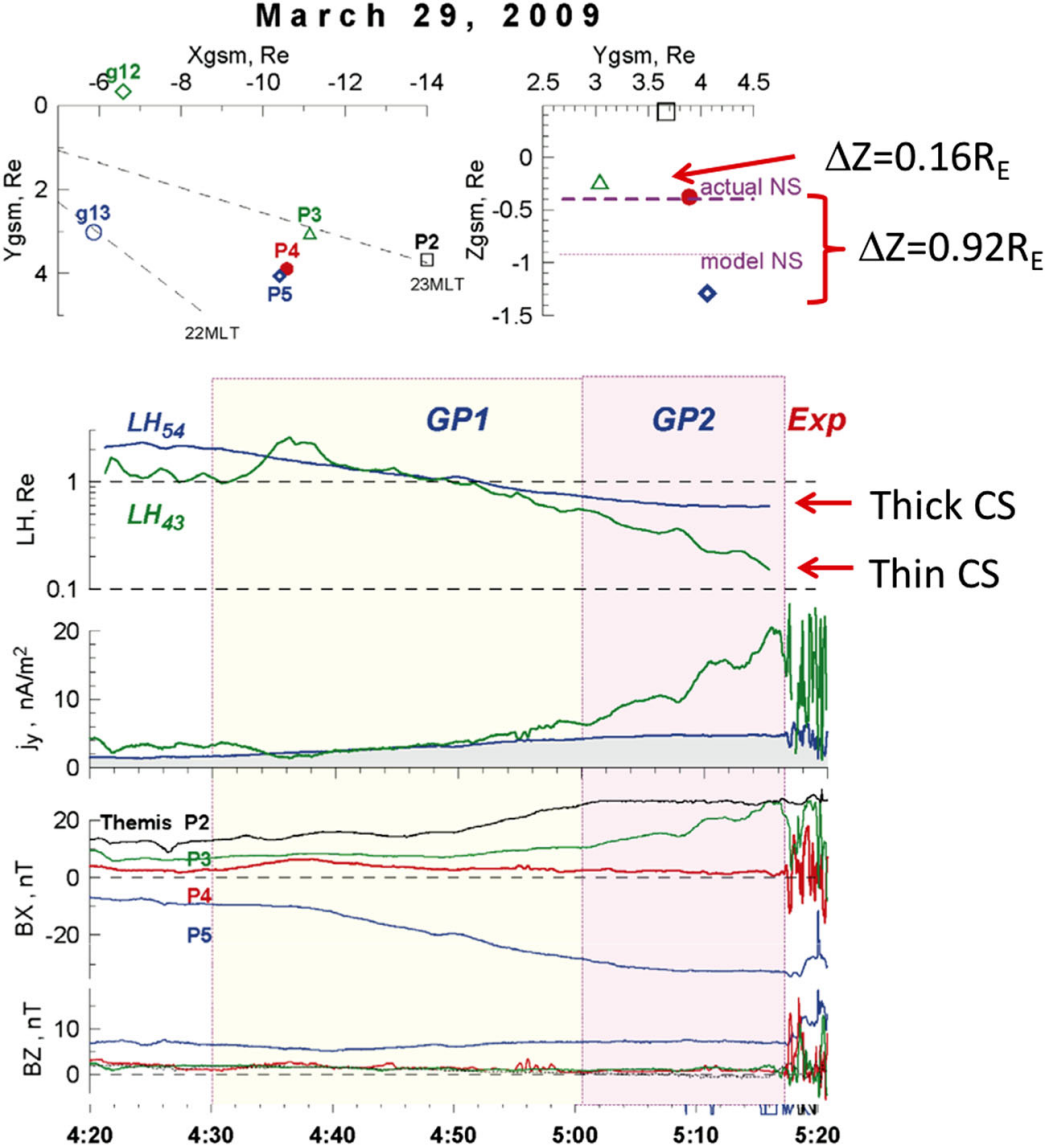
Formation of the embedded TCS in the late growth phase of the March 29, 2009 substorm. Top panels show GSM coordinates of spacecraft and bottom panels show THEMIS P2–P5 observations, including (from bottom to top) $B_{z}$ and $B_{x}$ GSM magnetic field components, estimates of cross-tail current $j_{y}$ using differences of $B_{x}$ components at pairs P3–P4 and P4–P5, and estimates of Harris current sheet thickness $L_{z}=L_{H}$ for the same pairs of spacecraft. Adapted from Sergeev et al. (2011)

Typical current sheet changes during the substorm growth phase are illustrated in Fig. 1. It shows observations of four THEMIS spacecraft in the fortuitous configuration, with P4 spacecraft probing the CS center, two spacecraft P2 and P5 monitoring the basic current sheet at $\sim 1\,\mbox{R}_{E}$ above and below the CS center plane, and with P3 spacecraft having small $Z$-separation from P4. Such configuration of probes allows one to check the formation of embedded TCS during last 10 minutes before the explosive onset (Sergeev et al. 2011). Figure 1 shows a gradual $B_{z}$ decrease in the CS center (reaching values as small as 1–2 nT at $R\sim 11\,\mbox{R}_{E}$), and overall increase of the tail current (the increasing distance between P2 and P5 $B_{x}$ curves) during the growth phase, excluding the last 15 minutes of the faster TCS growth marked by the bottom red arrow. The CS thinning process can be seen in Fig. 1 (the third and second panels from the bottom) as the increase of the current density $j_{y}$ and the magnetic field component $|B_{x}|$. The accompanying stretching of the tail field lines, which is usually seen as a decrease of $B_{z}$ magnetic field components, is not very pronounced in this particular example, and it will be discussed in more detail further in this section.

Distributions of fields and plasma parameters across the CS can be studied using various single- and multi-probe methods. First, one can use rapid flapping north-south motions of the CS to scan its structure. These motions can be detected as sign-alternating variations of the $z$-component of the ion bulk flow velocity anti-correlating with variations in $B_{x}$ magnetic field component. Their combined analyses applied to AMPTE/IRM observations (Sergeev et al. 1998) showed that at distances $12\mbox{ to }18\,\mbox{R}_{E}$ current sheet thickness varies in the range $0.2\mbox{--}1\,\mbox{R}_{E}$, and the current density may reach $20~\mbox{nA}/\mbox{m}^{2}$ during substorm episodes. In another method of the CS thickness evaluation, the CS magnetic field is approximated by the Harris model (Harris 1962) $B_{x}=B_{0}\tanh(z/L_{H})$, to estimate the CS’s halfthickness $L_{z}=L_{H}$ and current density $j_{y}\propto\partial B_{x}/\partial z$ as in Fig. 1. Two-point measurements with ISEE 1/2 probes revealed that the current sheet thickness at $R=11\mbox{ to }20\,\mbox{R}_{E}$ decreased down to $L_{z}\sim 0.1\,\mbox{R}_{E}$ and the current density may increase up to $j_{y}\approx 50~\mbox{nA}/\mbox{m}^{2}$ during dynamic events and substorm growth phases (McComas et al. 1986; Sergeev et al. 1993b; Sanny et al. 1994).

Reconstructions of the tail current sheet structure have been strongly improved by the four-probe Cluster mission. During flapping events observed at $R\approx 19\,\mbox{R}_{E}$ Runov et al. (2005) found that the current sheet thickness $L_{z}$ varies between $1\mbox{ and }20 d_{i}$, where $d_{i}=c/\omega_{pi}$ is the ion inertial length and $\omega_{pi}$ is the ion plasma frequency calculated using the plasma density at $B_{x}\approx0$. Its value in the plasma sheet is about 400 km (e.g., Runov et al. 2006). The reconstructed profiles of the current $\mathbf{j}(z)$ and ion density $n_{p}(z)$ are different: the current sheet is typically embedded into a much thicker plasma sheet (Runov et al. 2006). These results should be taken with some caution because flapping motion intervals, during which the CS structure was probed, might represent a special state of the CS (e.g., active CS).

A complementary approach to the Cluster data analysis was developed by Asano et al. (2005) who excluded flapping events, but selected instead configurations with one probe being close to the CS center ($B_{x} \sim 0$) and another one being in between the center and a lobe (two remaining probes were used to estimate the tilt of the current sheet). Despite the different approach, the results of Asano et al. (2005) turned out to be consistent with Runov et al. (2006). They confirmed that CSs are typically embedded into the thicker plasma sheets (thus forming complex structures different from the classical Harris solution), and that the CS scales are often comparable to the ion inertial length.

Petrukovich et al. (2011) analyzed embedded TCSs at the radial distances $r\sim15\mbox{--}19\,\mbox{R}_{E}$ using the flapping motion approach. They found that the typical TCS half-thickness is about 1–3 ion gyroradius (in the field $B_{0}$ defined at the outer boundary of the TCS, $z_{0}$), and estimated the magnetic flux per unit length in $Y$ ($F_{0}=z_{0} B _{0}$) to be 0.006–0.03 Wb/m, more than an order of magnitude smaller compared to the total closed magnetic flux. At $r\sim11\,\mbox{R}_{E}$ distance, the fast growth of embedded TCS during last several minutes before the substorm onset was found in the absence of an accompanying total pressure growth (Saito et al. 2011; Sergeev et al. 2011). This implies that the lobe pressure increase is not the only reason for the CS thinning. Recent observations by THEMIS at $10< R<30\,\mbox{R}_{E}$ (Artemyev et al. 2014), Geotail at $20< r<50\,\mbox{R}_{E}$ and at $80< R<200\,\mbox{R}_{E}$ (Vasko et al. 2015), and ARTEMIS $R\sim 60\,\mbox{R}_{E}$ (Xu et al. 2018) confirmed that at those distances the CS thickness might be as small as $\sim 1000~\mbox{km}$, comparable to the ion inertial length.

**Fig. 2:**
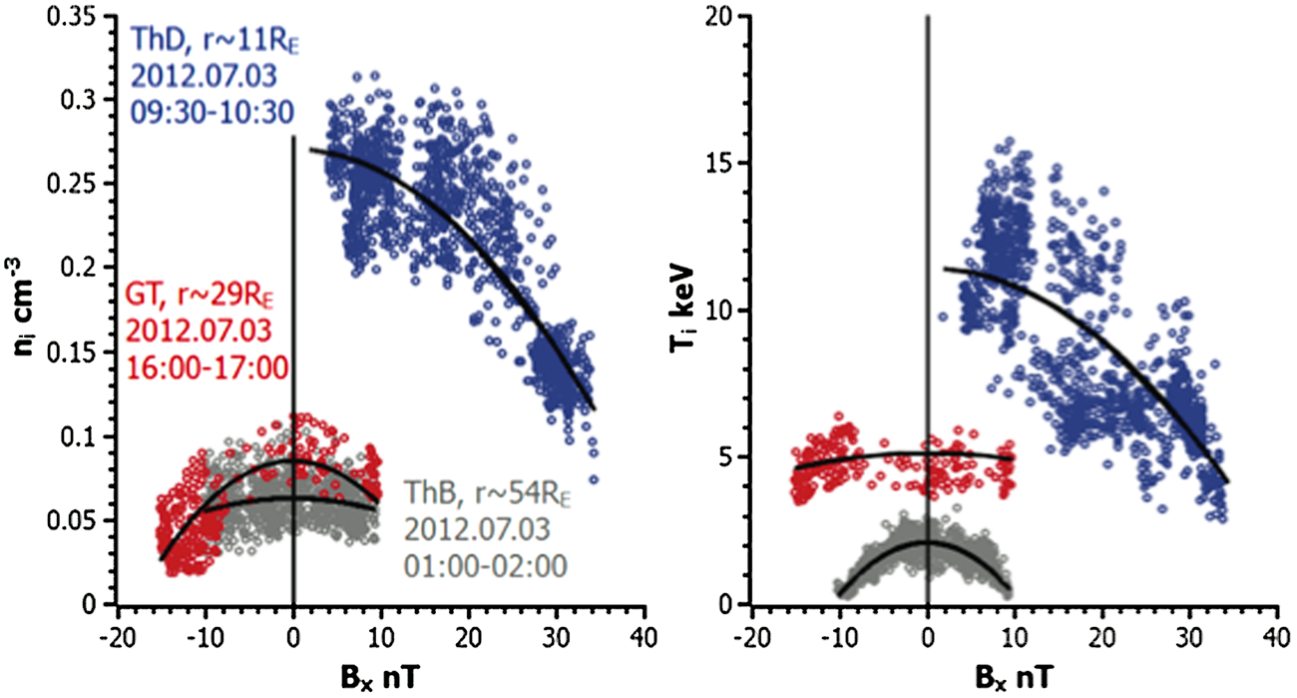
(Left) Plasma density and (right) temperature profiles across the magnetotail CS. Black lines show the corresponding analytical approximations. Adapted from Artemyev et al. (2017, Fig. 3)

The dominant current carriers in TCSs at $r\sim11\,\mbox{R}_{E}$ may be both ions and electrons (Artemyev et al. 2016b). Most recently, the embedded structure of the TCS has been demonstrated (Artemyev et al. 2017) in the form of the substantial temperature gradients across the sheet (Fig. 2).

Correlation of the CS thinning and magnetic field line stretching processes has been studied by Petrukovich et al. (2007, 2013) and Artemyev et al. (2016b). In particular, Petrukovich et al. (2007) investigated the dependence between the normal to the CS plane magnetic field component $B_{n}$ in the equatorial plane and the current density $j_{y}$ measured by Cluster at $R\sim 19\,\mbox{R}_{E}$ during the substorm growth phase. They found that thinning (i.e., the $j_{y}$ increase) is accompanied by a magnetic field stretching (the $B_{n}$ decrease) such that $j_{y}\varpropto{1/B_{n}}$. The $B_{n}$ amplitude immediately prior to a fast flow (taken as onset signature) varied between 2 and 7 nT. Petrukovich et al. (2013) showed a correlation between the decrease of $B_{z}$ and the increase of the $(\partial B_{x}/\partial z)/( \partial B_{z}/\partial x)$ parameter in the growth phase. They found that for 10 near-Earth events with stable positive $\partial B_{z}/ \partial x$ (earthward $B_{z}$ gradient) quasi-1D configurations with $\partial B_{x}/\partial z \gg\partial B_{z}/\partial x$ developed quickly, when $B_{z}$ was below 4–6 nT.

**Fig. 3:**
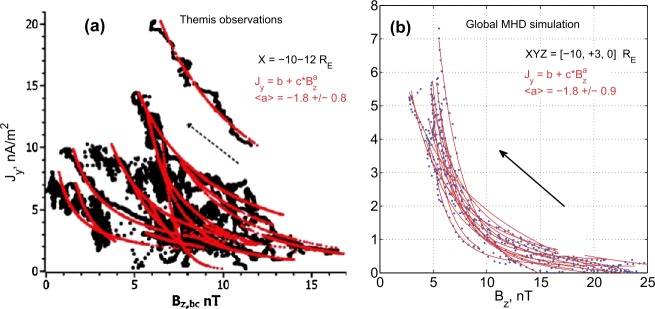
Hodograms showing the relationship between peak current density $J_{y}$ and $B_{z}$ during the substorm growth phase events near the neutral sheet at $\sim11\,\mbox{R}_{E}$ distances: (**a**) THEMIS multispacecraft observations from Artemyev et al. (2016b); (**b**) global MHD simulations from Gordeev et al. (2017b)

Artemyev et al. (2016b) used THEMIS observations to study properties of CS thinning at closer distances, in the tail-dipole transition region at $R\sim 10\mbox{--}12\,\mbox{R}_{E}$. A notable feature of evolution found was a rapid increase of tail-aligned plasma pressure gradient $\partial p/\partial x$, whose scale $L_{x}$ decreases down to a few thousand kilometers during CS thinning. The current density $j_{y}$ was estimated from three-point magnetic field measurements. For 17 selected events a gradual $B_{z}$ decrease and $j_{y}$ increase were observed, as is shown in Fig. 3a. On average, the current density varied as $j_{y}\varpropto{B_{z}}^{-7/4}$. The $B_{z}$ value at the onset varied between a fraction of nT and $\approx7~\mbox{nT}$. Similar $j_{y}(B_{z})$ scaling and pre-onset $B_{z}$ values have recently been obtained in global MHD simulations (Gordeev et al. 2017b, see Fig. 3b). The set of idealized MHD runs did not necessarily correspond to the same conditions that underlied the statistical observations (Fig. 3a), and thus the scale of the MHD variations (Fig. 3b) is different from the observations (note the difference in axes limits in the two panels). Nevertheless, Fig. 3 demonstrates that global MHD simulations reproduce important aspects of the magnetotail stretching and thinning processes.

It is not yet clear from observations whether the described increase in the current density is accompanied by an increase in the plasma density or temperature. THEMIS observations of CS thinning at $r\sim 10\mbox{--}12\,\mbox{R}_{E}$ suggested an increase in density with a decrease in ion and electron temperatures (Artemyev et al. 2016b). These results, however, were not yet confirmed for other distances and were not reported in simulations.

**Fig. 4:**
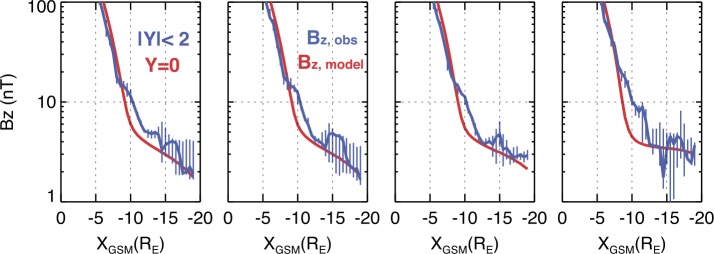
Equatorial radial profiles of observed $B_{z}$ (blue curves, in regions with plasma beta $>2$) and the model $B_{z}$ (red curves) at midnight for four different PCPAE loading parameter (from left to right—for loading rates $\mbox{PCPAE} = 100, 2000, 4000, \mbox{and } 8000~\mbox{kV}\,\mbox{min}$) with $P_{sw} = 2~\mbox{nPa}$ (adapted from Yue et al. (2015))

For the tail stability problem (Sect. 3), the $B_{z}$-component value and its radial distribution in the tail neutral plane are among the most interesting parameters. However, the corresponding empirical picture is still very limited. A set of $B_{z}$ profiles for different values of the special loading parameter PCPAE, a linear combination of the time integrated cross polar cap potential drop (CPCP) and the auroral index $AE$, obtained by Yue et al. (2015) is shown in Fig. 4. At $18\mbox{--}20\,\mbox{R}_{E}$ distance the equatorial $B_{z}$ values are as small as $2\mbox{--}3~\mbox{nT}$ for all loading rates, consistent with $B_{z} \sim1\mbox{--}2~\mbox{nT}$, $j_{0}\sim4\mbox{--}8~\mbox{nA}/\mbox{m}^{2}$, and CS thickness (Harris estimate) $>3000~\text{km}$ obtained by Petrukovich et al. (2007). At $r\sim 11\,\mbox{R}_{E}$ both observed and modeled $B_{z}(j_{y})$ scaling curves in Fig. 3 show $B_{z}$ ranging between 2 nT and 5–7 nT, which is smaller than $\sim 10~\mbox{nT}$ value at $r\sim 10\,\mbox{R}_{E}$ in Fig. 4. While Fig. 4 does not reveal any $B_{z}$ humps, it clearly shows the consistent reduction of the earthward $B_{z}$ slope with the increase of the PCPAE whose largest values correspond to the late substorm growth phase

A Cluster survey of $B_{z}$-gradients during growth phase events (Petrukovich et al. 2013), displayed only 4 cases supporting a substantial $B_{z}$ hump at $r\gtrsim15\,\mbox{R}_{E}$, whereas no sign of such $B_{z}(r)$ variation in this region is seen with similar spacecraft configurations in a dozen other events. Values of $B_{z}$ peaks documented so far are not big, they typically do not exceed 6–8 nT in the data, as is seen from Fig. 7 by Petrukovich et al. (2013). Sparse spacecraft coverage and the difficulty of removing tilt-related dynamical contributions to $B_{z}$ provide a major obstacle to systematic investigation of a non-monotonic $B_{z}(r)$ variation from in situ observations.

**Fig. 5:**
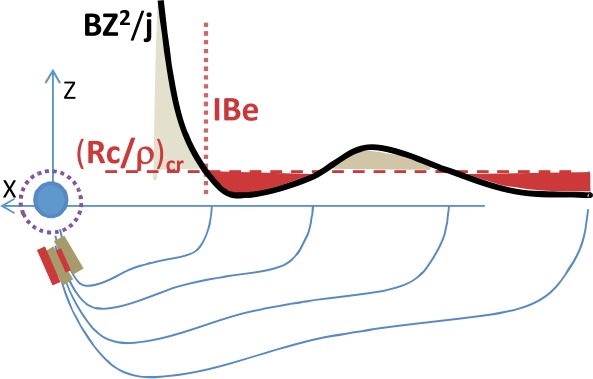
Schematic of equatorial $B_{z}^{2}/j$ expected in case of non-monotonic $B_{z}(r)$ variation around the loss-cone filling threshold for 30 keV electrons and expected pattern of isotropic (red) and anisotropic (grey) loss cone regions (Sergeev et al. 2018)

**Fig. 6:**
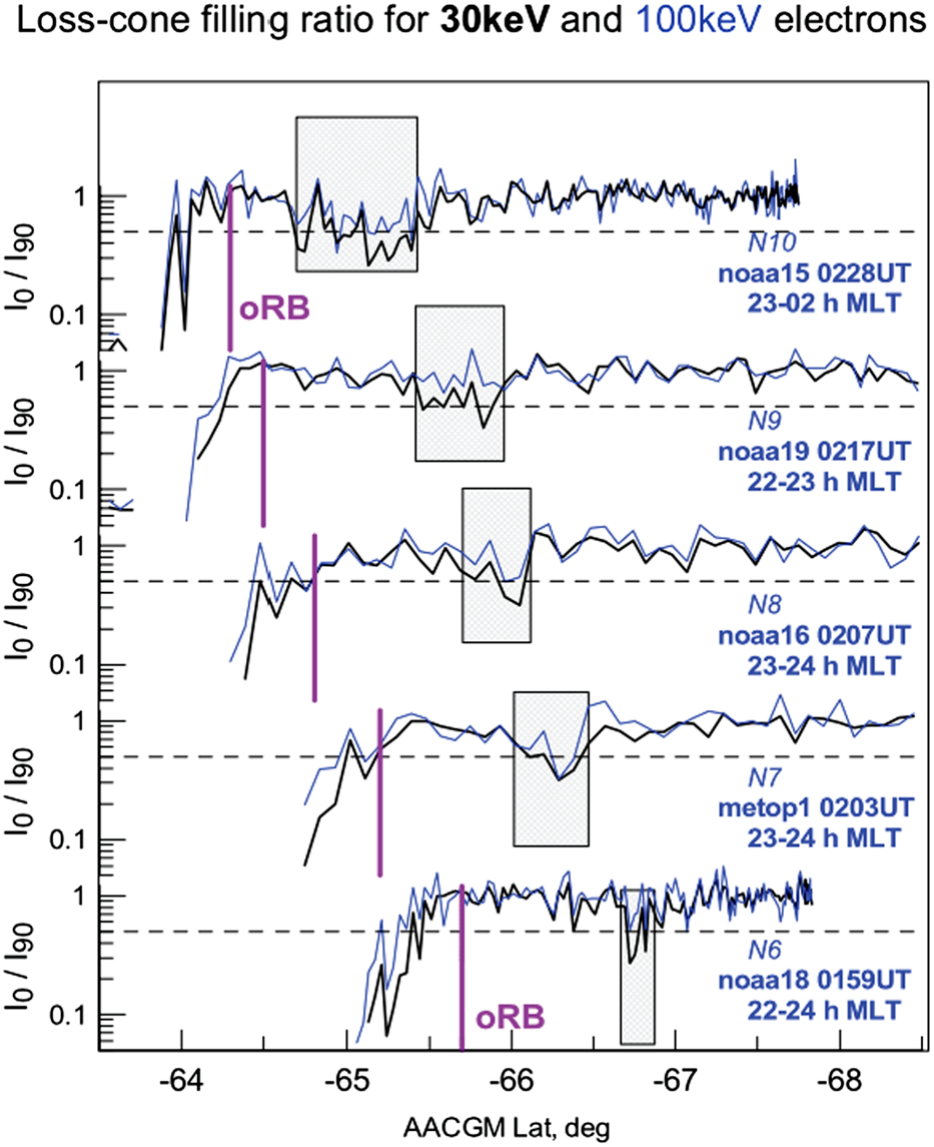
Loss cone filling rate (ratio of precipitated to trapped fluxes) during 5 consequent premidnight traversals of POES-type spacecraft. Anisotropic LC regions corresponding to $B_{z}$ peak in the equatorial plane are emphasized by shadowing (Sergeev et al. 2018)

Meanwhile, global distributions of the equatorial $B_{z}$ component can be investigated remotely, by observing the loss-cone (LC) filling rate at the low-altitude spacecraft which quickly traverse across the nightside auroral oval. The LC scattering is controlled by the magnetic field curvature in the CS center plane (more specifically, by the $B_{z}^{2}/j$ ratio). Energetic ($E>30\text{ keV}$) electrons are suitable tracers of $B_{z}$ in the tail because their LC filling threshold occurs near $B_{z}\sim 5\mbox{ to }10~\mbox{nT}$ in typical magnetotail conditions at around $15\mbox{--}20\,\mbox{R}_{E}$, i.e., in the range expected for non-monotonic peak/valley $B_{z}$ variations (see the schematic in Fig. 5). Sergeev et al. (2018) presented successive crossings of the premidnight auroral oval by six low-orbiting Polar Operational Environmental Satellites (POES) spacecraft during the growth phase of an intense, isolated substorm. All of these crossings show a narrow ($0.2\mbox{--}0.5^{\circ}$ AACGLat width) region of anisotropic LC fluxes (the expected signature of a $B_{z}$ peak in the equatorial magnetotail) embedded inside of the wide isotropic (a signature of low $B_{z}$) loss-cone precipitation region, formed in the middle and distant tail current sheet, respectively. This structure was observed $\sim1^{\circ}$ AACGLat poleward of the outer boundary of the radiation belt (marked as oRB in Fig. 6) for more than 30 minutes, during a slow expansion of the auroral oval.

Robust observations of such effects are limited to rare intense electron-rich solar particle events which provide sufficiently high count rate of $30\text{ keV}$ tracer electron fluxes from the entire plasma sheet. In such favorable conditions the structures in question are not always observed: from 8 available growth phase events Sergeev et al. (2018) identified robust signatures of non-monotonous $B_{z}(r)$ only in 2 events (the lack of them in remaining events may be also partly due to sparse spacecraft coverage and due to threshold nature of remote detection method). In any event, the aforementioned remote sensing results provide important evidence that a few hours MLT-wide $B_{z}$-ridge-like structures are formed in the tail at $15\mbox{--}20\,\mbox{R}_{E}$, and that they may persist there for more than 0.5 hour without being destroyed.

Most recently, these remote-sensing findings have been confirmed by the empirical analysis of the substorm magnetic field based on a new data-mining approach (Stephens et al. 2019). The analysis revealed that the formation of deep $B_{z}$ minima at $r \sim11\,\mbox{R}_{E}$ and humps at $r \sim16\,\mbox{R}_{E}$ is a prominent feature of the late substorm growth phase. In summary, in agreement with multi-spacecraft event studies, remote sensing results confirm that persistent $B_{z}$-ridge-type structures are real objects during the substorm growth phase in some events, but also caution us that they may not be present in all events (or they may be shallow structures).

Thus, multi-spacecraft studies over the last-decade strongly expanded and quantified our knowledge of the gradual, but ultimately very strong, reconfiguration of the magnetotail prior to its explosion. The main signatures of the reconfiguration are strong CS thinning and current density increase with the formation of multiscale embedded TCS structure, strong stretching of the tail because of the decrease of $B_{z}$ and increase of $|B_{x}|$ magnetic field components, especially in the transition region (roughly, $8\mbox{--}15\,\mbox{R}_{E}$). Also, there is growing observational evidence that another distinctive feature of the growth/pre-onset phase is the formation (probably at $15\mbox{--}20\,\mbox{R}_{E}$) of areas with locally accumulated magnetic flux (so-called “$B_{z}$-humps”), on the earthward side of which the gradient of the equatorial magnetic field $B_{z}$ is directed tailward. As is shown below in Sects. 3.2–3.4, the latter feature is a critically important condition for one of the regimes of spontaneous magnetic reconnection (IDMR) and it also appears prior to reconnection onset in the other regime (EDMR).

*Key points:*

Observed pre-onset features of the magnetotail include: (1) its stretching and thinning with the formation of the ion-scale TCS embedded in a much thicker plasma sheet, resulting into a complex multi-scale structure of plasma parameters across the tail and (2) redistribution of the magnetic flux along the tail sometimes resulting in the formation of regions with a tailward gradient of the equatorial magnetic field $B_{z}$.

*Open questions:*

What is the picture of systematic plasma parameter (density, temperature) variations in the tail at $10\mbox{--}20\,\mbox{R}_{E}$?

How generic are TCSs and $B_{z}$ humps in the pre-onset magnetotail and what are their characteristics and formation conditions and pre-conditions?

What is the radial and local time extension of the pre-onset TCSs?

What are other non-MHD and/or global features of the pre-onset magnetotail (e.g., temperature gradients, cold dense plasma sheet)?

### Modeling the Pre-onset Reconfiguration

The gradual magnetotail reconfiguration prior to its explosion is determined by an interplay of two major processes, the accumulation of magnetic flux in the tail lobes supplied by magnetopause reconnection and flux depletion at low latitudes in the near-Earth region on closed field lines, where the flux is tapped sunward through the flanks to feed the same dayside reconnection.

#### Open Magnetic Flux Accumulation Under High-Latitude Driving

The well-documented increase of magnetic flux in the lobes in the substorm growth phase is strongly suggestive of its importance in the generation of current sheet thinning or of an intensified current sheet embedded in the wider plasma sheet. However, a simple 1D scaling of the effects of an increase of the lobe field strength by some factor $f$ would suggest that the current density should increase by a factor $f^{2}$ while the current sheet thickness would decrease by $1/f$. Many PIC simulations have indicated that a half-thickness of less than an ion inertial length of, e.g., 500 km, is necessary to render a current sheet unstable (disregarding the stabilizing effects of a normal magnetic field component, to be discussed in Sect. 3.1). The reduction of the current sheet thickness from quiescent value of, e.g., 10,000 km to 500 km thus would require a lobe field increase by a factor of 20. This is completely incompatible with observations. However, the lobe boundary deformation through OMFA (open magnetic flux accumulation) will in general result in a two or three-dimensional evolution of the tail equilibrium.

An alternative to the 1D scenario was given by Birn and Schindler (2002), who investigated sequences of stretched 2D magnetotail equilibria under the conservation of mass and entropy content on closed field lines. The latter can be expressed, for an isotropic pressure, by $S=\int p^{1/ \gamma}dV$, where $dV=ds/B$ is the differential volume of a flux rope of unit magnetic flux and the integral is along a closed magnetic field line from one (ionospheric) foot point to the other. This quasi-static evolution is equivalent to ideal MHD under slow adiabatic changes. Assuming boundary deformations consistent with a stronger increase of magnetic field closer to Earth, they found that the adiabatic sequence might lead to critical states, at which neighboring equilibria ceased to exist. The evolution toward the critical state was characterized by the formation of a TCS, embedded in the wider plasma sheet and extending into bifurcated thin sheets toward earth. At the critical state the current density of the embedded current sheet would go to infinity. The theoretical results were confirmed by ideal MHD simulation and extended to 3D (Birn et al. 2004b).

The entropy function $S$ plays another important role. As Schindler and Birn (2004) have demonstrated, a monotonic increase of $S$ with distance along the tail renders the tail stable to ballooning/interchange modes. An adiabatic ideal evolution that conserves $S$ on each field line would not change that monotonicity as long as field lines remain simply connected to the ionospheric boundary. In contrast to the variation of $S$, the magnetic field strength $B_{z}$ does not remain monotonic and typically develops a local minimum, when the tail configuration evolves toward a critical point.

**Fig. 7:**
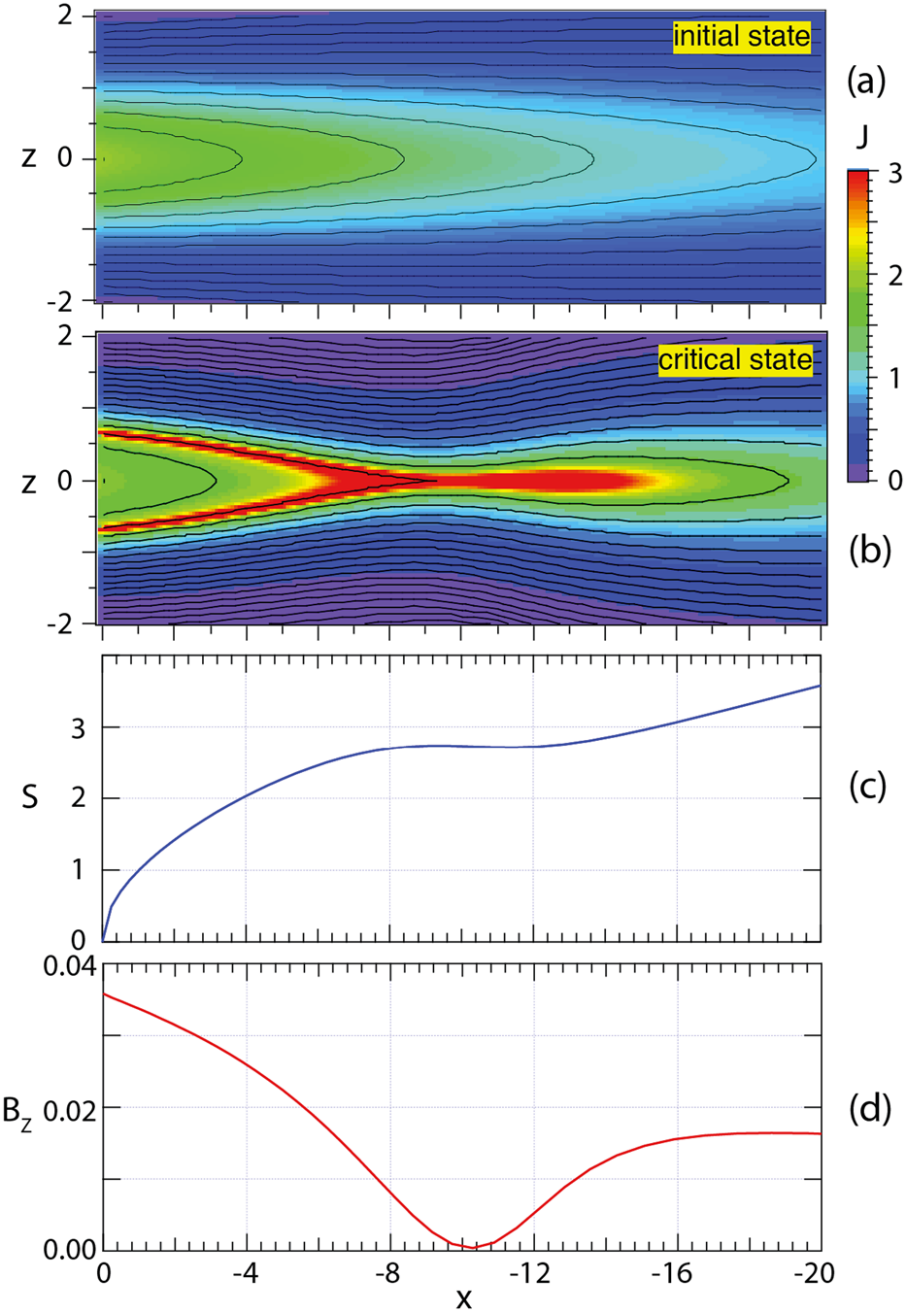
Properties of a 2D quasi-static tail equilibrium deformed adiabatically via boundary deformation, corresponding to the second example in Birn and Schindler (2002). The top two panels show magnetic field lines and the color-coded current density of the initial and the critical state (akin to Fig. 8a of Birn et al. 2009). The panels below show the entropy integral $S$ and the magnetic field component $B_{z}$ of the critical state at $z=0$ as functions of $x$

This is illustrated in Fig. 7, which shows properties at the critical state based on example 2 in Birn and Schindler (2002). Figure 7a shows magnetic field lines and the color-coded current density of the initial state, Fig. 7b that same of the deformed state at the critical threshold (equivalent to Fig. 8a in Birn et al. 2009). Figures 7c and 7d show the entropy function $S$, defined by Eq. (1), and the magnetic field component $B_{z}$ at $z = 0$ as functions of $x$.

The loss of equilibrium, however, does not necessarily lead to the onset of explosive activity. It is as well possible that one of the imposed conditions becomes violated in a more benign manner by, for instance, a change in topology or the development of 3D structure. Thus, it is probably more important that the evolution toward the critical state is characterized by the development of a thin current sheet, which eventually can undergo instability such as tearing. This is indeed confirmed by PIC simulations to be discussed in Sect. 3.2. It should be noted that the TCS formation from high-latitude boundary deformation is not restricted to the addition of magnetic flux. Other possible mechanisms include pressure pulses in the solar wind or sudden IMF changes (Birn and Schindler 2002). Yet another opportunity is the transformation of isotropic MHD-like CS into more complex TCS equilibria, as described, for example, in Schindler and Birn (2002), Birn et al. (2004c), Sitnov et al. (2000, 2003), Sitnov and Merkin (2016), Zelenyi et al. (2003) and discussed in more detail in Sects. 2.4 and 2.5. The complexity can be provided by plasma anisotropy or even agyrotropy.

*Key points:*

(1) The formation of singularly thin CSs under finite deformations of the magnetotail boundary (corresponding to high-latitude driving and OMFA) can be explained in the theory of quasi-static (isentropic) evolution of isotropic Grad-Shafranov-type equilibria. (2) The equilibrium theory of the boundary deformations is confirmed by MHD simulations. (3) The resulting singular TCSs may either explode due to a plasma instability or transform into more general classes of TCS described in Sects. 2.4 and 2.5.

*Open questions:*

How generic is the relationship between thin current sheet formation and the evolution toward loss of equilibrium? Are there paths to the loss of neighboring equilibrium that do not involve TCS formation?

#### Closed Magnetic Flux Depletion Under Low-Latitude Driving

In addition to lobe boundary deformations through OMFA (Sect. 2.2.1), a typical property associated with thin current sheet formation is closed magnetic flux depletion (CMFD) through low latitude driving and observed in the strong reduction of $B_{z}$ in the near Earth tail and at geosynchronous distances. CMFD has been proposed by Hsieh and Otto (2014) and Otto et al. (2015). A similar process using the Rice Convection Model was described in Yang et al. (2010, 2013). The physical cause for the strong reduction of closed flux in the midnight sector is the transport of magnetic flux to the dayside during periods of southward IMF. This magnetic flux circulation converges toward the dayside magnetopause and is divergent in the near Earth tail (see Fig. 17.3 in Otto et al. 2015).

**Fig. 8:**
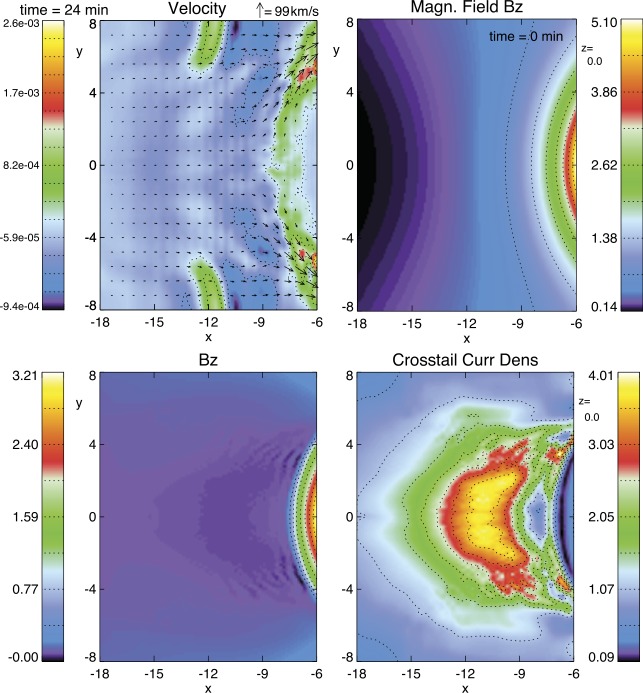
Magnetic flux transport from the night- to the dayside after the IMF turns southward. Top: Velocity (at $t = 25~\mbox{min}$, in units of $430~\mbox{km}/\mbox{s}$) and initial magnetic field component $B_{z}$ (in units of 20 nT) in the equatorial plane. Bottom: $B_{z}$ and cross-tail current density (in units of $2.5~\mbox{nA}/\mbox{m}^{2}$ at $t = 50~\mbox{min}$). Distances are in $\mbox{R}_{E}$ (adapted from Hsieh and Otto 2014 and Otto et al. 2015)

Figure 8 illustrates basic results from three-dimensional meso-scale MHD simulations (Hsieh and Otto 2014; Otto et al. 2015) of a section of the magnetotail (−5 to $-45\,\mbox{R}_{E}$ in $x$, −15 to $+15\,\mbox{R}_{E}$ in $y$, 0 to $12\,\mbox{R}_{E}$ in $z$). The initial configuration uses an appropriate Tsyganenko magnetic field model T96 (Tsyganenko 1995), which is relaxed to an equilibrium configuration (Hall 2006) using a frictional relaxation method (Hesse and Birn 1993). The highest numerical resolution is about $60\text{ km}$ close to the Earthward boundary, and all boundary conditions are fixed except for the sunward boundary. At this boundary an azimuthal sunward flow is prescribed corresponding to a convection channel with a radial width of $\sim2\,\mbox{R}_{E}$ and a radial distance of about $10\,\mbox{R}_{E}$ consistent with flux tube entropies at the dayside magnetopause. Magnitude and profile of this sunward velocity are chosen consistent with typical values of the cross-polar cap potential during the growth phase. The example in Fig. 8 uses a potential of about $50~\mbox{kV}$ (Otto et al. 2015).

After the sunward outflow is applied, the simulation develops a slow divergent convection (away from the midnight sector) in the near Earth tail (top left in Fig. 8). There is also very slow convection toward the equatorial plane (bluish colors in the plot) indicating the thinning of the CS. Consistent with the divergent magnetic flux transport, $B_{z}$ decreases in a large vicinity of local midnight (about 3 hours on either side) from about 25 nT initially to values of less than 1 nT after 50 minutes (Fig. 8) and the decrease is progressing into the initially dipolar region. The strong decrease of $B_{z}$ is associated with the evolution of a very thin and intense crosstail current (Fig. 8, bottom right). The current density is initially reduced earthward of 8 or $9\,\mbox{R}_{E}$ and intensifies tailward of this region. Subsequently an embedded thin CS develops with a half-width of about $0.15\,\mbox{R}_{E}$ at 50 minutes into the evolution. The CS is bifurcated at the Earthward edge. The current density of the thin CS increases to about $10~\mbox{nA}/\mbox{m}^{2}$ or 4 to 6 times the initial value at $t = 50~\mbox{min}$. These results agree well with observational studies relating the decrease of Bz and increase of the current density (Petrukovich et al. 2007; Artemyev et al. 2016b). CS location and the narrow transition to the dipolar region are consistent with recent THEMIS events (Zhou et al. 2009; Kubyshkina et al. 2011; Sergeev et al. 2011).

The time scale for TCS formation is determined by the amount of available closed flux and the total flux transport rate. For about $1.5\times10^{8}~\mbox{Wb}$ of closed flux, a 50 kV potential yields a time of about 45 minutes to deplete this tail section of all magnetic flux. The example also illustrates that the flux transport cannot occur in a steady state because this contradicts $\partial\mathbf{B}/\partial t=0$.

**Fig. 9:**
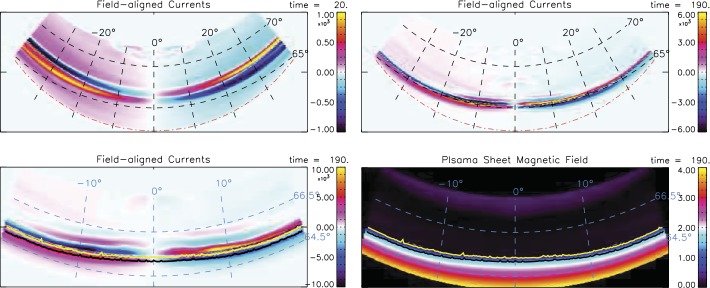
Top: FAC density mapped into the auroral ionosphere at $t=6$ and 50 minutes. Bottom: Magnified view (64.5^∘^ to 66.5^∘^ magnetic latitude) of the FAC density and of the mapped plasma sheet magnetic field $B_{z}$ combined with the 20 keV ion (black line) and 100 keV electron (yellow) isotropy boundaries at $t=50$ minutes (Hsieh and Otto 2014). The time unit corresponds to $\approx 15.7\text{ s}$

CMFD is of major importance for the auroral ionosphere. The magnetic foot points of magnetotail structure such as field-aligned currents, open-closed boundary, isotropy boundaries in the magnetotail (Sergeev et al. 1993a; Newell et al. 1998) are expected to converge in latitude and to move generally equatorward. Hsieh and Otto (2014) have mapped CS properties, field-aligned current density, and particle isotropy boundaries for the near Earth CS evolution into the ionosphere. Figure 9 shows field-aligned current densities after 5 and 50 minutes (top) and higher resolution field-aligned current density and a map of the plasma sheet magnetic field strength after 50 minutes (bottom). The bottom plots also indicates particle isotropy boundaries (yellow and black lines). The results in Fig. 9 demonstrate that the strong reduction of closed flux can explain the typical equatorward motion of the open flux boundary and of the growth phase aurora by 2 to $3^{\circ}$ in latitude. They also show a strong convergence and concentration of the FAC systems, and the proximity of the intense FACs with particle isotropy boundaries and with the sharp transition from stretched to dipolar magnetic field.

While lobe flux accumulation and CMFD represent two separate mechanisms, in reality, they can operate simultaneously. This possibility was considered by Hsieh and Otto (2015) who found both processes highly efficient for generation of TCSs. Absent any other source of compression, it was found that TCS formation by OMFA is also determined by the total amount of flux added to the lobes. When both processes, CMFD and OMFA, operate simultaneously, either one very extended or two TCSs form. Here the relative amount of flux transported in CMFD and OMFA determines which of these CSs evolves faster. The location for TCS formation in OMFA was approximately the same for a uniform and a localized profile of the driving electric field at the lobe boundary. The results suggest that TCS formation can occur simultaneously in different regions of the magnetotail for typical southward IMF conditions.

*Key points:*

(1) Closed magnetic flux depletion, provided by the transport of magnetic flux to the dayside during periods of southward IMF, represents a second major mechanism of the TCS formation and magnetic field line stretching in the near Earth region. (2) In the CMFD process, field-aligned currents, open-closed boundary and isotropy boundaries in the magnetotail converge in latitude and move equatorward, consistent with observations. (3) In reality, CMFD and OMFA are likely to operate simultaneously but can cause TCSs in different locations in the tail.

*Open questions:*

How does CS thinning evolve for different azimuthal and lobe driving conditions?

What is the effect of pre-conditions (amount of closed magnetic flux or asymmetry due to dipole tilt)?

What is the influence of Hall physics or ion kinetics on the CS thinning?

### Testing OMFA and CMFD Pre-onset Mechanisms in Global MHD Simulations and Observations

To test the pre-onset configuration changes scenario outlined in Sects. 2.2.1 and 2.2.2, Gordeev et al. (2017b) used the set of Lyon-Fedder-Mobarry (LFM) global MHD (Lyon et al. 2004) numerical simulations, including 16 runs under different driving conditions. These simulations were shown to be in a good agreement with empirical data with respect to the growth phase duration and tail magnetic flux accumulation (Gordeev et al. 2017a). During several tens of minutes of the growth phase, magnetic flux is redistributed significantly in the magnetosphere. The open flux in the magnetotail lobes increases by up to 50–60% (consistent with empirical estimates, see Shukhtina et al. 2014), corresponding to lobe flux accumulation (OMFA) discussed in Sect. 2.2.1.

**Fig. 10:**
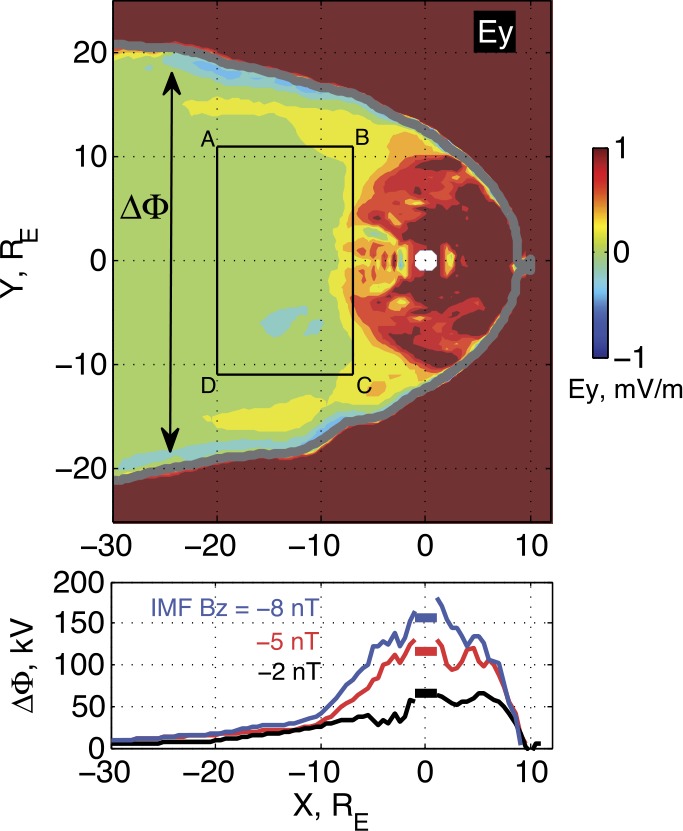
Results of LFM simulation, the end of the growth phase. Top panel: distribution of $y$-component of convective electric field $E_{y}=-(\mathbf{V}\times\mathbf{B})_{y}$ in equatorial plane, which reflects an intensity of sunward magnetic flux transport. Bottom panel: ‘cross-tail electric potential drop’ distribution along $x$-axis, calculated as an integral of convective electric field inside magnetopause boundaries, $\Delta\varPhi= \int_{-MP}^{+MP}(\mathbf{V} \times\mathbf{B})_{y}dy$ as shown in the top panel. Three curves are the results of three simulation runs with identical SW/IMF input except of IMF Bz during the growth phase, which were set to −2, −5 and $-8~\mbox{nT}$. Small rectangular segments shown at $X=0$ are the cross-polar cap potential values

The closed flux in the equatorial magnetotail (for example, threading the contour ABCD in Fig. 10), may decrease several times according to simulation data (Gordeev et al. 2017b), corresponding to the CMFD in Sect. 2.2.2. Global self-consistent simulations reveal that in a few minutes after the sharp increase of the dayside merging rate, strong convection develops in the inner part of magnetosphere ($R<10\,\mbox{R}_{E}$, Fig. 10) starting to sweep out the closed flux toward the dayside. Intensity of this convection as well as the closed flux depletion (CMFD) rate highly correlates with the dayside merging rate. On the contrary, in the midtail the sunward convection stays suppressed during the growth phase (Fig. 10, bottom panel), and it does not respond to significant changes of the dayside driver.

**Fig. 11:**
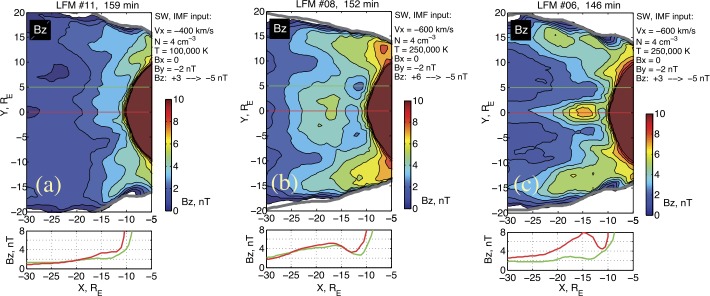
Distribution of equatorial magnetic field in the late growth phase for three LFM simulation runs, which demonstrate different types of global magnetic configuration: (**a**) monotonic radial decreasing of $B_{z}$; (**b**) configuration with local $B_{z}$ minimum; (**c**) $B_{z}$-hump configuration. The simulation input parameters are shown in the right corner of each color panel. Bottom panels show the $B_{z}$ profiles parallel to $X$ axis at $Y=0$ and $Y=5\,\mbox{R}_{E}$, denoted by red and green straight lines in color plots. Adapted from Sergeev et al. (2018)

In addition to other consequences of the CMFD process discussed in Sect. 2.2.2, global MHD simulations reveal its one more interesting feature: the CMFD acts efficiently in the localized distance range at the periphery of the transition region, where it may contribute to the formation of a localized $B_{z}$ minimum (and a tailward $B_{z}$ gradient region). Moreover, global MHD simulations indicate that, depending on the initial configuration and CMFD intensity and configuration, different types of equatorial $B_{z}$ distribution may develop. Particularly, Fig. 11 shows the distribution of equatorial magnetic field at the end of the growth phase for three LFM simulations, which were performed under different SW inputs. In these simulations the equatorial $B_{z}$ demonstrates either monotonic (Fig. 11a) or non-monotonic radial distribution, resembling $B_{z}$ minimum (Fig. 11b) and $B_{z}$ hump (Fig. 11c) configurations which are potentially unstable to interchange or tearing modes. Therefore, both the possibility of forming tailward $B_{z}$ gradient regions and their variability are evident, in agreement with observations (see Sect. 2.2.1).

Due to the CMFD effect, the total magnetic flux in the near-Earth tail cross-section at around $8\mbox{--}12\,\mbox{R}_{E}$ has to increase more slowly than in the mid-tail at $15\mbox{--}30\,\mbox{R}_{E}$. This can be used to test the new growth phase scenario described above and evaluate observationally the CMFD hypothesis. Examining the 15-year period (2001–2015) of joint Cluster and Geotail operation, Shukhtina et al. (2018) found 13 events of isolated substorm growth phases with conjugate spacecraft measurements in the inner and mid-tail distances, suitable for the tail magnetic flux evaluation (Shukhtina et al. 2016). In these events the variation of the total magnetic flux in the two tail cross-sections had similar behavior to that found in simulations (Gordeev et al. 2017b), giving an observational confirmation to the CMFD scenario. Therefore, global MHD simulations as well as spacecraft measurements support the idea that the CMFD process may be an important driver of magnetic reconfiguration in the inner magnetotail.

*Key points:*

(1) Global MHD simulations using the LFM model exhibit both lobe flux accumulation (OMFA) and closed flux depletion (CMFD) in the substorm growth phase. (2) Evaluation of the total magnetic flux using simultaneous spacecraft observations in two tail cross-sections supports the global MHD results. (3) Dependent on the preconditioning and IMF parameters, the closed flux may be redistributed to create monotonic or non-monotonic $B_{z}$ distributions, such as tailward $B_{z}$ gradients.

*Open questions:*

What are the specific factors controlling the range of radial distances where the CMFD process operates?

What are the specific factors (e.g., solar wind/IMF before the southward IMF $B_{z}$ turning) leading to the equatorial $B_{z}$ configurations with and without a tailward gradient $B_{z}$ region?

### Grad-Shafranov Thin Current Sheet Models

The mechanisms discussed in Sects. 2.2.1–2.2.2 operate even under ideal MHD conditions. The resulting TCSs, however, are expected to develop kinetic structures when the thickness approaches or becomes smaller than typical particle scales. In that case the MHD description might break down and Vlasov theory becomes necessary to describe the corresponding self-consistent kinetic equilibria.

One such approach, valid for 2-D equilibria, is based on a generalization of the broad class of isotropic CS models (Schindler 1972). These models use the solution of the Vlasov equation in the form of an arbitrary function of two integrals of motion, the total energy $E_{\alpha}$ of particles of the species $\alpha=i,e$ and the $y$-component of the canonical momentum $P_{y\alpha}(A_{y})$, where $A_{y}$ is the $y$-component of the vector potential. In the original 1972-class models (Schindler 1972) the particle distributions were exponential functions of the invariants resulting in the isotropic distributions (shifted Maxwellians) providing uniform temperatures and charge neutrality. The corresponding Ampere’s equation then transforms into a nonlinear differential equation of the Grad-Shafranov (GS) type (Grad 1961; Shafranov 1958) for the vector potential $A_{y}$.

The GS-type TCS models assume more complex functions of the same invariants $E_{\alpha}$ and $P_{y\alpha}$ (Schindler and Birn 2002). Since all these functions have the dependence on the vector potential $A_{y}$ in the same point, the corresponding Ampere’s and Poisson’s (quasineutrality) equations can be reduced to a similar GS equation: $\nabla^{2}A_{y}=-dp/dA_{y}$, where $p=\sum_{\alpha=i,e} ({p _{\alpha}}_{xx}+{p_{\alpha}}_{zz} )/2$ (Schindler 2007). Moreover, since for these models $j_{y}=dp/dA_{y}$, the force balance equation in the $x$-direction is always reduced to $dp/dx=j_{y} B_{z}$, meaning that the magnetic tension is balanced by the pressure gradient as in conventional isotropic models. The pressure parameter $p$ is mainly determined by ions because of their higher temperature. These TCS models may have temperature gradients, plasma anisotropy and even agyrotropy and they describe both embedded and bifurcated TCSs shown in Fig. 7 (e.g., Birn et al. 2004c). Since plasma parameters $p$, $j_{y}$ and others depend on the vector potential $A_{y}$, these parameters remain constant on the fixed magnetic surface $A_{y}=A_{0}$. In other words, spatial variations in such CS models are transverse to the magnetic field, and the field-aligned gradients are equal to zero everywhere.

*Key points:*

(1) Both embedded and bifurcated TCS can be reproduced in Grad-Shafranov CS models. (2) Spatial variations in such CS models are transverse to the magnetic field allowing no field-aligned gradients. (3) Grad-Shafranov TCSs are relatively short because the magnetic tension is balanced by the pressure gradient and hence large current densities require large pressure gradients along the tail.

*Open questions:*

Are the Grad-Shafranov TCS models sufficient to describe all their observed features?

What are the roles of plasma anisotropy and (ion) agyrotropy in these models?

### Non-Grad-Shafranov Thin Current Sheet Models

Another class of TCS models can be built by replacing or extending the original set of invariants of motion $E_{\alpha}$ and $P_{y\alpha}$. These models utilize the features of quasi-adiabatic ion motion, which allows an additional approximate quasi-adiabatic invariant $I_{z}^{(i)}$ of the particle motion across the sheet (Sonnerup 1971). Assuming anisotropy of the ion species outside the TCS, the magnetic tension in these models can be balanced by the ion inertia when the invariant $I_{z}^{(i)}$ replaces the canonical momentum $P_{yi}$ (Sitnov et al. 2000). The new invariant can also be combined with the original set of the energy and momentum (Sitnov et al. 2003; Zhou et al. 2009; Zelenyi et al. 2011; Sitnov and Merkin 2016). This allows one to describe both embedded and bifurcated TCS (Sitnov et al. 2003; Merkin and Sitnov 2016). An important advantage of the models with the extended set of invariants $E_{\alpha}$, $P_{y\alpha}$ and $I_{z}^{(i)}$ is that they describe plasmas with weak ion anisotropy, including the continuous transition to Harris equilibria (Harris 1962) in the isotropic limit (Sitnov et al. 2003).

**Fig. 12:**
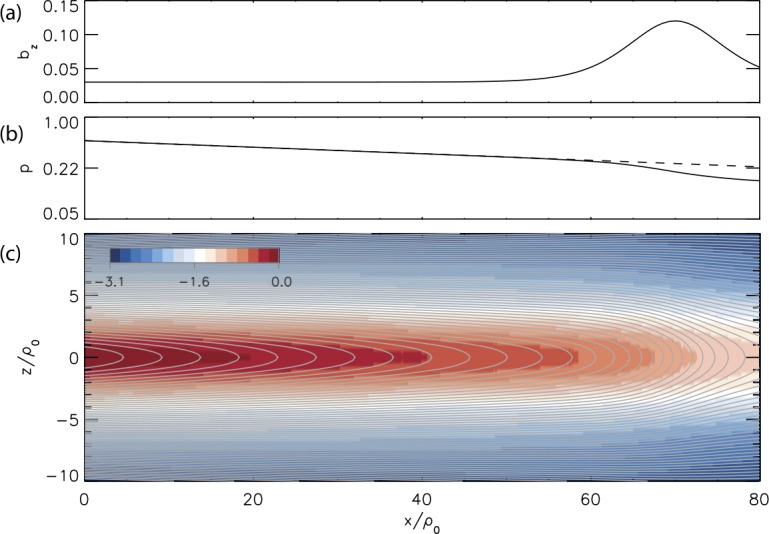
2-D non-Grad-Shafranov TCS equilibrium with the region of accumulated magnetic flux corresponding to the equatorial $B_{z}$ field (4) with the parameters $\xi=x/\rho_{0}$, $\varepsilon_{1}=0.03$, $\varepsilon_{2}=0.133$, $\alpha=3$ and $\xi_{0}=70$. $\rho_{0}$ is the thermal ion gyroradius in the asymptotic field $B_{0}(x=0)$. Panels (**a**)–(**c**) show the equatorial $B_{z}(x,0)$ profile, the effective pressure parameter $p \propto B_{0}^{2}-B_{z} ^{2}$, and 2-D isocontours of the vector-potential with the color-coded logarithm of the current density normalized by its value in the point $(x,z)=(0,0)$ (Sitnov and Merkin 2016)

TCS models with the invariant $I_{z}^{(i)}$ offer another solution of the $1/f$ CS thinning problem discussed in Sect. 2.2.1. Embedded TCS as well as current density dips in bifurcated TCS with the scales of the order of $\rho_{0i}$ appear in these models due to the properties of the quasi-adiabatic “figure-of-eight” ion orbits, and in particular, their corresponding scales, as discussed, for example, in Zelenyi et al. (2003, Fig. 3). Furthermore, since the dependence of the distribution function (through the invariant $I_{z}^{(\alpha)}$) on the integral of $A_{y}$ over the ion orbit causes violation of the isotropic force balance condition $dp/dx=j_{y} B_{z}$, these models can describe non-zero field-aligned gradients of the current density and pressure. An important advantage of TCS models with the extended set of invariants is that they can describe TCS that are strongly extended along the tail (Sitnov and Merkin 2016) (Fig. 12). This is provided due to another small parameter $\rho_{0i}/L \ll1$, in addition to $B_{z}/B_{0}\ll1$ used in GS-type models, which is introduced by the quasi-adiabatic ion dynamics.

Non-Grad-Shafranov TCS models can also be built assuming anisotropic pressure created by adiabatic electrons with ${p_{e}}_{\parallel} \ne{p_{e}}_{\bot}$ (e.g., Artemyev et al. 2016a, and references therein) and using the electron magnetic moment $\mu=mv_{\bot}^{2}/2B$ as an integral of motion in addition to the total energy and canonical momentum. Since $\mu$ depends on the derivatives of the vector potential $A_{y}$, the plasma pressure, current density and other parameters are not constant on a fixed magnetic surface and may have non-zero field-aligned gradients.

*Key points:*

Non-Grad-Shafranov models (1) describe both embedded and bifurcated TCS with weak ion anisotropy; (2) naturally explain the two-scale structure of the tail current sheet due to characteristic features of quasi-adiabatic “figure-of-eight” ion orbits; (3) explain long TCS because the magnetic tension in these models is balanced by both the pressure gradient and by the ion inertia; (4) assume non-zero field-aligned gradients of plasma parameters.

*Open questions:*

What are the impacts of anisotropic electrons and agyrotropic ions on the observed TCS structure?

What the roles of adiabatic, quasi-adiabatic and chaotic regimes of particle motion in the observed TCSs?

## Onset Mechanisms

Plasma modes responsible for onset of magnetotail dipolarizations and affecting global configuration of the magnetotail can be split into three major types: (1) reconnection or tearing-type modes, (2) ballooning/interchange perturbations and (3) flapping motions. Tearing modes are associated with the current filamentation along the tail and they eventually result in topological changes of the tail magnetic field (X-line formation). B/I modes describe buoyancy motions bringing flux tubes with reduced plasma content toward the Earth and are structured in MLT. Flapping motions are also structured in MLT but they represent a different type of motions, global north-south oscillations of the CS as a whole like a flapping flag.

One of the most plausible mechanisms of magnetotail activity is magnetic reconnection. However, a fundamental problem is that the corresponding tearing instabilities (Coppi et al. 1966; Schindler 1974) are almost fully prohibited. As was shown by Lembege and Pellat (1982), due to the stabilizing effect of electrons magnetized by the $B_{z}$ field, the region where tearing is forbidden extends from global to micro (electron gyroradius) scales:

1 $$ \pi (B_{z}/B_{0} )C_{d}^{2} \lesssim kL_{z} \lesssim (B_{z}/B_{0} ) (L_{z}/\rho_{0e} ) $$

Here $k$ is the mode wave number, $L_{z}$ is the current sheet half-thickness, $\rho_{0e}$ is the thermal electron gyroradius in the field $B_{0}$ outside the sheet and $C_{d}=VB_{z}/(\pi L_{z})$, where $V=\int dl/B$ is the flux tube volume. At micro-scales, the right hand side of (1) allows an instability when the electrons become unmagnetized by the field $B_{z}$ (EDMR regime). In this regime inverse Landau damping on the electrons is possible, and this provides the dissipation to drive a collisionless tearing instability. The left hand side of (1) controls another transition to a tearing instability that is possible on macro-scales. This new instability regime requires special magnetic flux distributions in the tail that possess a region of tailward gradient of the field $B_{z}$ (IDMR regime). Such a tailward gradient is not a common feature in the quiet magnetotail configuration that typically possesses only an earthward gradient.

### Tearing Instability

To lowest order, the magnetotail current sheet configuration resembles the classical Harris (Harris 1962) neutral sheet in which the magnetic field $B_{x}(z)$ reverses from an anti-earthward direction in the southern lobe to an earthward direction in the northern lobe, with the transition occurring over a characteristic half-width $L$. It was proposed very early in the space era (Coppi et al. 1966) that collisionless reconnection could occur in this tail current sheet as a result of an electron tearing instability driven by the electron Landau dissipation. The suggestion was that this instability could serve as the triggering mechanism that powers the sudden onset of magnetic reconnection associated with the expansion phase of substorms in the magnetotail.

This simple one-dimensional picture of the magnetotail must fail, however, inasmuch as the magnetic field lines must connect to the intrinsic dipolar magnetic field of the Earth. This results in a small northward component of the magnetic field in the region of the current sheet whose magnitude is typically a few nanoteslas, which is about 10% of the asymptotic (lobe) field strength (e.g., Fairfield and Ness 1970). The presence of this normal magnetic field component has profound implications for the possibility of magnetic reconnection in the tail. On the most fundamental level, the resulting cyclotron motion of electrons in even a very weak normal field removes the electron Landau resonance (Galeev and Zelenyi 1976), thus ruling out the possibility of an electron tearing mode.

The tearing hypothesis for the magnetotail was resurrected by Schindler (1974) who suggested that ion Landau damping could drive a pure ion tearing instability in which the electron dynamics was presumed to be unimportant due to the small value of the electron temperature ($T_{e}/T_{i} \ll1$). He noted that the characteristic scaling of the ion tearing growth rate in the absence of the normal field, valid for $\rho_{i0}/L \ll1$, would be of the form (Laval et al. 1966):

2 $$ \gamma/\varOmega_{i0} \sim(\rho_{i0}/L)^{5/2}, $$

where $\rho_{i0}$ $(\varOmega_{i0})$ is the ion gyroradius (cyclotron frequency) in the asymptotic field $B_{0}$. During quiet times, the ratio $\rho_{i0}/L \sim0.03$. Thus the scaling in (2) would give $\gamma/\varOmega_{i0} \sim2 \times10^{-4}$ or $1/\gamma\sim$ 1 hr, which is too long to be relevant to substorm onset times. It was expected, however, that as the current sheet thinned during the growth phase, the growth time for the tearing instability would decrease substantially. This is particularly significant since it was expected that the condition $\gamma/\varOmega_{i0} > B_{n}/B_{0}$ would need to be satisfied in order that the free-streaming particle motion which drives the tearing mode would not be destroyed by the gyromotion in the normal field $B_{n}$. This expectation was confirmed by particle-in-cell (PIC) simulations of the pure ion tearing mode in a TCS (Pritchett et al. 1991). The scaling (2) then suggests that for $\rho_{i0}/L \approx1$, this condition easily would be satisfied for $B_{n}/B_{0} \sim0.1$.

Subsequent investigations, however, showed that the basic reconnection growth rate increases much less rapidly as $\rho_{i0}/L \rightarrow1$ than suggested by the scaling (2) (Pritchett et al. 1991; Brittnacher et al. 1995). In particular, it was found that for $\rho_{i0}/L = 1$, the maximum growth rate for the pure ion tearing mode (equivalently, $m_{i}/m_{e} = 1$) is only $\gamma_{\mathrm{max}}/\varOmega_{i0} \approx0.17$, about a factor of 6 smaller than expected from (2).

When the effect of the electron dynamics is no longer neglected, an additional barrier to ion tearing arises. Traditionally, the analysis of electron stabilization for the ion tearing instability has been carried out using an idealized 2-D plasma sheet configuration in the noon-midnight meridional ($x,z$) plane; no variation in the $y$ direction is considered. In such a configuration and assuming a tearing perturbation $A_{1y} = A_{1}(x,z) e^{\gamma t}$ and an electrostatic potential $\varPhi_{1} = \varPhi_{1}(x,z) e^{\gamma t}$, the completely general energy principle is (Laval and Pellat 1964; Schindler 1966)

3 $$ \delta W = \int d^{3}x \biggl[ \vert{\mathbf{\nabla}} A_{1} \vert ^{2} - \frac{4\pi}{c}\, \frac{dJ_{0}}{dA_{0}} \vert A_{1} \vert^{2} + \sum _{\alpha=i,e} 4 \pi T_{\alpha} \int d^{3}v \frac{\vert \tilde{f}_{1\alpha} \vert^{2}}{f_{0\alpha} } \biggr]. $$

Here, $f_{0\alpha}$ is the equilibrium distribution function for the species $\alpha$ with the temperature $T_{\alpha}$, $J_{0}$ is the equilibrium current density, and $\tilde{f}_{1\alpha} = f_{1\alpha} -A_{1} \partial f_{0\alpha}/\partial A_{0}$ is the non-adiabatic part of the perturbed distribution $f_{1\alpha}$. The first term in (3) represents the stabilizing effect of field-line tension, the second term is the destabilizing free energy associated with the adiabatic response to the equilibrium currents, and the last term represents the compressibility effect arising from the perturbed current density due to $\tilde{f}_{1\alpha}$. Various assumptions have been made regarding the nature of the electron dynamics.

Lembege and Pellat (1982) used a drift-kinetic analysis (which should be valid for time and space scales long compared to the electron cyclotron period and electron Larmor radius) and assumed adiabatic motion for the electrons. They demonstrated that the tearing mode electromagnetic field produces a strong compression of the electron density which is independent of $T_{e}$. This perturbation also forces a large electrostatic potential in order to maintain charge neutrality. In the energy principle (3), the energy associated with the electron compression exceeds the free energy available from the reversed magnetic field configuration provided that the condition on left hand side of (1) is satisfied. In order to violate it and thus to permit instability, the wavelength of the mode in case of $B_{z}=\mbox{Const}$ would have to exceed $\sim60~\mbox{L}$. On such a large scale the conditions necessary for the WKB approximation to be valid would be violated, and so Lembège and Pellat concluded that the ion tearing mode was stable.

Pellat et al. (1991) avoided any specific assumption regarding the nature of the electron dynamics by appealing to the conservation of the canonical momentum $P_{y}$ in a 2D system. This alone is sufficient to constrain the cyclotron excursion in the direction transverse to the magnetic surface and to preserve the perturbed number of particles on a flux tube. They then recovered the Lembège-Pellat stability criterion (1) under the very mild assumption that $k_{x} \rho _{en} < 1$, where $\rho_{en}$ is the electron Larmor radius in the $B_{n}$ field. Assuming the proton/electron value for the mass ratio, $T_{i}/T_{e} \approx7$, $\rho_{i0}/ L \sim1$, and a wavenumber $k_{x} L \approx0.5$, one finds that this condition is satisfied for a normal field of only $B_{n}/B_{0} \sim5 \times10^{-3}$. Direct confirmation of this electron stabilization effect was provided by 2-D PIC simulations (Pritchett 1994; Dreher et al. 1996).

Additional investigations using various assumptions have all recovered the stabilization result (3). Brittnacher et al. (1994) examined the cases of intrinsic and external pitch-angle diffusion and found at most an additional stabilizing term, so that the marginal stability criterion remained the same. Quest et al. (1996) employed fluid equations to evaluate the perturbed particle density assuming that the electrons were frozen-in to the magnetic field, demonstrating that the electron stabilization is a macroscopic fluid effect, independent of the specifics of the electron orbits. Schindler (2007) presented an alternative Vlasov treatment that generalized the class of distribution functions beyond simply drifting Maxwellians, avoided the use of inequalities, and introduced the small electron gyroscale regime by considering the formal limit $m_{e} \rightarrow0$.

Thus, the possibility of a collisionless tearing instability is problematic due to two issues: (1) the need to have a thin enough current sheet so that the tearing growth rate is faster than the ion gyrorotation time, and (2) the stabilizing effect of the electron compressibility needs to be reduced or eliminated. Of these two, the electron compressibility appears to present the greatest impediment.

### Electron Demagnetization-Mediated Reconnection

One way to achieve the tearing destabilization of the tail is to account for the magnetic flux addition to the lobes during the substorm growth phase, which causes a compression and a reduction of the thickness parameter $L$ below the ion inertial length $d_{i}$ and the magnetic field $B_{z}$ to break the stability condition on the right hand side of (1). This was first demonstrated by Pritchett and Coroniti (1995) in a 2D magnetotail and later by Pritchett (2005) in a 3-D PIC simulation with mass ratio $m_{i}/m_{e} = 100$. In these simulations, a persistent strong electric field was applied at the high-latitude boundaries to a moderately thick ($L = 1.6 d_{i}$) current sheet containing a nonzero $B_{z} = 0.04 B_{0}$. The external driving led to a significant thinning of the current sheet and a concomitant increase in the underlying tearing growth rate. Yet as the sheet became thin enough to allow for an ion tearing mode, there was no apparent increase in the reconnected flux. It was only as $B_{z}$ approached zero and the electrons became unmagnetized that a marked increase appeared in the reconnected flux. Thus, the strong driving regime appears to initiate reconnection without the presence of a linear instability stage.

**Fig. 13:**
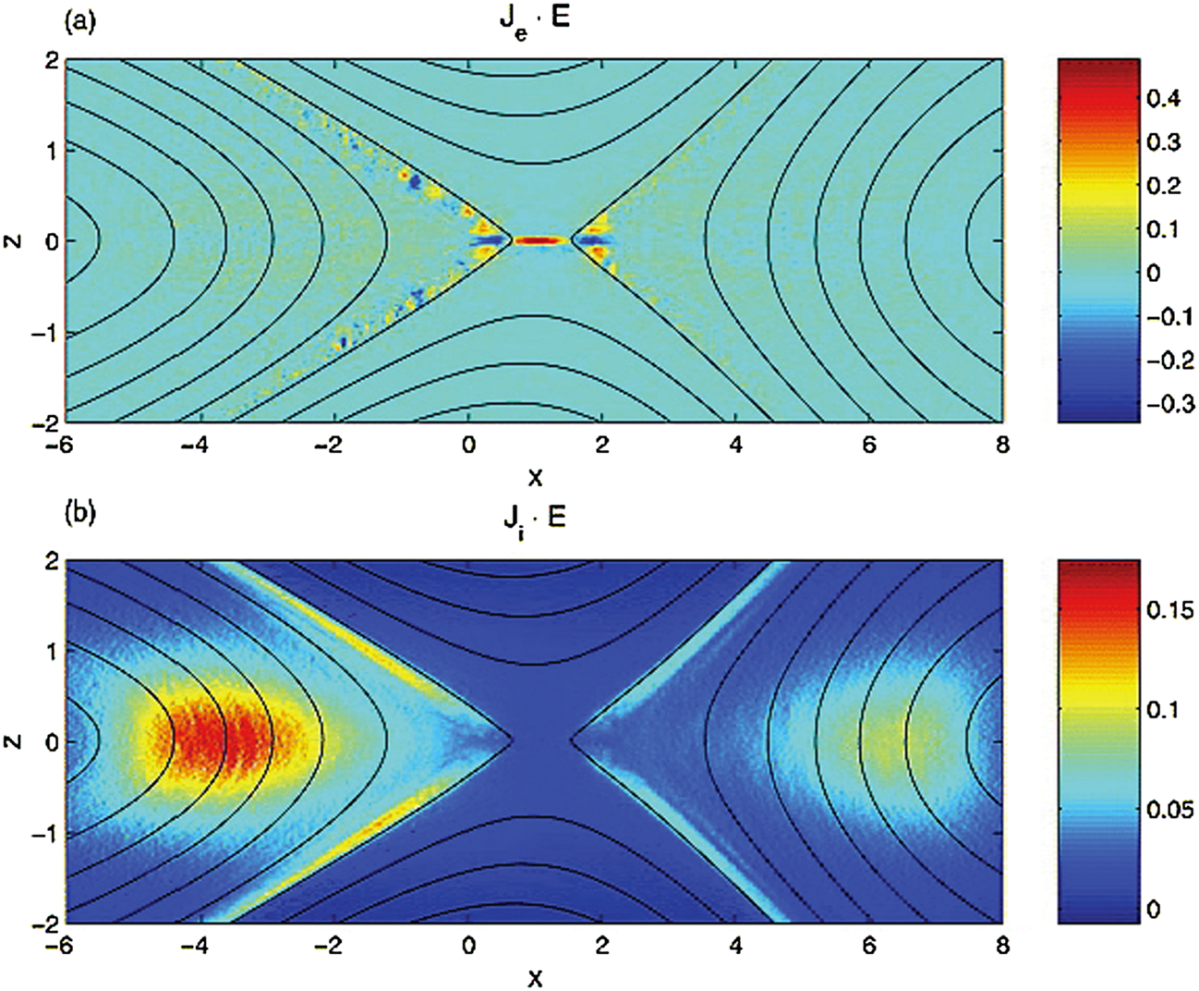
Distributions of the load factor $\mathbf{j} \cdot{\mathbf{E}}$ from a 2D PIC simulation with $m_{i}/m_{e} = 1600$ (Pritchett 2010): (**a**) electron contribution and (**b**) ion contribution. Black lines show isocontours of the $y$ component of the vector potential, and spatial coordinates are normalized by $d_{i}$

The externally-driven 2D simulations were extended by Pritchett (2010) to treat mass ratios of $m_{i}/m_{e} = 400\mbox{ and }1600$. The time behavior of the reconnected flux (expressed in terms of the ion gyrofrequency) was virtually identical over the entire mass ratio range from 25 to 1600. Thus, the onset did not involve an electron tearing instability. Nevertheless, the electron dynamics was clearly responsible for the reconnection. Figure 13 shows the load factor $\mathbf{j} \cdot{\mathbf{E}}$ for the $m_{i}/m_{e} = 1600$ simulation associated with (a) the electrons and (b) the ions. The dominant contribution comes from the electrons and is concentrated in a very thin layer of width of the order of the local electron inertia length where the electrons become demagnetized. The somewhat weaker (and frame-dependent) ion contributions to $\mathbf{j} \cdot \mathbf{E}$ in the expanding fronts both earthward and tailward of the X-line represent a transfer to thermal energy via $\mathbf{U_{i}} \cdot {\mathbf{j \times B}}/c$ and are not a true dissipation (Birn and Hesse 2005; Goldman et al. 2016). This example of an externally-driven EDMR provides a self-consistent realization—without the initial lack of pressure balance—of the original GEM (Geospace Environment Modeling) Challenge regime (Birn et al. 2001).

While the simulations of Pritchett (2005, 2010) were based on persistent driving at the high-latitude boundaries, an alternative approach was taken by Hesse and Schindler (2001), Birn and Hesse (2014), and Y.-H. Liu et al. (2014b) using 2D PIC simulations. This approach was made, consistent with predictions from quasi-static, adiabatic equilibrium theory (Birn and Schindler 2002), on the basis that even moderate finite perturbations at the high-latitude boundaries could cause local thinning, current intensification and reduction of $B_{z}$, and ultimately even a loss of equilibrium (see Sect. 2.2.1).

Y.-H. Liu et al. (2014b), in particular, demonstrated that the response of the magnetotail to the finite boundary deformation (at least for slow driving) depended on the amplitude of the vector potential deformation, rather than the magnitude of the driving electric field. They found a threshold, below which the configuration settled into a new equilibrium with an embedded intensified, yet stable, current sheet. If the deformation amplitude exceeded that threshold, the system would undergo the onset of instability, tearing, reconnection, and plasmoid formation and ejection. By varying the assumed ion to electron mass ratio from 25 to 100, 400, and up to the real proton/electron mass ratio, it was found that the onset of the instability (determined by observing a marked change in slope of the rate of decrease of the $B_{z}$ field) clearly depended on the value of the mass ratio. This indicates that the onset of reconnection in a sufficiently thinned initial sheet involves the electron rather than the ion tearing mode. For ion tearing, there would be no such dependence.

The Y.-H. Liu et al. (2014b) studies also demonstrated the governing characteristics of the various stages of the evolution. The early evolution, in response to the boundary deformation, prior to the onset of instability, was basically identical for different mass ratios, consistent with PIC simulation results of Pritchett (2010) and an ideal MHD evolution. This was confirmed in particular by the conservation of the entropy integral (see Sect. 2.2.1), which is imposed in ideal MHD by the adiabatic approximation (see also Birn and Hesse 2014). As discussed above, the onset of instability, which occurred prior to the formation of an $x$-line, was clearly mass-dependent, which was also true for the immediate evolution after onset. It is noteworthy that the $x$-line and plasmoid formation is a consequence of the tearing instability, rather than a condition for its onset. In contrast, the subsequent growth, marked by the most rapid increase in reconnected flux and the maximum reconnection electric field appeared independent of the mass ratio and the related dissipation mechanism. This indicates that this phase is dominated by ion dynamics, which is consistent with the results of the GEM Challenge studies (Birn et al. 2001).

**Fig. 14:**
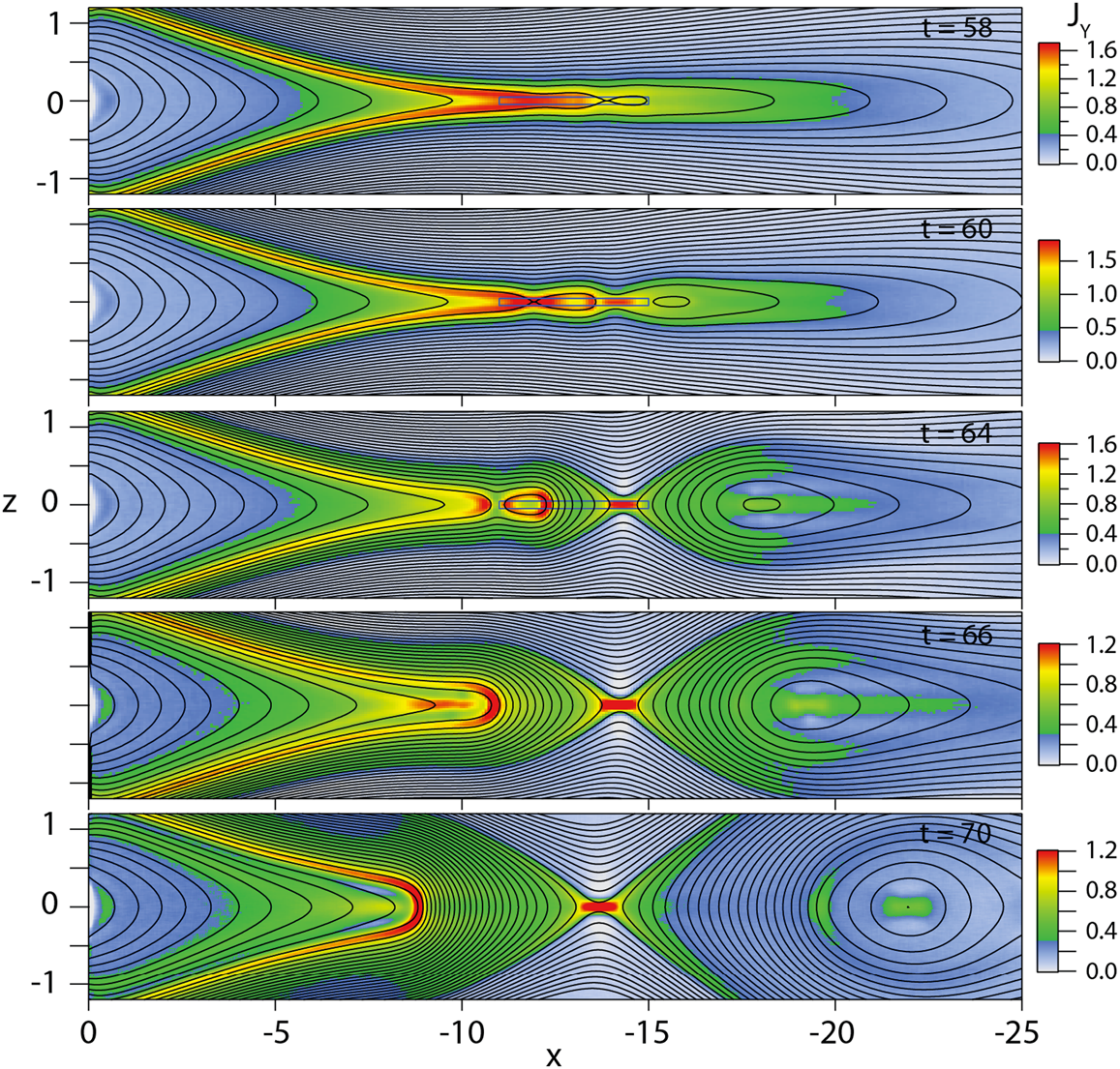
Distributions of the current density $J_{y}$ in 2-D PIC simulations of the EDMR regime around the reconnection onset obtained from run 4 in Y.-H. Liu et al. (2014b) with $m_{i}/m_{e}=1836$. Black lines show isocontours of the $y$ component of the vector potential. The electron tearing-unstable CS is obtained in this run by applying and an external electric field to the 2-D generalized Harris equilibrium during a period $\sim10\varOmega_{ci}^{-1}$. Spatial coordinates are normalized by $d_{i}$

Characteristic stages of the evolution of the $m_{i}/m_{e}=1836$ case in the Y.-H. Liu et al. (2014b) studies are illustrated in Fig. 14 (data courtesy of W. Daughton). The Figure shows snapshots of magnetic field lines and of the color-coded current density $J_{y}$ after the onset of instability, which was identified near $t=56$ (times are in units of $1/\varOmega_{ci}$ and lengths in units of the ion inertial length $d_{i}$). The system size for this simulation was $60\times20$ in $x,z$ with $6144 \times3072$ grid cells and $\sim5\times10^{9}$ particles per species. The evolution shows the initial current concentration on the electron scale, which extends to bifurcated current sheets toward the left (earthward), a relatively short electron current sheet around the $x$-type neutral line, the temporary formation of a small island or flux rope structure and the formation and earthward propagation dipolarization front (vertical current sheet), all on electron scales.

**Fig. 15:**
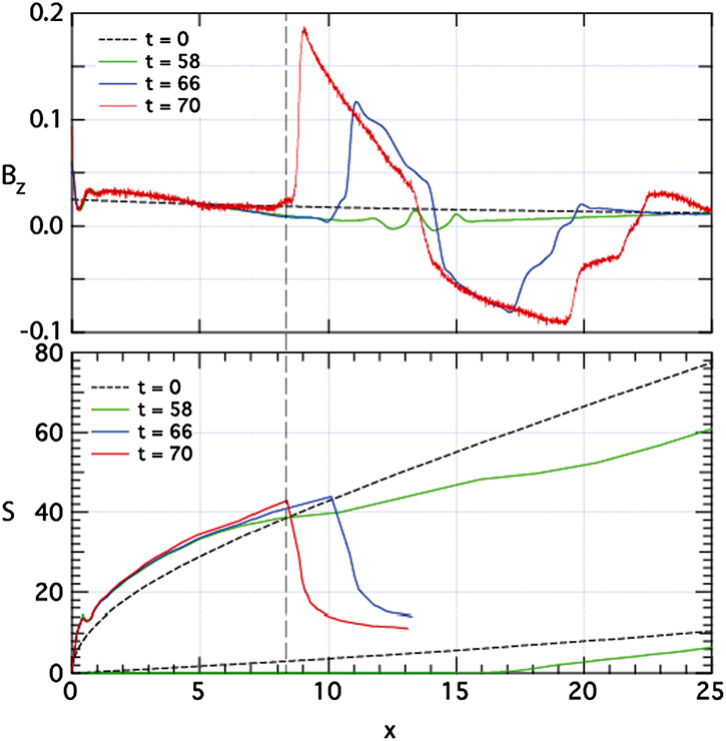
Evolution of the magnetic field component $B_{z}$ (top) and the entropy integral $S$ (bottom), as function of $x$ for the simulation shown in Fig. 7 (run 4 of (Y.-H. Liu et al. 2014b) with $m_{i}/m_{e}=1836$). The dashed lines show the initial variation, the green lines show the variation at a time just after the identified onset of electron tearing, and the red and blue curves the variations at later times, demonstrating the earthward propagating DF

Figure 15 shows the evolution of the magnetic field component $B_{z}$ (top) and the entropy integral $S$ (bottom), defined as $S=\int p^{1/\gamma}dV$, and plotted as a function of $x$ for the same value of $m_{i}/m_{e}=1836$ used in simulation. The dashed line shows the initial variation; the monotonic increase of $S$ with distance downtail illustrates the initial stability to ballooning modes. The green lines show the variation for a time just after the identified onset of electron tearing; the waviness of $B_{z}$ between $x\sim12$ and $x\sim15$ indicates the early mode structure, akin to Fig. 5 of Y.-H. Liu et al. (2014b). It is noteworthy that at this time the entropy integral is still monotonically increasing with distance downtail. At later times (red and blue curves), the $B_{z}$ variation demonstrates the sharp, earthward propagating, dipolarization front (DF), shown also in earlier simulations. From the comparison with the entropy variation, it is obvious that the DF and the following region of enhanced $B_{z}$ consist of reconnected flux tubes, whose entropy content has been reduced by the plasmoid severance, rather than a pile-up of the unmodified medium earthward of the front.

The development of the region of enhanced $B_{z}$ and reduced entropy $S$ with a strong tailward gradient of $B_{z}$ is remarkable also for a second reason. As we will discuss in the following section, this type of equilibrium configuration may be subject to a fast growing ion dominated instability. Although the configuration that leads to this development is not in exact equilibrium, one might consider the transition from (a) relatively slow, electron tearing mode to a faster evolution to be associated with this ion mode.

**Fig. 16:**
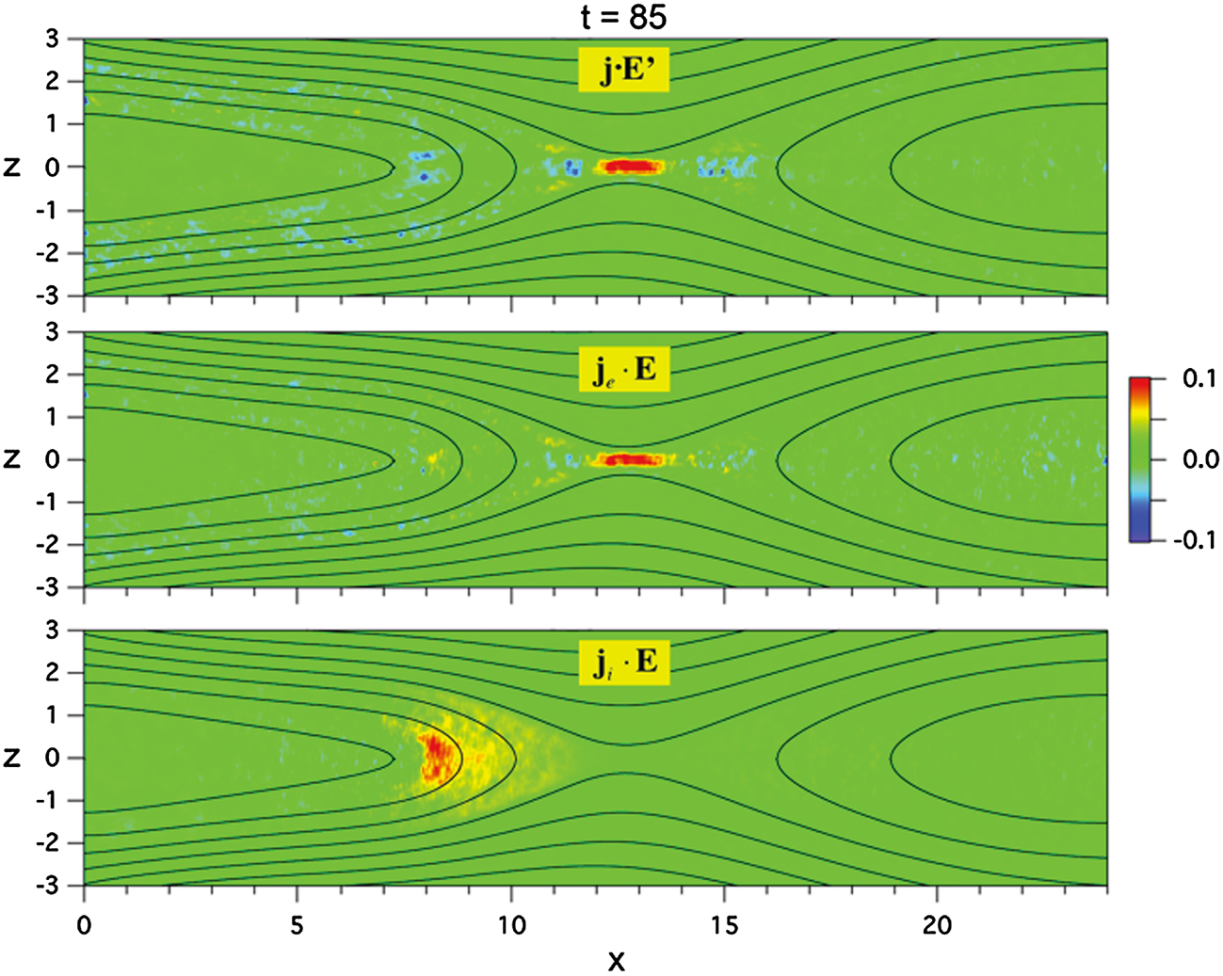
Joule dissipation and energy conversion rates to the electron and ion species in the EDMR regime (Birn and Hesse 2014)

Magnetic reconnection in the tail was proposed not only as a mechanism of rapid reconfiguration of the magnetic field and flux redistribution, but also as a mechanism of the energy conversion and a source of energetic particles. The energy conversion for the EDMR regime with the constant driving (Pritchett 2010) shown in Fig. 13 was dominated by the electrons in the electron diffusion region (EDR). The EDMR energy conversion picture in case of the limited-time or slow driving (Birn and Hesse 2014) reveals similar enhancement of the energy conversion in the EDR (Fig. 16). In these regimes the energy conversion near DF-like regions outside X-lines is comparable with that in the EDR. However, it mainly goes to the bulk acceleration of the ion species and contributes to the enthalpy flux. This is seen from the distribution of the Joule heating parameter $\mathbf{j}\cdot\mathbf{E}^{\prime}$ (Fig. 16, top panel), where $\mathbf{E}^{\prime}_{\alpha}=\mathbf{E}+\mathbf{v} _{\alpha}\times\mathbf{B}/c$, $\mathbf{v}_{\alpha}$ is the bulk flow velocity of the species $\alpha$ and due to the quasi-neutrality $\mathbf{j}\cdot\mathbf{E}^{\prime}_{i}=\mathbf{j}\cdot\mathbf{E} ^{\prime}_{e}$.

The most recent observations (Torbert et al. 2018) made by the MMS mission confirmed that the EDR indeed forms in the magnetotail, and its properties, including the electron-scale thickness of the CS, are consistent with earlier theoretical models. They have also brought new challenges and showed in particular that the conventional resistive-MHD measure of dissipation, $\mathbf{j}\cdot\mathbf{E}^{\prime}$, is difficult to interpret (especially in case of its negative values) and it requires a kinetic generalization suitable for collisionless plasmas (see the next section for more detail). Further studies (Stawarz et al. 2018; Teh et al. 2018) revealed several ion-scale flux ropes near the EDR. This is qualitatively consistent with the development of the electron tearing mode within the EDMR regime (Y.-H. Liu et al. 2014b), the specific scales ($\ll d_{i}$ in case of the electron tearing and $\sim10 d_{i}$ in observations) appear to be more consistent with the oblique secondary tearing modes found in turbulent guide-field reconnection regimes (Daughton et al. 2011; Nakamura et al. 2016).

*Key points:*

Key results regarding the initiation of an EDMR regime due to magnetic flux addition to the lobes during substorm growth phase are: (1) Continual flux addition leads to current sheet thinning independent of the value of $m_{i}/m_{e}$, but an increase in reconnected flux occurs only when the electrons become unmagnetized; (2) A finite boundary displacement (up to some maximum) leads to the formation of a new equilibrium with an embedded and intensified (yet stable) current sheet. For larger displacements, a mass-dependent increase in the rate of change in the erosion of the $B_{z}$ field occurs that is indicative of the EDMR.

*Open questions:*

What are the distinctive features of the EDMR in 3D? In particular, is it possible to compress the CS to electron scales in the presence of the sub-proton-scale instabilities such as the lower-hybrid drift instability?

How are the onset criteria affected by the presence of externally imposed or locally generated waves and turbulence?

### Ion Demagnetization-Mediated Reconnection

**Fig. 17:**
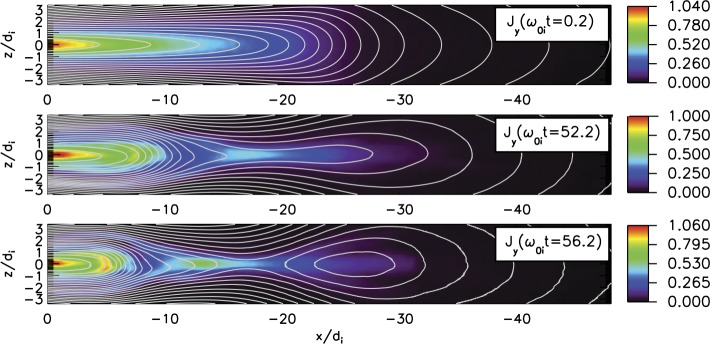
Distributions of the current density $J_{y}$ in the meridional plane $y=2.5d_{i}$ at the beginning of 3-D PIC simulations of the IDMR regime, at the time and after the formation of the new X-line behind the DF. White lines show isocontours of the $y$ component of the vector potential. Parameters of the run are similar to those used in Sitnov et al. (2017), including $m_{i}/m_{e}=128$, with the main distinctions in the CS thickness $L=1d_{i}$ and the box size dimensions $L_{x} \times L_{y} \times L_{z} =80d_{i} \times10d_{i} \times10d _{i}$

As was first noticed by Sitnov and Schindler (2010), a possibility for breaking the stabilization of tearing (Sect. 3.1) and thus allowing spontaneous reconnection in the tail CS with magnetized electrons appears because the left hand side of the stability condition (1), derived by Lembege and Pellat (1982), differs from the WKB condition by the factor $C_{d}$. It may be greater than one in the regions with a tailward $B_{z}$ gradient. As is seen from Fig. 17, the corresponding ion tearing instability may develop in CS with the thickness $L_{z} > d_{i}$. Simulations have been performed in a 3-D box with dimensions $L_{x} \times L_{y} \times L_{z} =80d_{i} \times5d_{i} \times20d_{i}$. Figure 17 shows the region $-48d_{i}< x<0$ and $|z|<3.375d_{i}$ in the plane $y=2.5 d_{i}$. Simulations based on the explicit massively parallel PIC code P3D (Zeiler et al. 2002) start from a 2-D isotropic plasma equilibrium (Schindler 1972) with the vector potential $\mathbf{A}^{(0)}=(0,-\psi(x,z),0)$, where $\psi=LB_{0}\ln[ \beta(x) \cosh( z/(L\beta(x) ))]$, $L=d_{i}$ is the characteristic current sheet thickness parameter, and the $x$-axis points from Earth to Sun. The tailward gradient in this equilibrium is described by the following analytical model:

4 $$ B_{z} ( x,z=0 ) =\varepsilon_{1}B_{0} \bigl[1+\alpha\cosh ^{-2} \bigl( \varepsilon_{2} ( \xi- \xi_{0} ) \bigr) \bigr] $$

where $\varepsilon_{1}\ll\varepsilon_{2}\ll1$ and $\alpha>0$ are constant parameters that determine the equilibrium vector potential through its key function $\beta ( x ) =\exp ( \varepsilon_{1}g (\xi ) )$, with $\xi=x/L$ and $-g ( \xi ) =\xi+(\alpha/\varepsilon_{2}) [1+ \tanh ( \varepsilon_{2} ( \xi-\xi_{0} ) ) ]$. According to Schindler (1972), $\beta ( x )$ is a slowly varying function, compared to the $z$-dependence of the vector potential $\partial/\partial x \ll \partial/\partial z$. Thus, (4) selects equilibria with a region of accumulated magnetic flux near $\xi=\xi_{0}=-24d_{i}$. The specific values of the parameters $\varepsilon_{1}=0.03$ and $\varepsilon_{2}=0.2$ and $\alpha=3$. Open, periodic and closed boundary conditions are employed, correspondingly, in the $x$, $y$- and $z$-directions (Sitnov et al. 2017, and refs. therein).

Simulations show that the reconnection instability indeed develops in equilibria (4) and it has characteristic signatures of the long-sought ion tearing instability (Schindler 1974). In particular, the spatial structure of the growing electric field (Sitnov and Swisdak 2011, Fig. 14) matches the theory prediction (Pritchett et al. 1991) $E_{y} \propto\cosh^{-kL_{z}}(z/L_{z})$. Furthermore, consistent with the stability theory predictions (Sitnov and Schindler 2010), the mode is stable for $C_{d}=1$, and it becomes progressively more unstable with the increase of that parameter up to $C_{d}=3$ (Sitnov et al. 2013). The destabilization was first demonstrated for simulations with different types of open $x$-boundaries (Sitnov et al. 2013; Bessho and Bhattacharjee 2014) and later confirmed in the most rigorous (from the energy principle point of view) case of closed boundaries (Pritchett 2015). The detailed comparison of the stability theory and 2D PIC simulations results has been provided in Merkin and Sitnov (2016, Table 1).

It should be emphasized here that, because of the finite normal magnetic field component $B_{z}$, the ion tearing instability, which ultimately leads to magnetic reconnection in the tail, must start before the magnetic topology change, i.e., reconnection proper. Thus, the conventional term “ion tearing instability” is rather misleading: no real tearing of magnetic field lines may occur unless electrons become unmagnetized. Therefore, following Merkin and Sitnov (2016), to avoid confusion, we denote this instability a Magnetic Flux Release Instability (MFRI). At the same time, one has to take into account that, as shown in the next section, the MFRI may also develop in ideal MHD, whereas IDMR involves the ion Landau dissipation and eventually provides the topology change. Therefore, the acronym for the specific regime of reconnection, the IDMR, when the dissipation in the MFRI is provided by the ion Landau resonance, will also be used in this section.

**Fig. 18:**
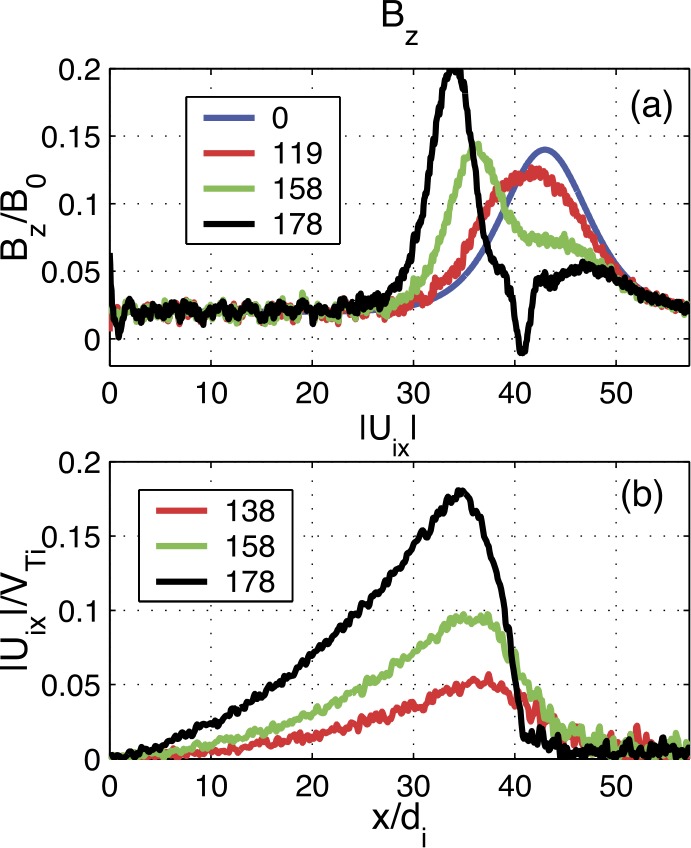
Time evolution of (**a**) the $B_{z}$ profile and (**b**) the bulk ion flow velocity profile $\vert U_{ix} \vert$ in a 2D PIC simulation with closed boundaries and an initial configuration similar to that of (4) (Pritchett 2015, Fig. 3b). Times provided in the insets are given in units of $\varOmega_{i0}^{-1}$ based on the asymptotic $B_{0}$ field at the location of the hump maximum (for more details on normalizations, see Merkin and Sitnov 2016)

**Fig. 19:**
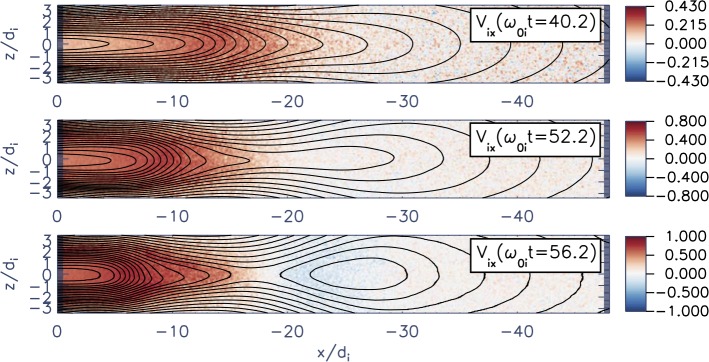
Spontaneous generation of earthward plasma flows in the IDMR regime shown in the form of distributions of the ion bulk flow velocity component $V_{ix}$ in the meridional plane $y=2.5d_{i}$ at different moments for the run shown in Fig. 17. Isocontours of the $y$ component of the vector potential are shown by black lines

The development of the MFRI indeed differs drastically from conventional tearing regimes with the formation of magnetic islands as is the case for the electron tearing instability (Y.-H. Liu et al. 2014b). Figure 18 shows the development of the instability in a simulation with closed boundaries (Pritchett 2015). The instability starts with a coherent earthward (leftward in Fig. 18) displacement of the original region of accumulated magnetic flux (shown by the blue curve in Fig. 18a). As this process continues, there is a build up of the hump above the initial maximum and a corresponding erosion of flux on the tailward (right) side. By the time $\varOmega_{i0}t = 178$, the increase in peak $B_{z}$ reaches 40% above the original maximum, and the tailward edge of the hump approaches and passes through zero due to flux starvation. Once this X-line forms, the system is rapidly disrupted by large-scale reconnection. Prior to this disruption, there is very little change in the leftward half of the $B_{z}$ profile where the field is weak. In simulations with open boundaries (Sitnov et al. 2013, 2014, 2017; Bessho and Bhattacharjee 2014), however, the earthward flows become much pronounced (Fig. 19) and the $B_{z}$ hump increases strongly by factors of two to three to form a pronounced DF prior to the formation of a trailing X-line. The formation of DFs in place of a chain of magnetic islands is a distinctive feature of the MFRI, which was first described in Sitnov et al. (2009) for reconnection outflows with magnetized electrons. At the same time, DFs and earthward ion flows are characteristic for magnetotail activity (Ohtani and Mukai 2008; McPherron et al. 2011; Angelopoulos et al. 2013). Thus, while the properties of the MFRI mode are qualitatively similar in the open and closed simulations, the quantitative details are clearly quite different. This indicates the extreme importance of boundary conditions in quasi-local calculations.

Different motions of ions and more magnetized electrons result in the formation of the in-plane $(x,z)$ Hall currents and out-of-plane magnetic fields $B_{y}$. Their pattern after the formation of a new X-line is similar to the classical quadrupole pattern of anti-parallel reconnection (Sonnerup 1979; Shay et al. 1998). However, since the MFRI starts before the topology change it reveals other Hall patterns, such as the dipole pattern prior to the new X-line formation (Sitnov et al. 2014) and the quadrupole pattern around the DF, which is opposite in polarity to the classical quadrupole around the X-line (Sitnov et al. 2014). The latter was noticed earlier in hybrid and PIC simulations (Nakabayashi and Machida 1997; Zenitani et al. 2013), where it was interpreted as a pileup effect caused by the interaction of the reconnection outflows with ambient tail plasma similar to the snow-plow mechanism discussed by Lapenta and Bettarini (2011). An important new result obtained in PIC simulations of the MFRI (Pritchett 2015; Sitnov et al. 2017) is that the new Hall patterns can be formed before the topology change and can be considered as its precursor signatures.

In contrast to the EDMR, the new X-line is formed in the IDMR regime due to evacuation of magnetic flux by the growing and moving earthward DF structure. In simulations with closed boundaries (Pritchett 2015) this flux starvation results from the fact there is no process to replenish flux that has shifted earthward. However, similar X-line formation processes have also been observed in simulations with open boundaries (Sitnov et al. 2013; Bessho and Bhattacharjee 2014) suggesting that its mechanism is more universal than an effect of the closed boundary. Indeed, it is also similar to the triggering of reconnection in 3D by earthward streaming of the finite-$k_{y}$ B/I heads (Pritchett and Coroniti 2011) as well as the bubble-blob formation process observed in some resistive MHD models (Hu et al. 2011; Yang et al. 2011). This process also resembles generation of magnetic reconnection by shear flow vortices (Liu and Hu 1988), which may be formed because of the Kelvin-Helmholtz instability near the magnetopause.

**Fig. 20:**
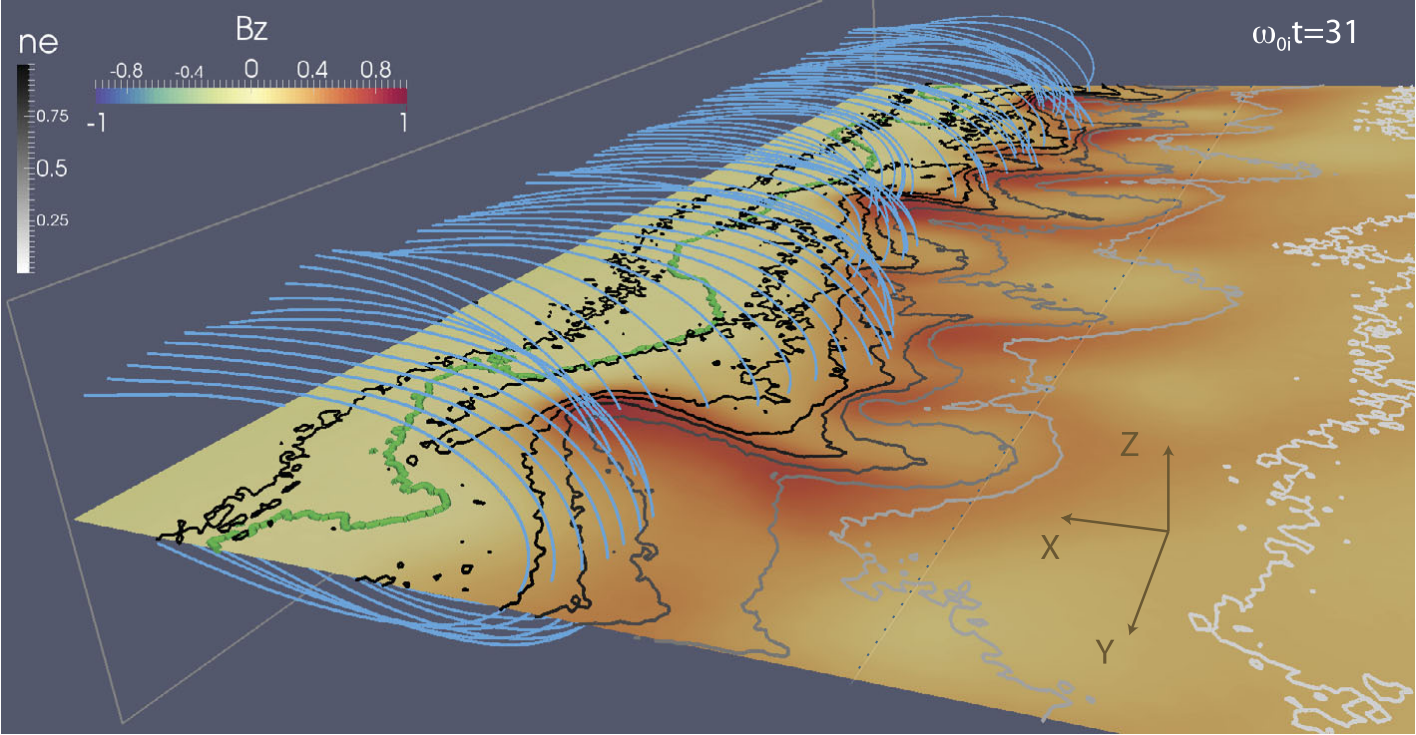
DF perturbed by B/I motions in 3D PIC simulations of MFRI (Sitnov et al. 2014). $B_{z}$ (color-coded) and electron density $n_{e}$ (gray-shaded contours) are shown in the neutral plane. Magnetic field lines (blue) are traced from the line $(x,z)=(-d_{i}, 0.6d_{i})$. The green trace indicates the new O-line (defined as the $B_{z}=0$ contour) forming ahead of the front. The dimensions of the displayed subset of the simulation box are indicated with two rectangles: vertical $x=0$, $y=(0,10d_{i})$, $z=(-d_{i}, d_{i})$ and horizontal $y=(0,10d _{i})$, $z=0$, and $x=(-4d_{i},0)$, where the lower bound is marked by the dotted line

Equilibria with the tailward $B_{z}$ gradient necessary for the IDMR regime are also prone to B/I instabilities (Merkin and Sitnov 2016). Therefore it is important to check their stability in 3D. Dedicated 3D PIC simulations (Sitnov et al. 2014, 2017) show that B/I motions indeed substantially perturb DFs forming due to MFRI. In particular, Fig. 20 shows that regions of the local in $y$- direction enhancement of the field $B_{z}$ correlate with the regions of plasma density reduction. Thus, they have indeed properties of B/I fingers or plasma bubbles (Pontius and Wolf 1990). At the same time, Fig. 20 shows that even in the absence of the background plasma, which should strongly stabilize the B/I (Schindler and Birn 2004; Merkin and Sitnov 2016), interchange motions do not destroy the global 2D structure of reconnection motions provided by MFRI.

As is seen from Merkin and Sitnov (2016), in the IDMR models discussed above the destabilization of the ion tearing is achieved due to the reduction of the stabilizing third term in the energy principle (3). Another way of destabilization was considered by Zelenyi et al. (2008) who suggested that in embedded non-Grad-Shafranov TCS (Sect. 2.5) one can expect the enhancement of the destabilizing second term. The tearing regions were indeed found for models with strong ion anisotropy outside CS. However, neither the possibility of such unstable regions in case of more realistic weak anisotropy ($|T_{||i}-T_{\perp}|\ll T_{\perp }$) nor the actual development of the corresponding instability in PIC simulations have been demonstrated so far. Meanwhile, the importance of TCS in the onset mechanism has recently been confirmed by the data-mining reconstruction of substorms: Stephens et al. (2019) showed that the buildup and decay of embedded TCS is a distinctive feature of substorm growth and expansion phases.

**Fig. 21:**
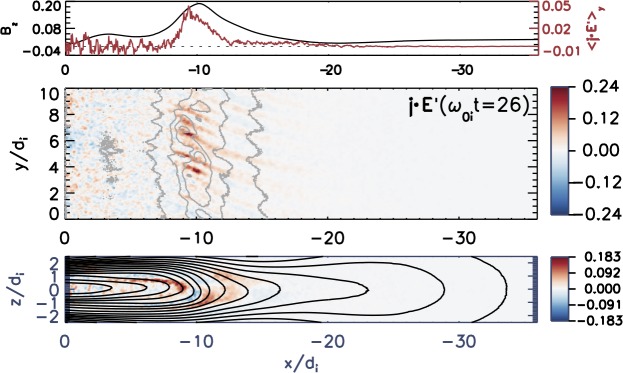
Joule dissipation in the IDMR regime before the new X-line formation (Sitnov et al. 2017). The central and bottom panels show the distribution of the corresponding energy conversion parameter in the equatorial plane $z=0$, in the meridional plane $y=5d_{i}$, while the top panel shows the distribution in the equatorial plane of the energy conversion parameter averaged over the $y$-direction (red) and the similar distribution of the $B_{z}$ magnetic field component (black)

Since in the IDMR regime, the reconnection instability starts before the formation of a new X-line and an EDR, the energy conversion and dissipation (Fig. 21) in that regime is associated with DFs (Sitnov et al. 2017). The magnetic energy is converted largely to the ion species. However, it is also strongly structured on the sub-proton scales with the energy conversion peaks higher for electrons than for ions. Whether this complex energy conversion picture is dominated by the ion Landau dissipation remains unclear because the corresponding Joule dissipation measure (Fig. 21) is essentially a resistive MHD parameter ($\mathbf{j}\cdot\mathbf{E} ^{\prime}\propto(d/dt)(pn^{-\gamma})$), which does not distinguish between the ion and electron dissipation channels.

After the formation of a new X-line the energy conversion and dissipation picture in the IDMR regime becomes more similar to that in case of the EDMR (Sitnov et al. 2017, Fig. 3), including the formation of another dissipation region, the EDR. The latter however remains strongly shifted earthward relative to the X-line. Also, the energy conversion remains dominated by ions at the DF rather than electrons near the EDR.

Energy conversion and dissipation near DFs have already been studied in a number of observations. Strong energy conversion at the fronts was reported by Angelopoulos et al. (2013) and Huang et al. (2015) ($>0.5~\mbox{nW}/\mbox{m}^{3}$ on average of a set of 18 events). The enhancement of the parameters similar to $\mathbf{j}\cdot\mathbf{E}^{\prime}$ were reported by Runov et al. (2011) and Khotyaintsev et al. (2017). At the same time Khotyaintsev et al. (2017) and Yao et al. (2017) reported strong negative peaks of $\mathbf{j}\cdot\mathbf{E}^{\prime}$ ahead of the $B_{z}$ peaks, the effect also seen in 3-D PIC simulations (Sitnov et al. 2017, Fig. 3). The latter finding challenges the MHD parameter $\mathbf{j}\cdot\mathbf{E}^{\prime}$ as a correct measure of the dissipation processes in the collisionless plasmas. Perhaps even more challenging is the fact that the energy conversion rates in the frames moving with ions or electrons $\mathbf{j}\cdot \mathbf{E}_{e,i}^{\prime}$ (where $\mathbf{j}=\mathbf{j}_{i}+ \mathbf{j}_{e}$, $\mathbf{E}_{e,i}^{\prime}=\mathbf{E}+\mathbf{v} _{e,i}\times\mathbf{B}/c$, $\mathbf{j}_{e,i}$ are the electron/ion currents in the laboratory frame of reference and $\mathbf{v}_{e}$ and $\mathbf{v}_{i}$ are the electron and ion bulk flow velocities) are the same: Assuming quasi-neutrality ($n_{e} \approx n_{i}$), one gets $\mathbf{j}\cdot\mathbf{E}_{e}^{\prime}\approx\mathbf{j}\cdot \mathbf{E}_{i}^{\prime}$. The latter equality has also been confirmed by the MMS observations (Yao et al. 2017). A way to resolve this problem has recently been proposed by Sitnov et al. (2018) using the double contraction of deviatoric pressure tensor and traceless strain-rate tensor also known as the $Pi\mbox{-}D^{(\alpha)}$ parameter ($\alpha =e,i$) in kinetic plasma turbulence studies (Yang et al. 2017). It is found that the dissipation processes in the tail start around DFs before the magnetic topology change and that they may include the ion dissipation, in agreement with the original concept of the ion tearing instability (Schindler 1974).

*Key points:*

(1) Spontaneous magnetotail reconnection may start in CSs with magnetized electrons and the thickness greater than the thermal ion gyroradius in the regions with the tailward gradient of the equatorial magnetic field $B_{z}$. (2) It starts from spontaneous generation of earthward plasma flows followed by the formation of DFs. (3) New X-lines are formed in this regime as a result of the flux starvation process behind DFs. (4) The IDMR instability has formal properties of the long-sought ion tearing instability (Schindler 1974), although in its initial phase no field line tearing takes place, and it resembles instead a slingshot process.

*Open questions:*

Which factors determine the domination of the MFRI over the B/I instability?

How does the MFRI growth in the IDMR regime depend on the CS thickness and the lobe plasma pressure?

How can the ion and electron Landau dissipation processes for the IDMR regime be quantified?

What are the tearing stability properties of weakly anisotropic embedded TCSs?

### MHD and Kinetic Stability of MFRI-Prone States

**Fig. 22:**
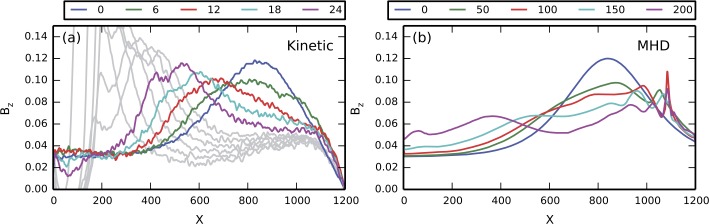
$B_{z}$ dependence on the X-coordinate in the tailward direction as a function of time in (**a**) the PIC simulations by Sitnov et al. (2013) and (**b**) the MHD simulations by Merkin et al. (2015). The time in the simulation normalized units is indicated at the top. The gray contours in panel (**a**) depict an explosive $B_{z}$ growth phase not observed in the MHD simulations. The figure is reproduced from Merkin et al. (2015)

Based on the energy principle of tearing-type (2-D) perturbations of a generalized 2-D current sheet, Merkin et al. (2015) suggested that the kinetic IDMR regime may have an ideal MHD analogue or phase, with the condition that the stability parameter $C_{d}^{2}>\gamma$ as opposed to $C_{d}^{2}>1$ in the kinetic case, where $\gamma$ is the polytropic index. Indeed, Pritchett (2015) showed that in their 2.5-D PIC simulations the kinetic instability did initiate with an ideal-like phase whereby the initial $B_{z}$ hump shifts earthward. This behavior was qualitatively similar to the initial phase of the evolution of similar magnetotail equilibria (with a tailward $B_{z}$ gradient) in PIC simulations with open boundary conditions on the earthward side of the simulation box (Sitnov et al. 2013, 2014; Bessho and Bhattacharjee 2014). In order to explore whether this behavior had an analogue in an ideal MHD system, Merkin et al. (2015) performed 2-D MHD simulations of the same equilibria as Sitnov et al. (2013) using a finite box modification of the LFM MHD code (Lyon et al. 2004). Since this evolution involved formation of earthward plasma flows, the MHD simulations were carried out with open earthward boundary conditions and did reveal a similar behavior (Fig. 22) manifested in the earthward displacement of the initial $B_{z}$ accumulation region. Note here that the formation of persistent ridge-like enhanced $B_{z}$ structures in the tail has been confirmed by global MHD simulations of substorm growth phase (Sect. 2.4) and steady magnetospheric convection (Garcia-Sage et al. 2016). Moreover, the latter simulations showed signatures of the MFRI in regions where the $C_{d}$ exceeded its MHD stability threshold.

More recently, Birn et al. (2018) performed similar MHD simulations with closed boundaries consistent with the energy principle. These simulations confirmed the previous results obtained in MHD and PIC simulations with open boundaries (Merkin et al. 2015; Sitnov et al. 2013, 2014; Bessho and Bhattacharjee 2014; Sitnov et al. 2017) as well as PIC simulations with closed boundaries Pritchett (2015), and demonstrated apparently unstable behavior of equilibria with a tailward $B_{z}$ accumulation followed by explosive $B_{z}$ growth and the formation of a DF.

While the numerical simulations demonstrated unstable behavior in specific equilibria, the parameter space of the instability is large and remains unexplored. To lay the groundwork for more thorough investigation of equilibria with different parameters, Merkin and Sitnov (2016) carried out an analytical examination of the equilibria described by (4) and explored their stability properties with respect to various parameters defining the equilibrium. In addition, since a tailward $B_{z}$ gradient may result in an inverse (earthward) gradient of the flux-tube entropy, these equilibria may potentially be ballooning/interchange unstable as well (e.g., Pritchett and Coroniti 2010, 2013). Therefore, MFRI and interchange stability of magnetotail equilibria need to be considered in concert. Moreover, while the presence of a background plasma pressure—resulting, for instance, from the current sheet embedded in a thicker plasma sheet (Artemyev et al. 2010)—is known to be stabilizing for interchange (Schindler and Birn 2004), similar stabilization of MFRI, at least in MHD, has only recently been revealed in simulations by Birn et al. (2018). Here we summarize briefly the results by Merkin and Sitnov (2016) and expand them to include the background stabilization effect for MFRI.

MHD interchange stability condition for asymptotic 2-D magnetotail equilibria can be written as

5 $$ \gamma(1+p)>2/Q, $$

where $p=P_{b}/P_{\mathrm{cs}}$, $P_{b}$ is the background plasma pressure, $P_{\mathrm{cs}}$ is the current sheet pressure, and $Q = V^{-1}\partial_{\varPsi}V$ is the logarithmic derivative of the flux tube volume, $V$, with respect to the vector potential, $\varPsi$. This condition is equivalent for these equilibria to the classical interchange stability condition $\delta(PV^{\gamma})>0$ (Bernstein et al. 1958). At the same time, the MHD stability criterion for MFRI can be written as

6 $$ \gamma(1+p)>C_{d}^{2}, $$

adding the new effect of the background pressure on the left-hand side to the initial result by Merkin and Sitnov (2016, note also the power of 2 on the right-hand side which was inadvertently omitted in the original work due to a typo). From the similarity of the above equations and the fact that the parameter $Q$ in (5) is determined by an integral similar to $C_{d}$ (the flux tube volume $V$), it is clear that the MFRI and interchange stability properties of these equilibria are intimately related.

**Fig. 23:**
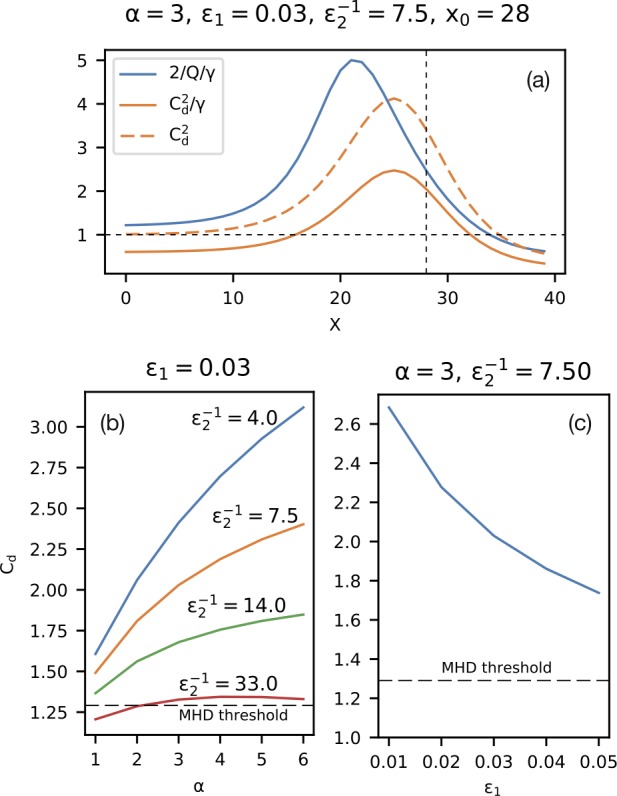
(**a**) Interchange (blue) and MFRI (orange) stability parameters (see Eqs. 5 and 6) as functions of distance along the tail, $x$. The horizontal dashed line indicates the MFRI stability threshold, while the vertical dashed line marks the location of the $B_{z}$ peak. The specific equilibrium parameters are indicated at the top. (**b**) The dependence of the $C_{d}$ parameter on the $B_{z}$ hump amplitude, $\alpha$ for the different hump sizes, $\varepsilon_{2}^{-1}$, and $\varepsilon_{1}$ value indicated at the top. The MHD stability threshold ($C_{d}^{2}= \gamma$) is shown with the dashed horizontal line. (**c**) $C_{d}$ dependence on the level of tail stretching $\varepsilon_{1}$ given the other parameters indicated at the top

The results of the work by Merkin and Sitnov (2016) are rearranged and summarized in Fig. 23. Figure 23a shows the dependence of $C_{d}^{2}/\gamma$ (orange, solid) and $2/(\gamma Q)$ (blue) on the $x$-coordinate for the equilibrium of type given by (4) with the specific parameters indicated at the top. The horizontal dashed line indicates the MHD threshold for the MFRI instability ($C_{d}^{2}/\gamma=1$) given by (6) in the absence of the background, which coincides here with the interchange stability threshold ($2/(\gamma Q)=1$) following from (5). The vertical dashed line indicates the location of the $B_{z}$ peak. The plot shows that both interchange and MFRI are most unstable earthward of the $B_{z}$ peak, in the region of the strong tailward $B_{z}$ gradient. It also demonstrates that for the specific equilibrium parameters, MFRI gets stabilized by background pressure before interchange ($2/Q>C_{d}^{2}$ everywhere).

The dashed orange line in Fig. 23a shows the destabilization parameter $C_{d}^{2}$ without dividing by $\gamma$, suggesting that interchange-stable regions around the $B_{z}$ hump may still be unstable with respect to the kinetic MFRI, essentially, the ion tearing (Sitnov and Schindler 2010). This is consistent with the results of 3-D PIC simulations of the MFRI (Sitnov et al. 2014), discussed in Sect. 3.3. In particular, Fig. 20 shows that while interchange motions substantially modulate the 2-D DF, its formation is the dominant process in the 3-D dipolarization picture.

Figures 23b–c concentrate specifically on MFRI properties in the absence of background and summarize the results of a numerical calculation of the $C_{d}$ parameter for the various combinations of the three parameters $\{\alpha, \varepsilon_{1}, \varepsilon_{2}\}$. Figure 23b shows the dependence of $C_{d}$ on $\alpha$ for a fixed amount of tail stretching ($\varepsilon_{1}$) and different scale sizes of the $B_{z}$ gradient ($\varepsilon_{2}^{-1}$). Thus, the theory predicts the following relationships: i) Potential for the MFRI increases significantly with $\alpha$ unless the $B_{z}$ hump is very wide; ii) Potential for kinetic MFRI exists for all $\alpha\ge1$, while MHD instability threshold is reached for most but not all conditions and requires sufficiently narrow humps and sufficiently large values of $\alpha$. Figure 23c additionally explores the dependence of $C_{d}$ on the amount of tail stretching. It demonstrates that the potential for MFRI reduces significantly for less stretched tails but that, for the specific parameters chosen, even the least stretched tail configuration may still be unstable.

*Key points:*

(1) Magnetic flux release (MFR) or “hump” instability has been demonstrated in ideal MHD simulations of magnetotail equilibria with a tailward $B_{z}$ gradient. Analogous kinetic simulations indicate a similar regime where the same equilibria develop initially without a change in topology. (2) Tailward $B_{z}$ gradients may also be interchange unstable. Stability criteria for MFRI and interchange are similar, with background plasma pressure playing a stabilizing role. (3) Parameter space of the instability is large; only limited analytical exploration has been performed to date.

*Open questions:*

What are the distinctive features of the ideal-MHD MFRI in 3D? In particular, is it possible to form a 2D MFRI-unstable $B_{z}$ hump if it is also interchange unstable?

Tailward $B_{z}$ gradients form also in localized, but stable, TCSs from isentropic deformation in which the field line entropy remains monotonic. What are the exact conditions that render hump configurations MHD unstable?

### Ballooning/Interchange Instability

It is by no means clear that a 2-D treatment is sufficient to explain the origin of the disruptions that occur in the magnetotail during a substorm. In fact, there is clear observational evidence that these disruptions are confined at least initially to regions of a few $\mbox{R}_{E}$ extent in the east-west direction (Sergeev et al. 1996; Angelopoulos et al. 1997; Lui et al. 1998; Petrukovich et al. 1998; Nakamura et al. 2004). The corresponding current-aligned instabilities (see Lui 2004 for a review) were extensively discussed as a mechanism of magnetotail explosions within the current disruption scenario of substorms (Lui et al. 1991). The possible role that might be played by various 3-D current-aligned instabilities in providing the plasma nonideality necessary for reconnection in collisionless space plasmas has been reviewed by Büchner and Daughton (2007). Current-aligned instabilities have also been described as a part of the 3-D IDMR picture (Sitnov et al. 2014) in the study of Lui (2016). Here we consider the possible role that could be played by variants of the ballooning and interchange modes (Rosenbluth and Longmire 1957).

The MHD ballooning mode has been suggested to occur as a consequence of the strong field line curvature in the center of the plasma sheet (Roux et al. 1991; Bhattacharjee et al. 1998; Miura 2001; Cheng and Zaharia 2004). Schindler and Birn (2004) performed an MHD stability analysis of 2-D magnetotail equilibria (with no cross-tail $B_{y}$ component) under general 3-D perturbations. This case can be reduced to analyzing stability with respect to ballooning modes alone. They found a general result that stability/instability was related to a general interchange criterion based on entropy: stability (instability) occurs for a tailward increase (decrease) of the entropy $S = \ln(pW^{\gamma})$, where the flux tube volume $W = \int ds/B$ and $\gamma$ is the adiabatic gas constant. They concluded that most configurations with realistic tailward pressure profiles are stable. The unstable cases were limited to extremely rapid tailward pressure decay, and even for these unstable cases, a small background pressure component could typically remove the instability. These results were limited to the case of strongly stretched configurations, and thus do not exclude the possibility of a ballooning mode occurring in the transition region from the dipole to tail field (Roux et al. 1991; Cheng and Zaharia 2004).

**Fig. 24:**
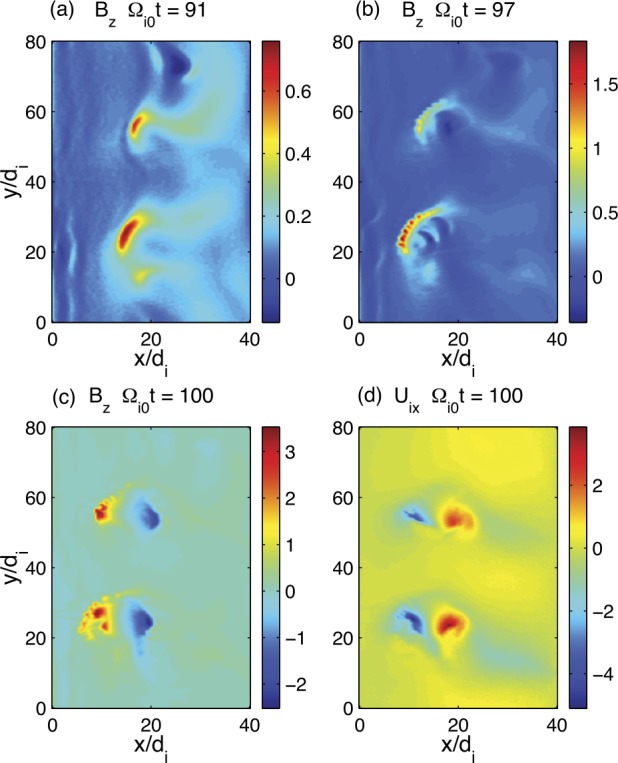
Structure in the equatorial plane of the magnetic field $B_{z}$ as observed at various times in a 3D PIC simulation of a hump configuration: (**a**) $\varOmega_{i0}t = 91$, (**b**) $\varOmega_{i0}t = 97$, and (**c**) $\varOmega_{i0}t = 100$; (**d**) structure in the equatorial plane of the ion flow velocity $U_{ix}/V_{Ti}$ at $\varOmega_{i0}t = 100$. Adapted from Pritchett (2013)

Consideration of the pressure-balance catastrophe associated with steady convection in the tail (Erickson and Wolf 1980) has led to the suggestion that a deep minimum in the equatorial $B_{z}$ could form in the inner plasma sheet (Erickson 1984; Hau et al. 1989; Hau 1991; Erickson 1992). Such midtail minima have been observed during periods of extended magnetospheric convection (Sergeev et al. 1994), and Saito et al. (2010) presented evidence of local magnetic field minimum formation in the equatorial region near $11\,\mbox{R}_{E}$ at the end of a substorm growth phase. In addition, the presence of increasing $B_{z}$ profiles has been deduced from statistical studies as occurring in the mid-tail prior to substorm onset (Machida et al. 2009). Such considerations have led to a number of investigations of possible ballooning/interchange (B/I) modes occurring in kinetic plasmas. Pritchett and Coroniti (2010, hereafter PC2010) and Pritchett and Coroniti (2011, 2013) demonstrated the existence of a kinetic instability occurring in the presence of a tailward $B_{z}$ increase (or more generally a decreasing entropy profile). This mode was identified as the low-frequency extension in a curved magnetic geometry of the lower-hybrid-drift instability in straight magnetic fields. Unlike the case of interchange modes in MHD, there is a substantial perturbation resulting from the finite $E_{||}$ field that results in strong field-aligned electron flows. In its nonlinear evolution, the mode develops interchange heads that extend into the near-Earth dipole regions. In some circumstances, the $B_{z}$ field can be driven southward in the wake of the heads, resulting in the onset of localized magnetic reconnection and a violent disruption of the plasma sheet. An example of this process (Pritchett 2013) is shown in Fig. 24. The $B_{z}$ hump maximum is located initially at $x/d_{i} = 32$. By $\varOmega_{i0}t = 91$ (panel a), two clearly defined B/I heads have emerged from the hump region and are propagating to the left. By $\varOmega_{i0}t = 97$ (panel b), these heads are beginning to filament into smaller units $\sim d_{i}$ in size, and the $B_{z}$ behind the heads has become negative. In panel (c) the bursts of reconnected magnetic field with strength on the order of $2\mbox{--}3B_{0}$ and cross-tail size $\sim5\mbox{--}10d_{i}$ are apparent. Panel (d) shows the ion flow velocity $U_{ix}$ for the same time as in panel (c). The flow is dominated by high-speed exhausts of magnitude $2\mbox{--}4V_{Ti}$ in both directions away from the localized reconnection regions. Some support for this connection between B/I oscillations and reconnection is provided by three THEMIS events (Uritsky et al. 2009; Sergeev et al. 2012b; Panov et al. 2012a) where B/I signatures were identified. Although reconnection was observed in all three events, in the 28 February 2008 event the oscillations persisted over tens of ion gyroperiods without a substorm onset. Thus, it appears that the B/I process does not always lead immediately to magnetotail reconnection.

**Fig. 25:**
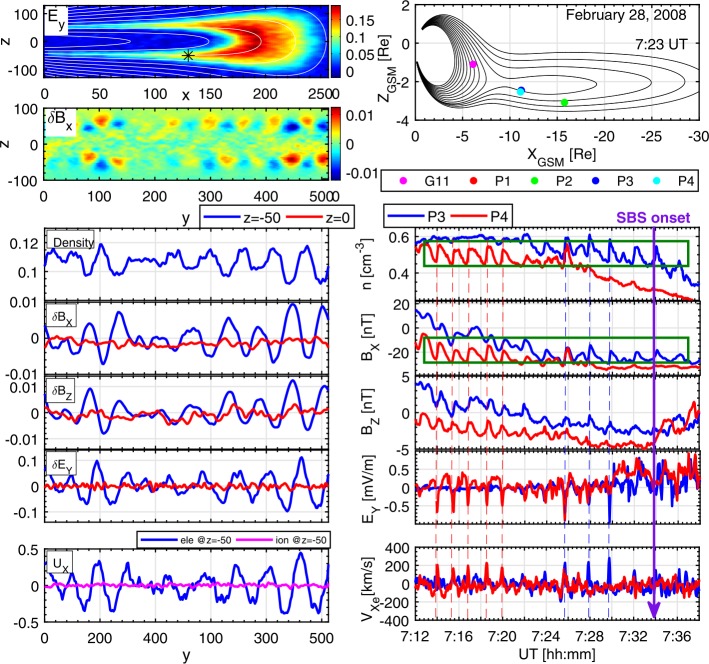
(Left) Results from PC2010 run: (X,Z) cut of the Y-component of the electric field at $\varOmega t=37.5$ at $y=464$. The white lines are magnetic field lines. (Y,Z) cut of the X-component of the magnetic field oscillations, and Y-cuts of the density, the X- and Z-components of the magnetic field oscillations, the Y component of the electric field, the X-component of the ion (magenta) and electron (blue) velocity at $\varOmega t=37.5$, and $x=130$. (Right) Magnetic field lines according to the AM-03 model shown in the noon meridian GSM plane on 28 February 2008 at 7:23 UT (locations of the P1–P4 THEMIS and GOES 11 spacecraft are overplotted, see legend for color coding); data from P3 and P4 on 28 February 2008 between 7:12 and 7:38 UT: (from top to bottom) electron density; $X_{\mathrm{GSM}}$- and $Z_{\mathrm{GSM}}$-component of the magnetic field; $Y_{\mathrm{GSM}}$-component of the electric field; $X_{\mathrm{GSM}}$-component of the electron velocity. P4 data are shifted by −50 seconds. See legends for color coding. Adapted from Panov et al. (2012a), Panov et al. (2012b)

Recent THEMIS (Angelopoulos 2008) observations around $10\mbox{--}12\,\mbox{R}_{E}$ (P3, P4, P5) have made it possible to directly investigate the cross-tail size and propagation velocity of plasma tubes with B/I signatures. The left column of Fig. 25 shows field and plasma parameters from the PC2010 particle-in-cell run. The equatorial field profile ($B_{z}$) was chosen to have a minimum between $x=32$ and 96, and the tailward gradient of $B_{z}$ was initially set up between $x=96\mbox{ and }224$. These results are for the simulation time $\varOmega_{i0} t=37.5$ when the instability was still in the linear stage. The top panel shows the ($x,z$)-cut of the electric field Y-component at $y=464$. The white lines are magnetic field lines. This electric field structure makes clear that the electron flow in the simulation is almost entirely field aligned, demonstrating that the B/I mode is a non-local mode in which significant kinetic ion and electron effects (bounce and drift resonant interactions) are present which are not included in an MHD treatment. The next panel shows an ($y,z$)-cut of perturbations produced by B/I in the $B_{x}$ magnetic field component at $x=130$ (slightly tailward of $B_{z}$ minimum, as marked by the star showing the location of a virtual spacecraft at $x=130$, $z=-50$ in the top panel). The perturbations are absent across the neutral sheet, so the mode at this cross-section is mostly confined to the off-equatorial part of the plasma sheet. The peaks above and below the neutral sheet either both increase or both decrease the field, revealing a sausage-like finger structure produced by the kinetic B/I. The perturbations produced by the B/I in $B_{x}$ and the other fields drift duskward at about one tenth of the ion thermal speed. Due to this drift, the Y-profile of perturbations is nearly equivalent to a temporal plot of parameters that would be observed by a magnetospheric spacecraft.

The next four panels in the left column of Fig. 25 show Y-cuts of the basic parameters, suggesting duskward propagation of the entire pattern (as observed in simulations); these plots can be directly compared with temporal variations observed in the magnetotail. Note that since at $z=0$ the density is constant on the scale of the B/I wavelength and is also much larger than at $z=-50$, we do not show it in this figure. The oscillations in the magnetic field components $\delta B_{x}$ and $\delta B_{z}$ are in phase; those in the electric field $E_{y}$-component are phase shifted by $\pi/2$. The $E_{y}$ oscillations are, however, in phase with the $X$-component of the electron velocity (bottom panel), which is the largest of the three $\mathbf{U_{e}}$ components and not accompanied by comparable ion velocity variation. Strong $\delta B_{x}$ (compressional) and $\delta N_{e}$, together with phase-shifted $\delta E_{y}$ and $\delta U_{xe}$, reveal distinctive B/I signatures in the cross-section near the $B_{z}$ minimum, and the structure of B/I fingers cross-tail-drifting in the westward direction.

The right column of Fig. 25 presents a long oscillation event on 28 February 2008 between 7:12 and 7:38 UT (the oscillations started at about 7:00 UT, not shown here). The top panel shows the magnetic field lines according to the AM-03 model in the noon meridian GSM plane on 28 February 2008 at 7:23 UT (locations of the P1–P4 THEMIS and GOES 11 spacecraft are overplotted, see legend for color coding). In the following panels data from THEMIS probes P3 and P4 are plotted next to the corresponding parameters from the PC2010 run in the left column of Fig. 25. The observations at P4 (red line) were shifted in time by 50 seconds to highlight the similarity of curves at P3 and P4. In this event, a substorm onset was identified at about 07:34 UT as onset of Pi2 waves and current wedge formation on the ground, together with strong oscillations at GOES-11 (at $\sim22.5~\mbox{h}$ MLT).

Anti-correlated oscillations of the electron density and the $X_{\mathrm{GSM}}$ magnetic field component with a period of $\sim100$ seconds were observed by P3 and P4. Several similarities with B/l signatures can be seen by comparing the left and the right columns in Fig. 25. Smaller-amplitude oscillations were observed in $B_{z}$ and large oscillations in $E_{y}$ in comparison to those in $B_{x}$ and $E_{y}$. Oscillations in the $X_{\mathrm{GSM}}$-component of the electron velocity were observed, without comparable ion velocity oscillations (for better visibility we do not show the ion velocity). The electron velocity oscillations along the X-axis are entirely field aligned. The electron velocity components have been time averaged over 5 probe spins (15 seconds) to remove high-frequency thermal noise. More detailed analysis (cf. Fig. 14 in Panov et al. 2012a) has reveled that the current sheet oscillations are sausage-like (i.e., balloons) rather than flap-like structures (i.e., kinks). The oscillations in the perpendicular $Y_{\mathrm{GSM}}$-(not shown) and $Z_{\mathrm{GSM}}$ magnetic field components are one order of magnitude smaller than in the field-aligned $X_{\mathrm{GSM}}$ magnetic field component. The $B_{x}$-oscillations were accompanied by phase-shifted electric field oscillations with the major $E_{y}$-component, such that $E_{y} \sim-\partial B_{x}/ \partial t$ (not shown here).

The long duration of the oscillations and different locations of P3 and P4 with respect to the neutral sheet allowed us to see that spiky $E_{y}$, $V_{x}$ and density oscillations were substantially weaker both near the neutral sheet (large density $\sim0.6\text{ cm}^{-3}$ and small $|B_{x}|$ amplitude) and in the lobes (density was below $0.4~\text{cm}^{-3}$ and $|B_{x}|$ exceeded 30 nT). We indicated the region of largest oscillation amplitudes between the plasma sheet center and its outer edge with green rectangles in Fig. 25. P3 and P4 were separated by 5950 km mostly along the $Y_{\mathrm{GSM}}$-axis. Long-lasting oscillations allowed us to compute the cross-correlation of the signals from P3 and P4, which gave a distinct peak at 50 seconds and indicated duskward propagation at a velocity of about 120 km/s. The characteristic cross-tail scale for the half-period of $T=50~\mbox{seconds}$ is then 6000 km.

There is a disagreement between the simulations and the THEMIS observations for the oscillation amplitude. Whereas in THEMIS observations $\delta B_{x}$ was very strong between the neutral sheet and the lobes (up to 50% of the lobe field), it did not exceed 5% of the lobe field in the PC2010 run. The amplitude of $\delta B_{x}$ may, however, depend on other parameters, such as the plasma beta and ion-to-electron mass ratio. Despite the disagreements, further evidences of the B/I instability development were recently found in the transition region between the dipole and tail fields. Panov and Pritchett (2018b) reported that B/I heads were observed at the tailward side of a $B_{z}$-dip with $\partial\mbox{B}_{z}/\partial \mbox{x}\approx {-}10~\mbox{nT}/\mbox{R}_{E}$, where they appeared to drift azimuthally toward dawn, in accord with PIC simulations of a charged current sheet. The presence of electromagnetic ion-cyclotron wave activity with $\delta E_{y}/\delta B_{z}\approx 4.5\,\mbox{V}_{A}$ in a region of the strongest differential drift between the electrons and ions appeared to ripple the background B/I head shape (Panov and Pritchett 2018a).

*Key points:*

Kinetic B/I instabilities may be an alternative mechanism for the onset of explosive magnetotail activity. In its nonlinear evolution, such an instability develops interchange heads that extend into the near-Earth dipole region; in some cases the wakes behind these heads can be the site of a violent disruption of the plasma sheet. These instabilities can occur in both neutral (where the accompanying waves propagate duskward) and charged (waves propagate dawnward) current sheets.

*Open questions:*

How can B/I unstable configurations evolve from stable configurations? Does this necessarily involve violation of entropy conservation? What are the possible mechanisms and what are their characteristic properties and time scales?

Are the properties of the B/I waves different in negatively charged as opposed to neutral thin current sheets? How do these properties depend on the thickness of the TCS?

What are the auroral signatures of B/I instabilities in the pre-onset magnetotail and after the onset? In particular, do B/I waves reflect special features of the pre-onset metastable current sheet, such as the tailward $B_{z}$ gradient, or do they reflect the mechanism of the onset and determine its strength?

Can B/I be the primary trigger of DFs or substorm onsets and how does it interact with reconnection motions in EDMR and IDMR regimes?

What are the relative roles of B/I and 3-D current aligned instabilities in the onset mechanism of the tail current sheet explosions?

### Flapping Motions

Flapping motions (FMs), in spite of their abundance in the tail (e.g. Sergeev et al. 2004, 2006), represent one of the most mysterious modes of the magnetotail activity. Their driving mechanisms, relation to the main modes of activity, such as substorms and BBFs, as well as other plasma instabilities remain insufficiently investigated. Perhaps the only fact that links FM to the tail activity is their correlation with fast flows (e.g. Sergeev et al. 2006). At the same time, FMs are very prominent, and their large amplitudes offer an important opportunity to probe the current sheet structure (Runov et al. 2005; Petrukovich et al. 2011) (see Sect. 2.1 for more detail).

In the limit of 1D equilibria with $B_{z}=0$ flapping represent kink waves whose mechanisms might be drift kink (Daughton 1999) and ion kink instabilities (Karimabadi et al. 2003; Sitnov et al. 2004). However, in 2D current sheets with electrons magnetized by the finite $B_{z}$ field the instability mechanisms are less obvious, and some 3D PIC simulations (e.g. Pritchett 2013; Pritchett and Coroniti 2013) reveal no flapping, which is seen, in particular, from symmetric profiles of the dawn-dusk electric field $E_{y}(z)$ across the current sheet (e.g. Pritchett and Coroniti 2013, Fig. 4). A mechanism of the flapping generation coined the double gradient instability was proposed by Erkaev et al. (e.g. 2007). Using a reduced MHD description of flapping modes they obtained the following expression for their complex frequency

7 $$ \omega_{f}=\bigl((\partial B_{x} /\partial z) ( \partial B_{z} /\partial x)/(4 \pi\rho)\bigr)^{1/2}, $$

where $\rho$ is the plasma mass density. Since in the tail $\partial B_{x} /\partial z >0$ the frequency $\omega_{f}$ becomes an imaginary number for $\partial B_{z} /\partial x <0$, suggesting an exponential growth of FMs in that region. Thus, similar to IDMR and B/I instabilities, flapping motions are favored by the same tail feature, namely the tailward gradient of the equatorial $B_{z}$ field. However, it remains unclear if the condition $\partial B_{z} /\partial x <0$ is both sufficient and necessary for FM destabilization because the theoretical analysis (Erkaev et al. 2007) contains some ad hoc assumptions.

**Fig. 26:**
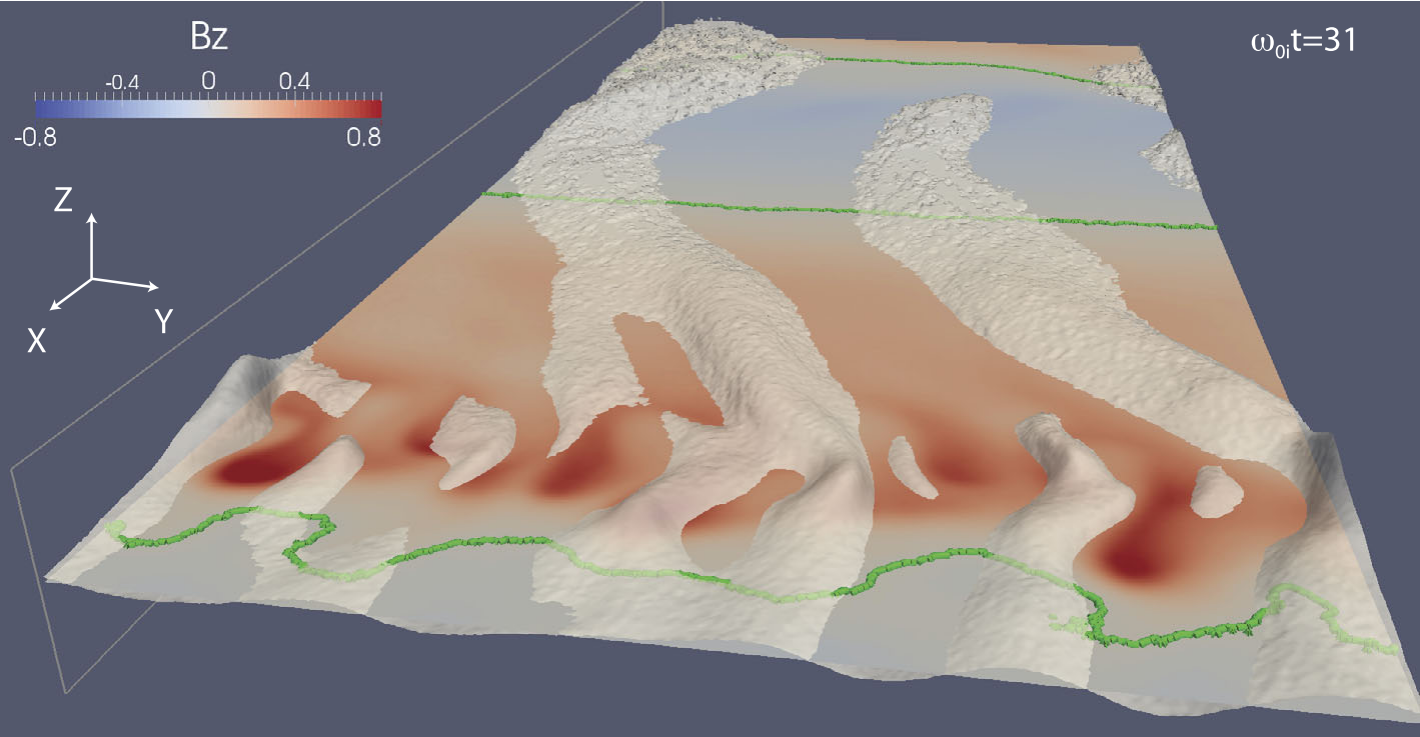
Perturbations of the DF and the new X-line in its trail (central green line) caused by flapping motions in 3D PIC simulations of IDMR (Sitnov et al. 2014). Flapping is shown by the corrugated translucent $B_{x}=0$ surface. The neutral plane ($z=0$) is color-coded by $B_{z}$ to indicate the concurrent reconnection and B/I motions. Bottom and top green traces within the plane depict the new O-lines ahead of and behind the DF. The latter supersedes the initial equilibrium X-line, which was used in simulations to mimic open tailward boundary conditions. The dimensions of the displayed subset of the simulation box are indicated with the rectangles: vertical $x=(-22.5d _{i},0)$, $y=0$, $z=(-d_{i}, d_{i})$ and horizontal $x=(-22.5d_{i},0)$, $y=(0,10d_{i})$, and $z=0$

Excitation of the flapping waves in the tail equilibria with a tailward $B_{z}$ gradient was demonstrated in MHD (Korovinskiy et al. 2013) and PIC simulations with open boundaries (Sitnov et al. 2014). In particular, Fig. 26 shows flapping in the tail CS in the form of the corrugated translucent $B_{x}=0$ surface together with MFRI and B/I motions reflected by the color-coded $B_{z}$ profile, as well as X- and O-lines.

**Fig. 27:**
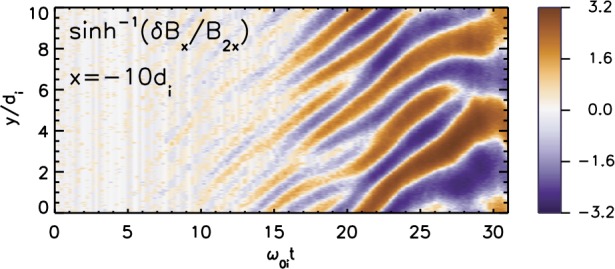
Stacked time plots of flapping waves in 3D PIC simulations (adapted from Sitnov et al. 2014). Variations of the magnetic field are defined as $\delta B_{x}=B_{x}-\langle B_{x} \rangle_{y}$, where the $\langle... \rangle_{y}$ average is taken over the Y coordinate at the fixed distance $x=-10d_{i}$. To mitigate the strong increase of the magnetic field perturbation the color-coded parameter is shown as a inversed hyperbolic sine function with $B_{2x}=0.01B_{0}$. The phase speed of buoyancy and flapping waves (in units of Alfvén speed $v_{A}$) is reflected by the slope of the stripes

A distinctive feature of flapping motions is that they usually propagate from the midnight meridian toward the flanks of the tail CS (e.g. Sergeev et al. 2004). An explanation of this feature may be the finding that in 2D Harris-type equilibria with the thickness $L_{z} \sim\rho_{0i}$ and $v_{Di} \sim v_{A}$ (Sitnov et al. 2014) and the bulk flow velocity dominated by ions $v_{Di}=-v_{De}=V_{Ti} \rho_{0i}/L_{z}$ the phase velocity of FMs is close to the Alfvén speed (Fig. 27). Thus, FM waves are essentially motionless ($|v_{y}| \ll v_{A}$) in the system of reference moving with the bulk ion flow. Then their flankward motion can be explained by the corresponding plasma motions on the MHD scale (e.g. Merkin and Lyon 2010). The latter are dominated by ions, whereas the Harris-type TCS are dominated by electrons due to their negative charging (for detail see, for example, Nishimura et al. 2016; Lu et al. 2016, and refs. therein).

*Key points:*

(1) FM correlate with fast flows. (2) they may appear as an instability in magnetotail configurations with the tailward gradient of the equatorial magnetic field $B_{z}$.

*Open questions:*

What features, other than the correlation with fast flows, link FM with the explosive tail activity?

What is the contribution of FM to the tail energy budget?

How do FM depend of the value of the equatorial magnetic field $B_{z}$ and its distribution along the tail?

### Auroral Breakup and Ionospheric Signatures

The spatio-temporal displays of the aurora provide a valuable tool for visualizing the structure and dynamics of explosive processes in the magnetotail. In particular, for the near Earth tail, it is a reasonable assumption that tail processes map along field lines almost instantaneously to the ionosphere. Recent work by Ferdousi and Raeder (2016) has shown that signals from the mid tail travel to the ionosphere in as little as 60 seconds. Since the magnetic configuration does not change much during such periods, the ionosphere can act like a projection screen for magnetotail processes. Various processes can be invoked to create the auroral emissions, such as scattered particles, or field-aligned currents (FACs).

As an example, auroral streamers (north-south aligned auroral arcs), which are usually considered as a visible ionospheric manifestation of earthward flow bursts associated with magnetotail reconnection, are often observed as precursor activity prior to the initial brightening arc and its subsequent auroral breakup. Detailed magnetic mapping indicates that the streamers do not map to the flow channels themselves but rather to upward field-aligned currents generated by shear at the duskward edges of the flows (Nakamura et al. 2001). Kepko et al. (2009) presented multi-spectral ASI observations of an onset event preceded by an equatorward-moving patch that coincided with an earthward flow burst in the near-Earth tail. They concluded that the auroral breakup likely results from the arrival of the flow burst. Nishimura et al. (2010) expanded on that idea and claimed that most auroral breakups are preceded by the arrival of streamers originating from the poleward auroral boundary, or vice versa, that auroral breakups are generally caused by streamers. Even though the streamer is usually observed prior to a substorm expansion onset (Nishimura et al. 2011), it does not mean that the streamer triggers the onset. Besides, there are also many substorm onsets not preceded by an auroral streamer. Mende et al. (2011), Shi and Zesta (2014), Murphy et al. (2014). Recently, Miyashita and Ieda (2018) revisited three substorm events reported by Nishimura et al. (2010, 2011), particularly with a focus of the arrival timing of the preonset aurora relative to the thee steps of auroral onset arc development, initial brightening, wave-like structure, and poleward expansion. Their detailed timing analysis indicated that the preonset auroral streamers reached the auroral onset arc but away from the initial brightening site for two events, while the preonset aurora did not reach the initial brightening site. The results allowed them to conclude that auroral streamers and related processes are not responsible at least for the initial brightening of onset arc. Thus, it still remains controversial which role, if any, auroral streamers play in substorm onsets. As noted by Mende et al. (2011), space based auroral imagers were not sensitive enough to reliably detect the streamers, and ground based imaging with ASI, while sensitive enough, lacks sufficient coverage to provide good statistics.

**Fig. 28:**
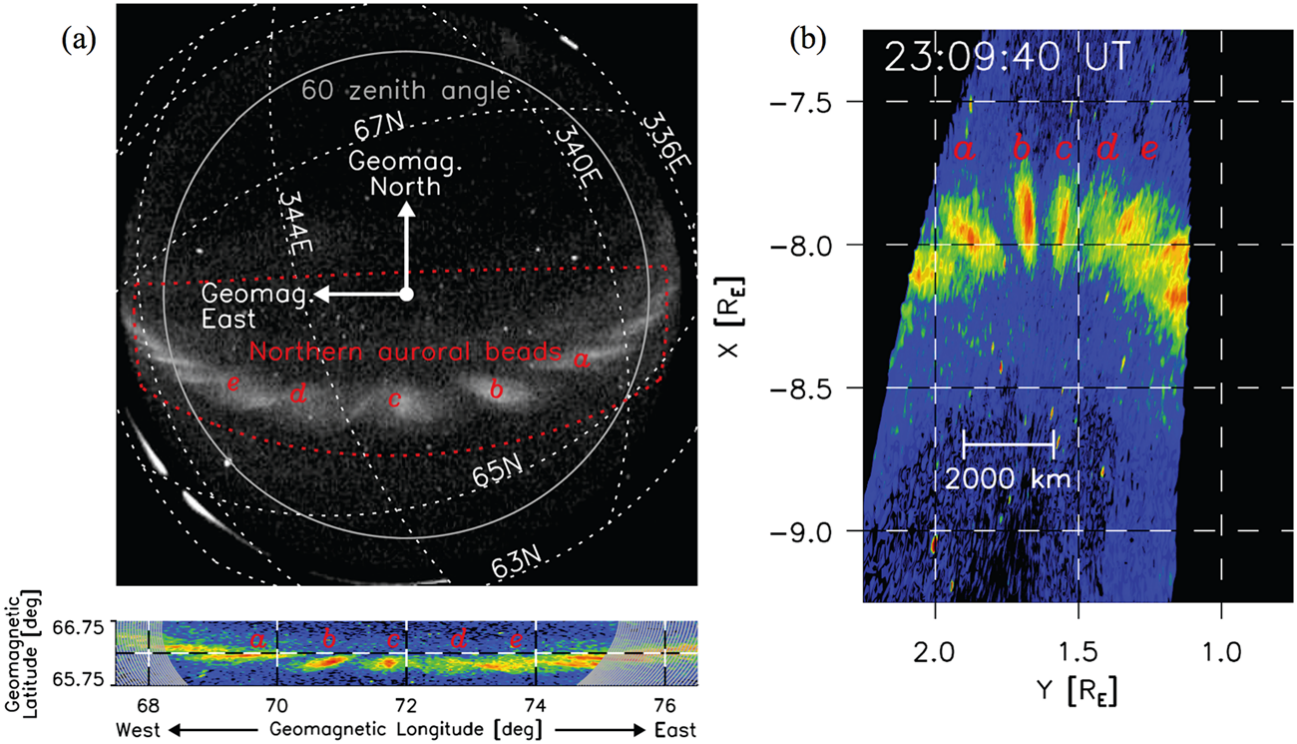
(**a**) An example of conjugate auroral beads in Iceland (adapted from Motoba et al. 2012). (**b**) Mapping of the auroral beads into the equatorial plane with T96 model

Auroral beads/rays are another auroral form which reflects the magnetotail activity. Beads are observed most often along the east-west aligned arc minutes prior to the onset of auroral breakup (Donovan et al. 2006; Liang et al. 2008; Sakaguchi et al. 2009; Motoba et al. 2012; Hosokawa et al. 2013; Kalmoni et al. 2015, 2017; Nishimura et al. 2016). The faint and small-scale bead/ray structures start to grow slowly and spontaneously along the arc, and then evolve more dynamically and non-linearly into brighter and larger-scale spiral structures. The wavy auroral forms have a characteristic wavelength of $\sim10\mbox{--}100~\text{km}$ and propagate westward, eastward, or in both directions at a speed of $1\mbox{--}10~\text{km}/\text{s}$. The beads’ emissions are likely caused by localized filamentary FAC structures. Such FAC structuring could be caused locally at low altitudes just above the aurora, or by processes in the magnetotail. Motoba et al. (2012) demonstrated for the first time using simultaneous observations of northern and southern auroral beads that they evolve synchronously and have remarkable similarities (Fig. 28a). The unique inter-hemispheric similarities strongly suggest that the beads have a common driver in the magnetotail. Assuming that the auroral beads are a simple ionospheric projection of the physical processes occurring in the plasma sheet, the scale size of auroral beads in the ionosphere is found to be typically on the order of the gyroradius ($\sim500\mbox{--}800~\text{km}$) of $1\mbox{--}10~\text{keV}$ protons in the source magnetosphere (Fig. 28b).

This leaves open a number of physical scenarios that could generate beads. The temporal evolution of beads suggest that they are driven by an instability in the tail. Several candidates have been hypothesized, including such instabilities as ballooning-type instability (Kalmoni et al. 2015, 2017; Nishimura et al. 2016), cross-field current instability (Kalmoni et al. 2015; Lui 2016), and inertial Alfvén wave turbulence associated with Kelvin-Helmholtz and tearing instabilities of the thin current sheet (Kataoka et al. 2011). Whereas auroral beads are often considered to be a projection of instabilities generated in the plasma sheet, it may be worthwhile to consider the active role of the ionosphere in the formation of auroral structures, e.g., ionospheric feedback instability in or above the auroral acceleration region (Hosokawa et al. 2013).

Nishimura et al. (2016) conducted a statistical study of beads and their properties. They found them to be commonly related to substorm onsets. Considering possible tail processes, they concluded that the kinetic ballooning modes, when mapped to the ionosphere, provided the best agreement with the observed phase velocity and growth rates of the beads, as opposed to the electromagnetic ion cyclotron instability and the cross-field current instability, which predict larger propagation speed and smaller wave periods.

A global MHD simulation of the March 23, 2007 substorm event (the THEMIS “first light” event (Raeder et al. 2008, 2010)) also shows signatures of MHD ballooning modes, as predicted by Zhu et al. (2009). The finger-like signatures resembling ballooning modes in the simulation (see, for example, Fig. 5 in Raeder et al. 2010) match quite well the dimensions of beads when mapped to the tail. The B/I signatures in the simulation were rather subtle, but proved to be independent of numerical resolution. In particular, the wavelength was found to be of the order of $0.5\,\mbox{R}_{E}$, in line with typical observations. Remarkably, since this is a MHD simulation, there is no intrinsic scale, such as for example the Larmor radius, that would determine the scale of the B/I modes. In a follow-up study, Raeder et al. (2012) showed that the predicted auroral signature of the B/I mode matches the images of the beads very well, resembling the Motoba et al. (2012) observations, although they are from a different time period. It must be noted, however, that in the simulation the B/I and the beads, although related to the substorm, are not the trigger of tail energy unloading, because the B/I occurs some 10 minutes before onset. This is also consistent with some observations. For example, Henderson (2009) presented a case where large-scale auroral spots were clearly visible in IMAGE auroral images, but some 10 minutes before substorm onset, which also occurred at a different location.

The scales of magnetotail structures predicted from recent 3-D kinetic simulations (Sitnov et al. 2014; Pritchett et al. 2014) are also consent with the properties of bead structures. Thus, at present several hypotheses regarding the physical causes of beads remain viable and require further study.

Another feature relevant to the pre-onset activity with a potential to explain the underlying instabilities is the evolution of the polar cap size, the dark area inside the luminous auroral oval, which reflects the magnetic flux in the open field line region (e.g., Craven and Frank 1987; Liou and Sotirelis 2016). It reaches a maximum during the substorm growth phase, consistent with major substorm models. At the same time, the moment of reaching that maximum may precede the substorm onset by 6 (Liou and Sotirelis 2016) to 40 (Shukhtina et al. 2014) minutes, suggesting that in the late growth phase important changes and reconfigurations of the magnetic flux in the closed field line regions take place, in agreement with independent theory and observations discussed in Sect. 2.

The post-onset auroral morphology is very rich, and in fact, it has become the starting point of the auroral substorm concept (Akasofu 1964). It includes brightening of the most equatorward auroral arc, its poleward expansion with the formation of the westward travelling surge, additional equatorward and eastward expansion, and many mesoscale structures, such as torches, omega bands and post-onset streamers (Nakamura et al. 1993; Elphinstone et al. 1996; Henderson 2012). (One should not forget here that bright auroras, formed predominantly by the field-aligned acceleration and being primarily the images of strong upward FACs, show us only some part of the complicated picture of energy transformation in the magnetosphere.) At the same time, the corresponding auroral morphology appears to be too complex, compared to the pre-onset activity, to trace analogy with the specific post-onset dipolarizations and plasma instabilities in the tail. A few exceptions (e.g., Fig. 37) are mentioned though in the next section, where we describe those post-onset processes.

Thus, recent observational and modeling efforts have made great strides toward understanding and more comprehensively describing the pre-onset ionospheric signatures of magnetotail explosions. They can be summarized as follows.

*Key points:*

(1) Auroral streamers are often (but not always) observed as precursor activity prior to the initial brightening arc; (2) auroral beads/rays emerge along the arc near the auroral breakup region immediately prior to auroral substorm expansion phase onset; (3) both auroral streamers and beads/rays are believed to be ionospheric manifestations of magnetotail explosions, i.e., bursty bulk flow and plasma instability; and (4) the observed characteristic properties (spatial scale, phase velocity and growth rate) of auroral beads/rays qualitatively match those of magnetotail instabilities predicted from high-resolution 3-D MHD and kinetic simulations.

At the same time, a large number of issues remain unresolved. Specifically, a great uncertainty of field line mapping from the nightside ionosphere to the magnetotail leads to major difficulties in understanding the linkages between auroral ionospheric signatures and magnetospheric processes.

*Open questions:*

What drives auroral beads/rays and what controls their evolution?

Where do quiet arcs or growth phase arcs originate in the magnetosphere?

What role do auroral streamers play in the onset process of auroral substorm?

What are the relative roles of processes in the acceleration region and magnetotail plasma sheet for the formation of preonset auroral structures?

What effects does the ionosphere have on the magnetotail configuration and/or dynamics?

## Explosive Magnetotail Dynamics

### Observations of Earthward Transients

Decades of in-situ observations in the magnetotail with increasing temporal resolution of field and plasma measurements showed that post-onset activity in the plasma sheet is highly time-variable and organized in a series of transients with characteristic time scales ranging from a second to hundreds of seconds (see Sharma et al. 2008, for a past review). Spatial scales of the transients estimated from timing of signatures and observed bulk flow velocity or inferred from multi-point measurements vary from a few ion gyroradii to several $\mbox{R}_{E}$.

This activity has been explored under different names and with different definition details. The most widely-used term, the bursty bulk flows (BBFs, Angelopoulos et al. 1992), emphasized a high bulk plasma peak velocity of $\sim1\,V_{A}$, where $V_{A}=B_{L}(\mu_{0} n m_{i})^{-1/2}$ is the Alfvén velocity calculated using the lobe magnetic field $B_{L}$ and plasma sheet density $n$. Its characteristic value in the magnetotail at $R\sim 20\,\mbox{R}_{E}$ is about $10^{3}~\mbox{km}/\mbox{s}$. The total duration of concurrent high-speed flows in BBFs varies from about a minute to $\sim 10\mbox{--}20~\mbox{minutes}$. Typically, a BBF consists of smaller-scale flow bursts (defined by $V>400~\mbox{km}/\mbox{s}$) with a time scale of several tens to about a hundred seconds (e.g., Baumjohann et al. 1990), which are accompanied by peaks of the northward magnetic field component $B_{z}$ and the resulting bursty enhancement of the magnetic flux transport, which can be quantified by the dawn-dusk electric field $E_{y}$.

The rationale for other plasma sheet transient definitions was largely dictated by the specific data analysis goals and data processing methods. One of the first terms emphasizing the transfer of flux, the Nightside Flux Transfer Events (NFTE) was introduced by Sergeev et al. (1992). Later definitions, emphasizing the rapid flux transport or RFT (Schödel et al. 2001), used the specific threshold value of the bursty convection electric field $E_{y}>2~\mbox{mV}/\mbox{m}$, equivalent to the transfer of 5 nT magnetic field at $V=400~\mbox{km}/\mbox{s}$. The use of the electric field as a threshold marker helps trace the flux transport from the tail to the inner magnetosphere where the flow bursts are slowed down well below 400 km/s. A similar concept of Flux Pileup Regions (FPRs) was introduced by Zhang et al. (2007) and later used in a number of studies (e.g., Khotyaintsev et al. 2011) (see, also Sect. 4.4). Yet another definition emphasizes transient enhancements in $B_{z}$ in the current sheet center ($B_{x}=0$) by focusing on the earthward-contracting dipolarizing flux bundles or DFBs (Liu et al. 2013), which has advantages in describing the dipolarization process during substorms. In the following we use the aforementioned acronyms intermittently in the appropriate specific context.

The earthward-moving transients are most extensively studied in the radial distance region $10\mbox{--}30\,\mbox{R}_{E}$ with the following distinctive features: (1) BBFs are associated with an increase in the northward magnetic field component ($B_{z}$) and a decrease in plasma density and pressure (e.g., Ohtani et al. 2004; Runov et al. 2011, 2015). (2) BBFs represent narrow flow jets with a cross-tail scale of $1\mbox{ to }5\,\mbox{R}_{E}$ (e.g., Nakamura et al. 2004; Liu et al. 2013, 2015). (3) BBFs are most frequently observed in the pre-midnight magnetotail sector (see Walsh et al. 2014, for a review). (4) BBFs are commonly associated with energetic particle flux increases (the latter are discussed in more detail in Sect. 4.5). The observed BBFs have properties similar to those of the narrow fast plasma flow channels seen in global MHD simulations (Wiltberger et al. 2015) (see Sect. 4.3 for more detail).

**Fig. 29:**
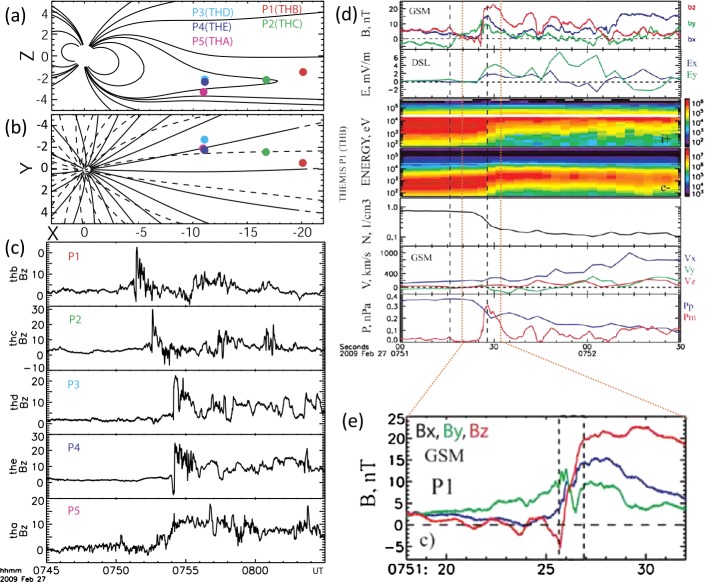
Observations of a DF by the fleet of 5 THEMIS probes distributed along the tail: (**a**)–(**b**) probe positions, (**c**) magnetic field $B_{z}$ GSM component recorded by Probes P1–P5, (**d**)–(**e**) zoomed-in $B_{z}$ variations, as well as accompanying variations (in panel (**d**)) of the ion energy-time spectrogram ($\mbox{eV}/\mbox{s}/\mbox{cm}^{2}/\mbox{eV}$), electron time-energy spectrogram, ion number density X, Y, and Z GSM components of the ion bulk velocity, magnetic (Pm) and plasma (Pp) pressures (adapted from Runov et al. 2009)

Earthward transients often look very spectacular in the leading part of the earthward fast flow burst, at the front which separates them from the ambient plasma ahead, and which is known as the dipolarization front (DF) (Nakamura et al. 2002b). DFs are seen in data as sharp increases of the northward magnetic $B_{z}$-component (i.e., transient dipolarization), accompanied by the plasma density and pressure drops (Fig. 29d) and often preceded by $B_{z}$ dips of smaller amplitudes. A typical time scale of the $B_{z}$ increase is as small as 1–3 seconds (Runov et al. 2009, 2011) (Fig. 29). Thus, DFs represent thin (ion-scale) current sheets with $j_{y}$ (up to several tens of $\mbox{nA}/\mbox{m}^{2}$ (Runov et al. 2011; Liu et al. 2013)), with the largest contribution coming from the $\partial{B_{z}}/\partial{x}$ term. In the presence of such a strong magnetic field gradient the ion and electron motions are decoupled, which leads to the generation of an electric field directed normal to the front. This Hall electric field was indeed observed and its amplitude was found to be as large as $10\mbox{ to }100~\mbox{mV}/\mbox{m}$ (Runov et al. 2011; Fu et al. 2012a). According to some estimates (Angelopoulos et al. 2013), the DFs could sometimes be the major dissipation sites in magnetotail during substorm, although other studies indicate that DFs have properties close to those of tangential discontinuities (Fu et al. 2012a).

DFs are usually observed as isolated soliton-like structures inside BBFs (e.g., Runov et al. 2009; Angelopoulos et al. 2013). However, there were also observations of groups of multiple DFs (Hwang et al. 2011), which can be explained using simple buoyancy mechanisms (e.g., Guzdar et al. 2010). They are distinguished not only by sharp $B_{z}$ increases and density drops, but also by the sharp peaks of energy conversion with the rate up to $\mathbf{j}\cdot \mathbf{E}\sim 500~\mbox{pW}/\mbox{m}^{2}$ (Huang et al. 2015; Khotyaintsev et al. 2017).

A superposed epoch analysis, using DFs as markers, revealed that plasma bulk flow appears about 50 s prior to the front detection, and the plasma velocity, similar to the density and ion temperature gradually increases during this time interval (Ohtani et al. 2004; Runov et al. 2011). Within the MHD framework, these precursor flows may be understood as a consequence of the plasma compression ahead of the front, which locally reduces the pressure gradient and leads to a net $\mathbf{j}\times \mathbf{B}$ force (Li et al. 2011; Birn et al. 2015a). On the kinetic level, the appearance of hot earthward streaming particles may be thought as a result of particle reflection by the enhanced magnetic field of the earthward-propagating front—a moving-mirror acceleration (Zhou et al. 2010, 2011). This process may also explain the field-aligned beams of hot ions observed in the plasma sheet boundary layer (PSBL) ahead of the DFs (Zhou et al. 2012).

**Fig. 30:**
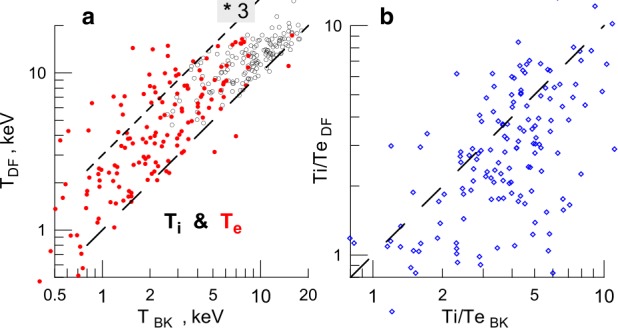
Comparison of (**a**) the ion $T_{i}$ and electron $Y_{e}$ temperatures, as well as (**b**) their ratio $T_{i}/T_{e}$ immediately following after the dipolarization front (index DF) with their background values preceding the front (index BK) (Sergeev et al. 2015)

In contrast to ions, the electron temperature increases sharply at the front, reaches a maximum at the $B_{z}$ peak and either stays on that level or gradually decreases while a DFB passes by a probe (Runov et al. 2011) resulting in a sharp drop of the temperature ratio $T_{i}/T_{e}$ (Runov et al. 2015; Sergeev et al. 2015) (Fig. 30). Thus, to a large extent, the dipolarization fronts separate two plasma populations: the ambient plasma sheet and hot, tenuous plasma in DFBs. The magnetic variation at the front is supported by the diamagnetic current, flowing on the boundary of tenuous DFB plasma and denser and cooler ambient plasma sheet (Runov et al. 2011; Zhang et al. 2011).

That DFB plasma and ambient population are not totally separated, becomes clear from comparative studies of particle energy spectra in DFBs and in the ambient plasma sheet, which revealed a close inter-relationship between two populations (Runov et al. 2015). This could be a consequence of limited cross-tail extent of DFBs, permits entry of plasma into (and exit from) the DFB by cross-tail drift, enabling a mixing of populations. These entry and exit processes will be further discussed in Sect. 4.5.

Fast earthward flows eventually brake in the near-Earth region (Shiokawa et al. 1997). Recent MMS observations of the flow-braking process off the neutral plane with $\sim50\text{ km}$ probe separation (Nakamura et al. 2018) revealed the formation of highly structured field-aligned currents and Hall current layers.

*Key points:*

(1) Earthward flow bursts are associated with an increase in the northward magnetic field component $B_{z}$ and a decrease in plasma density and pressure. They represent narrow flow jets with a cross-tail scale of $1\mbox{--}5\,\mbox{R}_{E}$. They are most frequently observed in the pre-midnight magnetotail sector and associated with energetic particle flux increases. (2) Leading parts of the flow bursts often contain substructures with sharp front boundaries, dipolarization fronts or DFs, at which the magnetic field $B_{z}$ rapidly (in $\sim1\text{ s}$ corresponding to one thermal ion gyroradius in space) increases, while plasma density and pressure drop. (3) DFs have 50 s-long precursors, presumably formed by ions reflected from the DF. Outside of the CS the DF precursors resemble field-aligned PSBL ion beams. (4) Behind DF electrons are heated much stronger than ions, resulting in a sharp drop of the temperature ratio $T_{i}/T_{e}$.

*Open questions:*

What are roles of DFs and X-lines in the substorm activity of the magnetotail? In particular, may DFs appear without substantial signatures of magnetic topology changes?

What are the collisionless dissipation mechanisms in DFs/BBFs/FTRs/DFBs or are they just ideal-MHD structures whose dissipation can only be provided by particle collisions in the ionosphere?

### Observations of Tailward Transients

Whereas post-onset transients in the near-Earth plasma sheet have been extensively studied, mid-distant tail ($30< R<100\,\mbox{R}_{E}$) dynamics remains under-explored. Post-ISEE3/Geotail view of activity in this region, summarized in the review paper by Sharma et al. (2008), was largely focused on the plasmoid/flux rope elements, which were thought to extend over a large portion of the magnetotail in the dawn-dusk direction (e.g., Slavin et al. 2003). In particular, using the Geotail dataset Ieda et al. (1998) reconstructed statistically the plasmoid evolution during its tailward motion from $20\mbox{ to }210\,\mbox{R}_{E}$ along the aberrated tail axes. According to their results, typical plasmoid length varies from $10\,\mbox{R}_{E}$ in the mid-tail and up to $40\,\mbox{R}_{E}$ in the distant tail, which is comparable to entire width of the magnetotail.

The picture of midtail transients has recently been strongly enriched due to the analysis of data from two ARTEMIS probes which surveyed the magnetotail at lunar distances with varied interspacecraft separations. In this way Kiehas et al. (2013) found that, contrary to previous results, flux rope cross-tail extent is limited by a few $\mbox{R}_{E}$, and, similarly, Li et al. (2014b) found that typical plasmoid cross-tail scale is smaller than $5\,\mbox{R}_{E}$. (Exceptions were observed during strong magnetospheric activity, $\mathit{AE}>50~\mbox{nT}$).

An important ARTEMIS update is also that the probabilities to observe tailward $V_{x}<0$ and earthward $V_{x}>0$ fast flows are nearly equal, with slightly larger probability to observe $V_{x}<0$ in the high speed region $|V|>300~\mbox{km}/\mbox{s}$ (Kiehas et al. 2018). It was confirmed that earthward and tailward flows are associated with northward ($B_{z}>0$) and southward ($B_{z}<0$) magnetic field, as predicted by magnetic reconnection models of the tail activity.

**Fig. 31:**
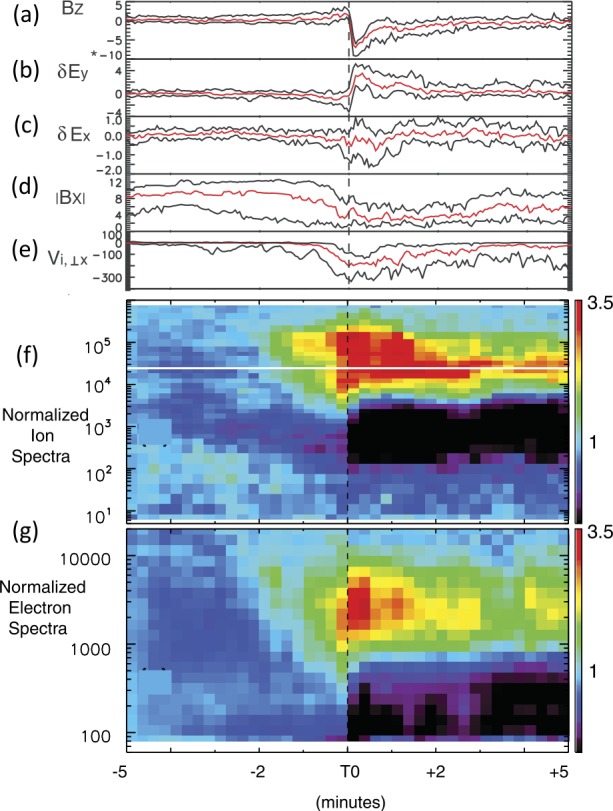
Superposed epoch analysis of the (**a**, **d**) magnetic field, (**b**, **c**) electric field, (**e**) ion velocity, as well as normalized (**f**) ion and (**g**) electron energy flux spectrograms for 26 ADFs (Li et al. 2014a)

ARTEMIS observations further revealed that fast tailward flows often carry sharp, highly asymmetric north-then-south variations in $B_{z}$ that do not fit the classical plasmoid model, but look rather as the mirror images of earthward-moving DFs (Fig. 31). They were coined therefore anti-dipolarization fronts or ADFs (Li et al. 2014a; Zhou et al. 2015). In fact, Angelopoulos et al. (2013) interpreted an ADF as a proto-plasmoid, that is, the leading edge of a developing plasmoid. The flux transfer rate $E_{y}$ rapidly increases at the ADFs and then gradually decreases down to undisturbed value, the peak $E_{y}$ amplitude often exceeds 2 mV/m. The average duration of these flux transfer events is 50 to 100 s, being close to those in the earthward-moving DFBs (J. Liu et al. 2014a). Statistically, the tailward convective flows are most probable between $Y_{\mathrm{AGSM}}$ of $5\mbox{ to }8\,\mbox{R}_{E}$ (Kiehas et al. 2018), like the earthward BBFs and other reconnection-related phenomena in the near-Earth plasma sheet (see Walsh et al. 2014, for a review).

Figure 31 also shows that the particle energy spectra on both sides of the sharp ADFs are different, indicating the separation of two plasma populations at the fronts. These signatures differ from those in classical plasmoid models, which exhibit a hot plasma population with no boundary at the center (see Li et al. 2014a, for comparisons between ADF and plasmoid observations). The distinct populations ahead and behind the ADFs, according to Li et al. (2014a), are the compressed ambient plasma sheet population and the heated plasma within newly-reconnected field lines, respectively. One can also find in Fig. 31 the enhancement of energetic ion fluxes before the ADF arrival, which probably originates from the acceleration of ambient ions during their reflection at the fast-propagating ADFs (Zhou et al. 2015). These signatures also indicate the similarities between ADFs and their mirror images of earthward-propagating DFs.

Runov et al. (2018) compared the plasma properties of tailward flux transfer events observed by ARTEMIS at lunar orbit with those in DFBs observed in the near-Earth plasma sheet by THEMIS probes. They found that the average ion temperature $\langle{T_{i}}\rangle\approx4~\mbox{keV}$ is the same as that in DFBs observed at radial distances $15< R<25\,\mbox{R}_{E}$, despite the ambient plasma sheet temperatures at these two locations being very different. The ion spectra at both locations were characterized by similar kappa-function with $\kappa\sim 5\mbox{ to }6$. Thus, statistically, the same ion population is observed in earthward and tailward outflows as observed at $60\,\mbox{R}_{E}$ and at $15< R<25\,\mbox{R}_{E}$. Assuming that the plasma sheet with temperature $\langle{T_{i}} \rangle$ and density $\langle{n}\rangle=0.2\text{ cm}^{-3}$ (Runov et al. 2015) was in balance with the lobe magnetic field $B_{L}$, Runov et al. (2018) concluded that the ion populations in both outflows originated at geocentric distances $25\mbox{ to }30\,\mbox{R}_{E}$, which is consistent with the most probable location of the near-Earth reconnection site (e.g., Nagai et al. 1998, 2005). Yet, electron temperatures and spectra at $60\,\mbox{R}_{E}$ and in DFBs at $15< R<25\,\mbox{R}_{E}$ were found to be quite different.

Observed similarities between the earthward-contracting DFBs in the near-Earth plasma sheet and tailward outflows suggests that these two phenomena originate in the same process that operates in impulsive regime and located in the mid-tail at $R\sim 30\,\mbox{R}_{E}$. The observations are consistent with the concept of time-dependent, impulsive reconnection (e.g., Sergeev et al. 1992). It should be noted, however, that it would be incorrect to state that the dipolarization fronts are directly created by reconnection. The fronts are current sheets separating two plasma populations with different densities and temperatures. Thus, the fronts’ appearance does not require reconnection, they may appear, for example, in the course of the ballooning-type instability development (e.g., Pritchett and Coroniti 2010; Pritchett 2013).

*Key points:*

(1) In contrast to earlier results based on the single-probe analysis, data from two ARTEMIS probes show that the cross-tail extension of the flux ropes at lunar distances is limited to a few $\mbox{R}_{E}$. (2) Probabilities to observe tailward $V_{x}<0$ and earthward $V_{x}>0$ fast flows are nearly equal at $\sim60\,\mbox{R}_{E}$. (3) Tailward flows often carry sharp, highly asymmetric north-then-south variations in $B_{z}$ that do not fit the classical plasmoid model, but look rather as the mirror images of earthward-moving DFs; they are interpreted as proto-plasmoids. (4) Tailward and earthward flows have similar dawn-dusk distributions of the plasma parameters suggesting that they originate in the same process (most likely, magnetic reconnection) that operates in impulsive regime and located in the mid-tail at $R\sim30\,\mbox{R}_{E}$.

*Open questions:*

1. If the interpretation that ADFs are proto-plasmoids is correct, how does the sharp boundary evolve and dissipate during its tailward propagation? If not, what causes the difference between these two kinds of structures?

2. What causes the difference between electron spectral properties in earthward and tailward outflows, whereas the ion properties are approximately the same? How do the outflow structures interact with the ambient plasma sheet, and how do we describe the ion and electron dynamics in these structures?

### Simulations of Magnetotail Transients

Regional MHD magnetotail simulations (Birn et al. 2004a, 2011) have demonstrated that azimuthally localized BBFs and DFs are generated as part of the earthward propagation of entropy depleted flux tubes. The depleted flux tubes (or “bubbles”) can be created either by reconnection or an ad hoc reduction of the flux tube entropy content further in the tail. As the flows brake approaching the dipole field region, they expand azimuthally and rebound creating vortical flow structures outside of the earthward flow channels.

Signatures of similar transient magnetotail flows and corresponding magnetic field perturbations have been seen in global MHD simulations of the magnetosphere since their relatively early days (Wiltberger et al. 2000). More recently, due to improved resolution and overall quality of the models, these transient phenomena in the simulations have acquired a significant degree of realism (Ge et al. 2011; El-Alaoui et al. 2013; Wiltberger et al. 2015). In global simulations, the fast flow channels are produced by reconnection and accelerated toward Earth by the magnetic tension force (e.g., El-Alaoui et al. 2016).

Hu et al. (2010) presented global simulations using the OpenGGCM one-way coupled with the RCM model of a substorm event. They showed that localized reconnection in the mid tail produced flux tubes with significant flux tube entropy depletion. While these flux tubes were initially accelerated by the reconnection process itself, the depletion allowed them to penetrate close to Earth, i.e., to within geosynchronous altitude. Since the RCM can produce energy spectra, this study also compared the simulation results directly to geosynchronous observations from LANL satellites for energies between 50 and 315 keV. The simulated time series of energized particles closely resembled the observed ones, suggesting that substorm injections were a result of BBFs.

Ge et al. (2011) used the OpenGGCM to simulate another substorm, for which many space and ground based data were available. The simulation results revealed a remarkable complexity of BBF flows, which are in no way simple straight flow channels, as they are often depicted, but are winding their way through the tail as shown in Figs. 12–14 of that paper. At present, the complex topology of the flows predicted by the global simulations eludes verification, and will require a satellite constellation to become observable.

**Fig. 32:**
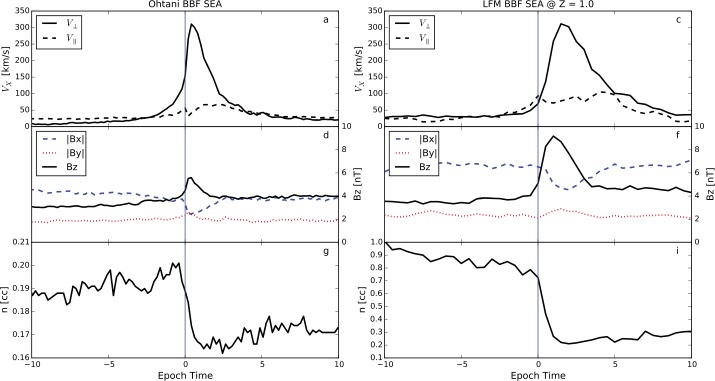
Superposed epoch analysis of BBF properties inferred from Geotail observations (Ohtani et al. 2004) and from LFM simulations. The top panel shows the $V_{x}$ component of the velocity perpendicular to the magnetic field; the middle panel shows the magnetic field components; and the bottom panel shows the plasma density. The LFM results were extracted from the idealized LFM simulation at $z=0$. The figure is adapted from Wiltberger et al. (2015)

Similar complexity of plasma sheet convection in the form of BBFs was demonstrated in LFM simulations by Wiltberger et al. (2015). They performed a superposed epoch analysis (SEA) of BBFs in the high-resolution LFM global MHD simulation. The criteria for the SEA were adopted from the statistical analysis of Geotail data by Ohtani et al. (2004), and the corresponding results are compared in Fig. 32. The left column is reproduced from the work of Ohtani et al. (2004), while the right column shows the simulation results in the same format. There is significant consistency between the observed and simulated signatures. The shape of the plasma velocity profiles in the top panel agrees well, including both the width and the amplitude. One difference is that the rise of the perpendicular $V_{x}$ component starts prior to the zero epoch time in observations (left column), which might be attributed to ions reflected off of the front—an effect not included in the single-fluid MHD description of the global model. The magnetic field $B_{z}$ component profile is also largely similar, with the $B_{z}/|B_{x}|$ showing differences due to sensitivity to the location of the spacecraft (or of the fiducial point in the simulation) relative to the neutral plane. Perhaps, the most significant difference can be seen in the bottom panel of the figure depicting the plasma density (note the different scales on the vertical axis.) This discrepancy was attributed to the preconditioning of the plasma sheet in the idealized MHD simulation with northward IMF driving that controlled the density of the plasma sheet through which the DF was propagating. Qualitatively, however, both observations and simulations reveal a drop in the plasma density behind the DF. Overall, the general statistical agreement of the simulation results with the data suggests that the transient features produced by the simulations are similar phenomena to those observed.

The complexity of the BBFs and their effects on the inner magnetosphere was further demonstrated by Cramer et al. (2017). Using OpenGGCM simulations with full two way coupling with the RCM they first showed that BBFs, as defined by their flow, are indeed depleted flux tubes. This was demonstrated not just for a few events, but for a number of storm and quiet time simulations over long time periods with 100 s of BBFs. They further showed that collectively the BBFs were responsible for roughly 80% of plasma transport into the ring current during storm times. The BBF dominated transport dropped off from 8 RE inwards, but still provided more than 50% at 6 RE and 10–20% at 4 RE. It must be noted, however, that it becomes more difficult to identify BBFs closer to Earth as their speed decreases. Still, the study confirmed previous works (e.g. Yang et al. 2015) suggesting that steady convection is not a viable process to feed the ring current, as originally predicted by Erickson and Wolf (1980), but instead, flux tubes must be depleted in B/I unstable regions to be able to penetrate deep into the magnetosphere.

Ukhorskiy et al. (2018) used the high-resolution global magnetosphere LFM simulation by Wiltberger et al. (2015) to demonstrate the process by which the magnetic structures associated with BBF’s (i.e., DFs) bring energetic ions from the mid-tail into the ring current. In their simulations, they used test particles traced in the MHD fields to show that protons with initial energy above 5–10 keV exhibited magnetic trapping which allowed them to be transported together with the bulk plasma and penetrate deep into the inner magnetosphere ($\sim4\,\mbox{R}_{E}$). In the process, the trapped particle population was accelerated by a factor of 10, and their contribution to the overall ring current buildup was in the range 20–60%.

Furthermore, Sorathia et al. (2018) used test particle tracing in two-way coupled LFM-RCM simulations of a geomagnetic storm completed earlier by Wiltberger et al. (2017) as well as the standalone high-resolution LFM simulation by Wiltberger et al. (2015) to demonstrate that plasma sheet electrons can also exhibit magnetic trapping in magnetic structures of BBFs. Their simulations reproduced with high degree of accuracy the electron fluxes observed by the Van Allen Probes. They attributed the replenishment of the outer radiation belt after the initial depletion to a handful of discrete injections from the magnetotail and showed that mesoscale magnetic structures are efficient accelerators of electrons into the outer radiation belt.

*Key points:*

(1) Modern global MHD simulations produce BBFs and DFs with properties statistically similar to those observed. (2) These azimuthally localized structures are effective in transporting and energizing both ions and electrons from the plasma sheet into the ring current and radiation belts.

*Open questions:*

What non-MHD features of magnetotail transients are globally important, i.e., affect their dynamics on a global scale?

Is it possible to quantify collisionless dissipation in global simulations of BBFs and how can it be incorporated?

What is the role of the energy-dependent drifts in the evolution of magnetotail transients? In particular, what is the role of the transition region between the tail-like and dipole magnetic field where both the drifts and high-speed flows are important? How important are 3D particle dynamics and non-adiabatic effects, and how can they be incorporated into global MHD models?

### Micro-instabilities

Micro-instabilities long have been considered as a mechanism for generating magnetotail explosions. In particular, the associated anomalous resistivity could enable the resistive tearing mode and magnetic reconnection (e.g., Huba et al. 1977, 1980). Such a mechanism could be an alternative to reconnection onset models starting from kinetic tearing instabilities driven by linear Landau dissipation that have been discussed in Sects. 3.1–3.3. While the classic ion-acoustic instability was found not to operate under realistic magnetotail plasma conditions ($T_{i} > T_{e}$), a very thin tail current sheet and strong reduction of $B_{z}$ can potentially enable micro-instabilities such as the modified two-stream or the lower-hybrid drift instability. The lower-hybrid drift instability (LHDI) has been studied extensively in PIC simulations (e.g., Lapenta et al. 2003; Daughton et al. 2004; Ricci et al. 2004; Sitnov et al. 2004). For drastically thinned (sub-proton gyroradius) CSs, they might penetrate to the center and/or cause anisotropic heating of electrons and thereby dramatically increase collisionless tearing (e.g., Daughton et al. 2004). Other modes have also been considered (e.g., Büchner and Kuska 1999; Yoon et al. 2002) and reviewed relatively recently by Büchner and Daughton (2007). Meanwhile, it was also found that micro-instabilities are strongly stabilized by the finite $B_{z}$ magnetic field component which magnetizes electrons (Pritchett and Coroniti 2001; Pritchett 2002). Based on recent MMS observations, whistler modes have also been suggested to enable or modify reconnection at the magnetopause (Cao et al. 2017). For ion scale current sheets, LHD modes typically predominate, and such modes have been observed in the PSBL. However, MMS observations have not (yet) confirmed the role of LHD waves in the electron diffusion region in the tail. In the following we therefore focus particularly on micro-instabilities in plasma transients away from the tail reconnection site.

Dynamic evolution of the magnetotail leads to creation of strong gradients at kinetic scales and strongly anisotropic particle distributions, for example temperature anisotropies. They provide energy sources for micro-instabilities which limit steepening of the gradients and further development of the anisotropies. In this section we give several examples of such instabilities which impact the front evolution.

**Fig. 33:**
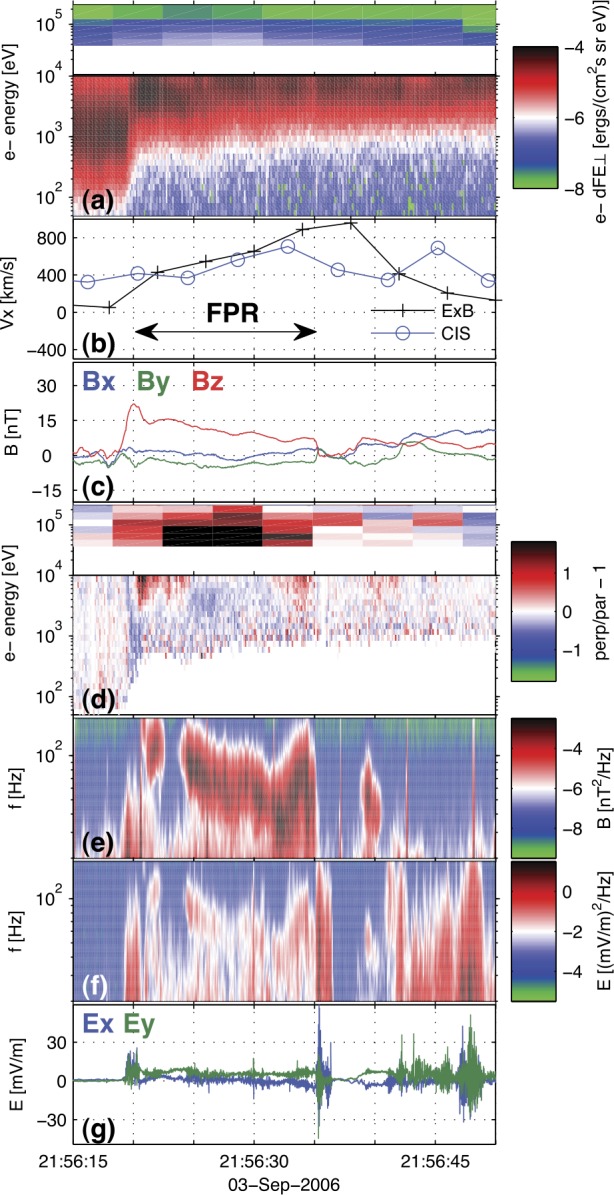
FPR observed by Cluster C1. (**a**) The electron flux from the Research with Adaptive Particle Imaging Detectors (RAPID, $>30~\mbox{keV}$) and Plasma Electron and Current Experiment (PEACE, $<10~\mbox{keV}$). (**b**) GSM X component of the ion flow from the Hot Ion Analyzer (HIA) of the Cluster Ion Spectrometry (CIS) experiment and $E \times B$ from the Electric Field and Wave (EFW) and fluxgate magnetometer (FGM) experiments. (**c**) Magnetic field GSM components from FGM. (**d**) Electron flux anisotropy, zero corresponds to isotropic fluxes; fluxes below $10^{-6}~\mbox{ergs}/(\mbox{cm}^{2}\,\text{s}\,\mbox{sr}\,\mbox{eV})$ are excluded below 10 keV. (**e**) Magnetic field spectrum (20–180 Hz) from the Spatio Temporal Analysis of Field Fluctuations (STAFF) experiment. (**f**) Electric field spectrum and (**g**) waveform. Adapted from Khotyaintsev et al. (2011)

Figure 33 shows an example of micro-instabilities observed by Cluster in association with a flow burst on September 3, 2006 (Khotyaintsev et al. 2011). The Cluster satellites were initially in the central plasma sheet where they detected a fast earthward plasma flow reaching maximum speed above 800 km/s at 21:56:35 UT (Fig. 33b). Prior to the flow maximum, a sharp $B_{z}$ increase (DF, Fig. 33c). The DF was propagating Earthward with a speed of $\mathbf{V} = 450 \cdot[0.91, 0.41, 0.08]~\mbox{km}/\mbox{s}$ GSM. A flux pileup region (FPR, marked by arrow in Fig. 33b) is formed between the DF and the peak of the flow velocity; there the plasma flow velocity increases and the magnetic field decreases consistent with braking of the plasma flow and pile-up of the magnetic flux.

The DF and FPR are associated with strong wave activity in both electric and magnetic fields. Strong, primarily electrostatic, broadband wave activity covering the lower-hybrid (LH) frequency range (Fig. 33g), $f_{\mathrm{LH}} \sim5\mbox{--}15~\text{Hz}$, is observed at the DF (21:56:20 UT) and the rear edge (21:56:35 UT) of the FPR. Peak values of the electric fields reach above 60 mV/m. Such wave activity in the LH frequency range contains the strongest electric fields observed in association with DF, and have been reported on a number of occasions (Sergeev et al. 2009; Divin et al. 2015b). The observed LH-range waves are localized to a spatial region having the transverse scale $\sim 500~\mbox{km}$, $\sim c/\omega_{pi}$, which contains the steepest gradients of the magnetic field and plasma density. This suggests that drift-type instabilities, such as the LHDI, can be responsible for the observed wave activity.

Observations of the electric and magnetic field fluctuations allow one to determine the phase velocity and wavelength of these waves. Since ions are unmagnetized in the lower hybrid frequency range $f_{ci}< f< f _{ce}$, electrons will carry a current via $\delta E \times B_{0}$ drift, where $\delta E$ is the wave electric field. And this current will produce a fluctuating magnetic field in the direction parallel to the ambient field, $\delta B _{\parallel}$. These magnetic fluctuations are linearly related to the electrostatic potential of the wave, which allows determination of the phase velocity vector (Norgren et al. 2012). For a DF event observed by Cluster, Divin et al. (2015b) assessed the frequency of oscillations in the reference frame moving with the front, and the wave vector, $|k_{\perp} \rho_{e} | \sim0.5$; the wave vector has a negative $k_{x}$ component, i.e., it is pointing toward the flux pile-up region. From comparison of the obtained wave characteristics with solutions of the theoretical dispersion relation one can conclude that the observed electric field oscillations are generated by the LHDI. The instability is driven by the strong density gradient at the front, and the observed amplitudes of the oscillations suggest that the instability already reached a nonlinear phase when many key parameters are saturated.

Electric fields associated with LHDI with $k_{\perp} \rho_{e} \sim0.5$ have the largest amplitudes. But the typical electric field spectrum at a DF is rather broadband, meaning multiple wavelength are present in the system consistent with continuous transition from LHDI to lower frequency kinetic B/I (Pritchett and Coroniti 2013). Recent MMS observations show evidence of a turbulent LHDI cascade from $k_{\perp} \rho_{e} \sim0.5$ to shorter wavelengths (Pan et al. 2018). Also longer, ion-scale fluctuations have been reported from multi-spacecraft Cluster observations (Balikhin et al. 2014).

Electron temperature anisotropy with $T_{e\parallel}/T_{e\perp}>1$ is observed together with the waves at the DF (Fig. 33d), indicating possible electron heating by the waves leading to an increase of $T_{e\parallel}$. Cairns and McMillan (2005) proposed a mechanism for such heating, where LHDI can produce stochastic acceleration of electrons parallel to the magnetic field by the Cherenkov resonance. Such increase of $T_{e\parallel}$ associated with DF-driven LHDI has also been observed in PIC simulations (Divin et al. 2015a).

Observations of LHD waves at DFs on sub-proton scales using MMS 2016 data with $\sim55\text{ km}$ spacecraft separation have recently been performed by Le Contel et al. (2017). The MMS cluster was located at the plasma sheet edge where the LHD excitation could be particularly favorable due to the density gradient across CS as well as lower values of the plasma beta, in addition to the density drop along the tail at the front. Le Contel et al. (2017) found intense electric fluctuations normal to the magnetic field with the amplitude $200~\mbox{mV}/\mbox{m}$, four times larger than for similar LHD waves at the DF and near the equatorial plane (Divin et al. 2015b). About 0.5 s after the detection of LHD waves, a train of electromagnetic solitary waves (phase-space holes) with a 1 ms time scale was detected.

Whistlers represent another type of micro-scale waves that are frequently reported in association with DFs (Le Contel et al. 2009; Deng et al. 2010; Khotyaintsev et al. 2011). Viberg et al. (2014) based on statistical analysis of 9 years of Cluster observations concluded that whistlers are a characteristic signature of DFs. Whistler mode waves are common in the vicinity of DFs: between 30 and 60% of all DFs are associated with whistlers, and whistlers are about 7 times more likely to be observed near a DF than at any random location in the magnetotail. The distribution of whistlers at DFs is independent of the distance from Earth, along the $X_{\mathrm{GSM}} $ axis, between $-20\mbox{ and }{-}10\,\mbox{R}_{E} $. The median frequency of the whistlers was $0.16 f_{ce} $, with 75% being below $0.29 f_{ce} $.

An example of DF-related whistlers is shown in Fig. 33e, f, where electromagnetic waves at frequencies $\sim 100~\mbox{Hz}$ are observed behind the $B_{z}$ peak (inside the FPR). Consistent with the whistler-mode theory (e.g., Divin et al. 2015a, and refs. therein), these waves are circularly right-hand polarized and propagate close to the magnetic field-aligned direction (within $20^{\circ}$ as determined by the minimum variance analysis of $\delta B$). The waves have amplitudes up to 0.5 nT and $5~\mbox{mV}/\mbox{m}$.

The region of whistler waves (FPR) has the opposite sign of the electron anisotropy, compared to LHDI (Fig. 33d). Here we observe $T_{e\perp}/T_{e\parallel} > 1$ for energies above $\sim 3~\mbox{keV}$, with the anisotropy extending up to energies above 100 keV. To study the electron anisotropy, Viberg et al. (2014) defined a factor $\alpha= T_{e\perp}/T_{e\parallel} - 1 $, for $T_{e\perp} < T_{e\parallel}$ (and $\alpha= 1 - T_{e\parallel}/T _{e\perp} $ for $T_{e\perp}>T_{e\parallel} $), which has values between −1 and +1, where a negative $\alpha$ means more parallel than perpendicular flux, and vice versa. Based on Cluster statistics almost all whistlers are associated with $\alpha> 0$. This provides a strong indication that the whistler emissions are driven by perpendicular electron temperature anisotropy.

Poynting flux calculations demonstrate that the energy in the whistler mode leaves the current sheet and propagates along the background magnetic field, towards the Earth (Le Contel et al. 2009). This suggests that whistler mode waves are generated near the magnetic equator, where the anisotropy is maximum. Using multi-spacecraft observations by Cluster, Khotyaintsev et al. (2011) found that the generation region is located close to the neutral sheet, $B_{x} \sim0$, which has been confirmed by statistical results (Viberg et al. 2014). Generation of whistlers in the FPR was also observed in high-resolution PIC simulations (Fujimoto and Sydora 2008).

Apart from transporting energy away from the current sheet in a form of Poynting flux, whistler generation plays an important role for electron distributions in the FPR. Whistlers can modify the electron distribution on the time scale of seconds (Khotyaintsev et al. 2011) to reduce the anisotropy, which is consistent with the observed distributions typically being marginally stable. As the FPR magnetic field increases, betatron acceleration forces the particles to larger perpendicular energies. However, the anisotropy is eventually limited by the whistler-mode interaction, which predominantly scatters the electrons back to smaller pitch angles at supra-thermal energies. At higher (100 keV) energies, the wave-particle interaction is only efficient near 90^∘^ pitch angles, so the anisotropy in that range may exceed the whistler-mode marginal stability threshold without driving strong wave-particle interactions. This is consistent with observation of relatively large anisotropies at high energies (Fu et al. 2012b). Thus, whistler-mode waves through pitch-angle scattering make the betatron acceleration nonadiabatic and hence irreversible.

**Fig. 34:**
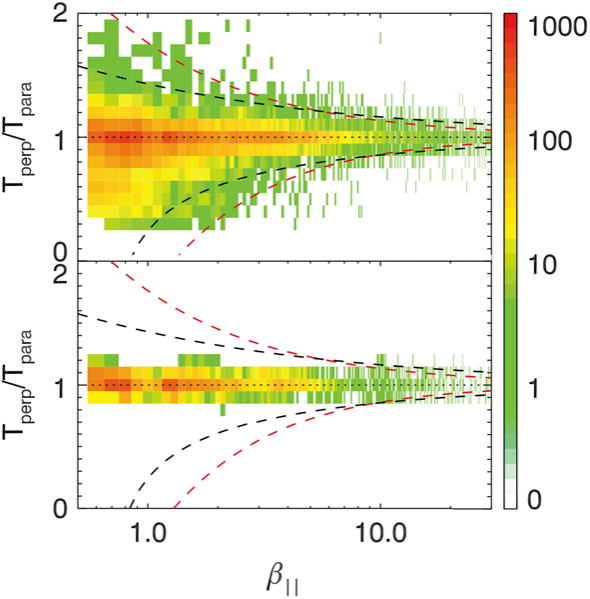
The top panel shows the statistic distribution associated with the BBFs of all events in the space ($\beta_{\parallel}$, $T_{i\perp}/T_{i\parallel}$), and the bottom panel is the statistic distribution associated with the quiet plasma sheet of all events in the space ($\beta_{\parallel}$, $T_{i\perp}/T_{i\parallel}$). Adapted from Wu et al. (2013b)

A significant fraction of the energy released by magnetotail reconnection goes to ion heating, but this heating is generally anisotropic. Compared to practically isotropic temperature in the quiescent tail, strong anisotropy increases are detected after a BBF passage. Such anisotropy can drive parallel and oblique firehose instabilities for $T_{i\perp}/T_{i\parallel}<1$, whereas the proton cyclotron and mirror instabilities are excited when $T_{i\perp}/T _{i\parallel}>1$. Using multi-point THEMIS observations, Wu et al. (2013b) analyzed relation of the observed anisotropies to the instability thresholds and found that the maximum anisotropies are indeed constrained by the instabilities (Fig. 34). They also found enhancement of magnetic fluctuations along the instability thresholds. However, Hietala et al. (2015) reported that localized deviation from this statistical results are possible. They observed patchy spatial regions with the anisotropy well above the firehose threshold in the interval $|B_{x} |=(0.1\mbox{--}0.5) B_{0}$, suggesting that the driver of the instability is strong while the instability is relatively weak to relax the anisotropy.

Zieger et al. (2011) presented Cluster observations of the interaction between the earthward moving fast plasma jet and the high-$\beta$ ambient plasma in the plasma sheet resulting in formation of non-linear mirror-mode structures and kinetic shocklets. Such mirror-mode structures have spatial scale of the order of the ion gyroradius. They develop within the plasma pileup region ahead of the jet front due to ion temperature anisotropy ($T_{i\perp} > T_{i||}$). Zieger et al. (2011) suggested that the growth of these mirror modes is driven by the perpendicular total pressure perturbation ($\Delta p _{\perp}$) generated by the braking jet. When $\Delta p_{\perp}$ becomes too large, the mirror-mode structure cannot maintain pressure balance any longer, and consequently a shocklet is formed in the pileup region ahead of the jet front.

*Key points:*

(1) Micro-instabilities in explosive dipolarizations are caused by sharp gradients and plasma anisotropies. (2) They include lower-hybrid drift instabilities at the DF and whistler instabilities behind it (FPR region), where electron distributions become more pancake-like because of the betatron effect. (3) Field-aligned anisotropy of the ion species drives parallel and oblique firehose instabilities, while their perpendicular anisotropy excites cyclotron and mirror instabilities. (4) Interaction of earthward-moving fast plasma jets with the high-beta ambient plasma results in the formation of nonlinear mirror-mode structures and kinetic shocklets.

*Open questions:*

What is the contribution of waves to the overall energetics of DFs, in particular which part of the energy is being transported away from the DF in the form of wave Poynting flux?

What is the efficiency of electron and ion heating by waves?

### Particle Acceleration and Velocity Distributions

The dawn-dusk electric field associated with dipolarization events (Sect. 4.1) has also been identified as a mechanism to accelerate ions and electrons to suprathermal energies. (For a recent review, see Birn et al. 2012.) The flux increases depend strongly on space and time. Acceleration mechanisms and particle behavior in general has become understood mostly from test particle studies in field configurations that were proposed to model dipolarization events or obtained from MHD simulations of such events, focused particularly on DFs and DFBs. In addition, particle characteristics in the vicinity of a reconnection site have been obtained from self-consistent PIC simulations (e.g., Hoshino 2005).

The acceleration mechanisms of electrons have been identified as betatron and first-order Fermi acceleration. At large pitch angles, electrons that are temporarily trapped within a DFB gain energy as the particle gyrates and drifts towards increasing magnetic field strength. For adiabatic electrons, the $\mathbf{E}\times\mathbf{B}$ drift toward increasing $B_{z}$ can also be expressed as gradient $B$ drift in the direction opposite to the dawn-dusk electric field (Birn et al. 2013). At low pitch angles, trapped electrons may bounce many times between mirror points in the more dipolar field and gain energy by the slingshot effect of first-order Fermi, type B, acceleration (e.g., Northrop 1963). This effect can also be expressed as curvature drift opposite to the dawn-dusk electric field (Birn et al. 2013). Whether betatron or Fermi acceleration dominates in shaping electron distributions, depends primarily on the location relative to the DFB. At higher latitudes, Fermi acceleration appears to be the dominant mechanism, while closer to the equator, betatron acceleration seems more important (Runov et al. 2013). However, the distance from Earth, or rather from the reconnection site, the presumed origin of the DFBs, also seems to play a role (e.g., Fu et al. 2011, 2012b; Wu et al. 2013a).

Recent MMS observations (Ergun et al. 2018) revealed an important role of the kinetic-scale turbulence in electron acceleration. Large-amplitude (up to $100~\mbox{mV}/\mbox{m}$) turbulent electric fields in the frequency range mainly above the average ion cyclotron frequency, associated with strong magnetic field fluctuations ($\delta B\sim20~\mbox{nT}$), were found to accelerate electrons to greater then 100 keV energies.

Ion acceleration can be more complicated and difficult to understand, primarily because ions in the energy range of interest (tens to hundreds of keV) are nonadiabatic and typically have only a single encounter or just a few encounters with the DF (or gyrations within the DFB). Ions may also be affected by direct acceleration in the vicinity of a near-tail $x$-line. When they are subsequently ejected along the magnetic field toward Earth, they tend to form a crescent-shaped beam population that co-exists with an unperturbed cold lobe population (Birn et al. 2015b), as observed by Zhou et al. (2012) in the PSBL. However, similar ion populations can also result from a single encounter and reflection by a DF with subsequent ejection toward higher latitude (Zhou et al. 2012).

**Fig. 35:**
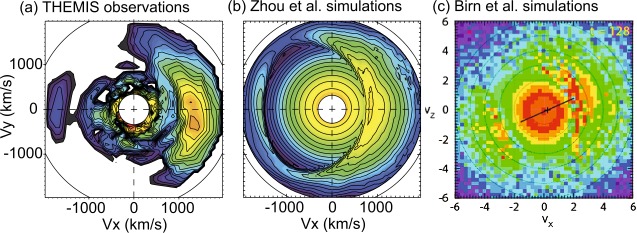
Crescent-shaped ion structures in the velocity space (**a**) observed in the plasma sheet boundary layer, in comparison with simulations in (**b**) Zhou et al. (2012) and in (**c**) Birn et al. (2015b)

Figure 35 presents a comparison between observations and simulation results of the crescent-shaped ion distributions in the PSBL. The similarities between observations and simulations, together with the observed timing correlation between DFBs and PSBL ion beams (Zhou et al. 2012), suggests that these transient crescent-shaped ion beams are closely associated with near-tail reconnection and DFBs. These results are similar to earlier findings of crescent shaped PSBL ion beams resulting from quasi-steady reconnection or retreating reconnection sites in the distant tail (Forbes et al. 1981; Onsager et al. 1991). Somewhat deeper within the plasma sheet, return beams and even multiple-energy beams might be observed, which result from mirroring near Earth and presumably two or more encounters with a propagating DF (Birn et al. 2017b).

Closer to the plasma sheet center, energetic ion populations show different anisotropies. Just prior to the arrival of a DF, earthward streaming populations are observed, contributing to the net earthward plasma flow (Zhou et al. 2010; Wu and Shay 2012; Drake et al. 2014; Greco et al. 2014; Birn et al. 2015a; Eastwood et al. 2015; Li et al. 2017). Such populations were found to result from a single encounter and reflection of ambient ions at a DF (Zhou et al. 2011; Birn et al. 2015a).

**Fig. 36:**
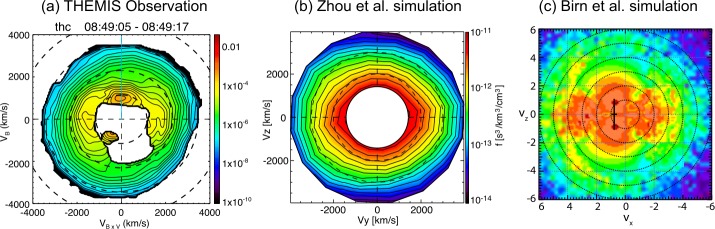
Perpendicular anisotropic ion distributions inside the DFB (**a**) observed by THEMIS spacecraft (Runov et al. 2017a), in comparison with simulations in (**b**) Zhou et al. (2018) and in (**c**) Birn et al. (2017a)

Right after the passage of a DF, ion distributions obtained by THEMIS observations are found with a strong perpendicular anisotropy (Runov et al. 2017a). The nature of the ion anisotropy was again explored using test-particle simulations (Birn et al. 2017a; Zhou et al. 2018), which suggest that the anisotropy originates from betatron-like acceleration in the propagating DFB electric field. Such ion distributions are shown in Figs. 36a–c.

The simulated ion distributions, shown in Figs. 36b and 36c, are quite consistent with the observations. Given that the DFB ions are a free energy source for instabilities when they are injected towards Earth, these may shed new lights on the coupling process between magnetotail and inner magnetosphere.

In contrast to the acceleration mechanisms, the source regions and entry mechanisms of ions into the acceleration region of the DFB are more controversial. The reason for this controversy is the strong spatial localization of the DFB in $x$ and $y$ and the fact that the entry depends strongly on the magnetic and electric field configuration outside the DFB, which is less well established observationally. Ambient plasma sheet particles may be picked up by the DFBs, become energized, drift across the DFB, and, eventually, exit from the DFB (see, e.g., Gabrielse et al. 2012; Birn et al. 2012, 2015a, for model description of these processes). In models, where the electric field vanishes outside the DFB, adiabatic particles on equatorial 90 degree pitch angle drifts simply follow contours of constant magnetic field ($B_{z}$ in most models) and enter mainly by azimuthal drift. After exit from the DFB they again follow azimuthal drift paths. Notably, this process that converts the electromagnetic energy into the thermal energy, also leads to locally built dawn-dusk asymmetry in thermal pressure: ions drift duskward, whereas lighter electrons drift dawnward (e.g., Runov et al. 2017b).

The surrounding electric field also affects the population ahead of a DF. For instance, in the Zhou et al. (2011) model, the electric field earthward of the DF is neglected. Thus the inferred precursor ion population consists of the superposition of reflected beam ions and an unperturbed plasma sheet population. In contrast, the Birn et al. (2015a) simulations, based on MHD fields, include a weak earthward flow and associated electric field ahead of the DF. This causes a weak acceleration of the preexisting population, which, combined with the higher-energy reflected ions, leads to a shifted nearly isotropic distribution.

Different conclusions about the entry of ions into the DFB just behind the front are also based on differences in the background electric field. In the Zhou et al. (2018) model, the ions experience successive ion reflections and reentries, and acceleration in the perpendicular direction, when they enter the DFB electric field from the earthward side, due to an assumed background electric field. This entry mechanism was used to explain an apparent relationship between the observed anisotropy of post DF ion distributions and the $\kappa$ value (square root of magnetic field curvature radius over gyro radius) of the ambient plasma sheet medium; for events with lower $\kappa$ values, the DFB ions appear to be more isotropic. In contrast, in the Birn et al. (2017a) simulations, which were based on MHD simulation fields (see, also, Fig. 36c), the accelerated ions entered the DFB electric field region mostly via cross-tail drift from the dawn flank outside of the DFB. (These simulations also showed low-energy field-aligned beams, generated by Fermi-type acceleration of low-energy ions entering from the lobes or PSBL, which, however, are harder to observe.)

*Key points:*

(1) In spite of different regimes, mostly adiabatic for electrons and often nonadiabatic or quasi-adiabatic for ions, the main acceleration mechanisms in substorm dipolarizations are betatron and first-order Fermi acceleration. (2) The specific dominant acceleration mechanism depends on the latitude, distance from the Earth or from the reconnection site. (3) Quasi-adiabatic features of the ion dynamics cause the formation of characteristic crescent-shaped PSBL distributions that co-exist with unperturbed cold populations. (4) In contrast to the acceleration mechanisms, the source regions and entry mechanisms into the acceleration region remain less clear and rather controversial because of strong localization of dipolarizing flux bundles and uncertainties of entry mechanisms.

*Open questions:*

How do the simulated electron and ion velocity distributions vary in space and time?

How do they affect stability and the generation of waves? And how are they affected by small-scale waves and turbulence?

What are the features and the role of heavy ions?

### Near-Earth Interchange Flux Tube Oscillations and Ionospheric Dissipation

The occurrence rate of earthward moving magnetotail transients or rapid flux transport events, discussed in Sect. 4.1, is found to decrease earthward of $\sim15\,\mbox{R}_{E}$ distance (e.g., Schödel et al. 2001), indicating slowdown and stopping in the inner tail. This might happen a few $\mbox{R}_{E}$ outside of geosynchronous orbit (Dubyagin et al. 2011; Runov et al. 2014), although dipolarization and energetic particle injection events (Sect. 4.5) have been observed to penetrate inside of geosynchronous orbit also (Friedel et al. 1996; Liu et al. 2016; Turner et al. 2017; Mitchell et al. 2018; Ohtani et al. 2018). The earthward motion of flow bursts and dipolarizing flux bundles is believed to end when the value of the moving plasma flux tube entropy $S$ (see Sect. 2.2.1) becomes close to the entropy of the background plasma sheet (Birn et al. 2009; Wolf et al. 2009; Dubyagin et al. 2011). Intrusions of fast flows into the inner magnetosphere and their direct correlation with particle injections are still very difficult to demonstrate observationally because of data paucity in the corresponding regions. At the same time, the observed correlation of the arrival of streamers at the equatorward edge of the auroral oval in the premidnight sector with enhancements in subauroral (westward) convection events, SubAuroral Polarization Streams or SAPS (Gallardo-Lacourt et al. 2017), enhancements in the proton aurora at subauroral latitudes (Nishimura et al. 2014) and omega band activity (Henderson 2012) suggest a prompt (on minutes scale) modification of inner magnetosphere convection in response to the intrusion of a magnetospheric flow channel.

The stopping and azimuthal diversion of earthward flows is believed to be essential for the build-up of the substorm current wedge; this has been the subject of a recent review (e.g., Kepko et al. 2015, and refs. therein) and will not be discussed here. It is suggested that a stopping flow burst is responsible for wave excitation in the Earth’s direction perpendicular to the magnetic field by driving a compressional (fast) wave front ahead of the flow (Kepko et al. 2001; Runov et al. 2014). If stopping flow bursts overshoot their equilibrium position, they may start performing interchange (or buoyancy) oscillations around that position (Wolf et al. 2012, 2018; Panov et al. 2013b). These oscillations are rather complex, but some recent studies have gained new knowledge for better understanding of their generation mechanism. For example, it has been revealed that their oscillation period is larger when flow burst stopping occurs farther from Earth at longer field lines (Wolf et al. 2012; Panov et al. 2014a). Also, due to asymmetry of the potential well in which the interchange oscillations occur, they can be anharmonic (Panov et al. 2015). Thin filament simulations predict more interesting properties of such magnetotail oscillations (Schutza 2015). These properties appear to heavily depend on the background plasma and magnetic field conditions. In contrast to the B/I oscillations growing in the azimuthally-wide magnetotail regions that are unstable due to locally inverse radial entropy profile (cf. Sect. 3.6), the interchange oscillations are damped oscillations of a depleted $2\mbox{--}3\,\mbox{R}_{E}$-wide flux tube that overshoot its equilibrium position in the dipolarizing magnetotail.

Different propagation paths of waves launched by a stopping flow burst are perhaps the reason for observations of the Pi2 pulsations through different L shells and wide sectors of magnetic local time (MLT) (Keiling et al. 2014). As BBFs are suddenly decelerated by the dominant dipolar magnetic field between $X=-20\,\mbox{R}_{E}$ and $X=-10\,\mbox{R}_{E}$, and pressure gradients pile up at the near-Earth edge of the plasma sheet, a substorm current wedge (Shiokawa et al. 1997; Baumjohann 2002; Birn et al. 1999; Birn et al. 2011; Ohtani et al. 2009) and substorm onset may occur. As ground observations by all sky imagers and magnetometers reveal, through coupling of stopping flow bursts and oscillating plasma sheet parcels with the ionosphere, braking plasma sheet flows also generate and modulate the ionospheric currents and aurora at the footprints of the field lines connected to the oscillating plasma sheet parcels (Panov et al. 2013a, 2016; Sergeev et al. 2014). It has also been shown statistically that such an oscillatory flow (and flux tube) braking (OFB) drives Pi2 magnetic field pulsations on the ground by launching waves along the field lines through twisting magnetic field lines on the sides of the flow channels (Panov et al. 2014b). The largest amplitudes of the Pi2 pulsations were detected near the footprints of the oscillating flux tube. The Pi2 pulsations during OFB are observed not only at a fundamental OFB frequency but also at a first harmonic (frequency doubling occurs) which is suggested to be the consequence of OFB anharmonicity (Panov et al. 2015).

**Fig. 37:**
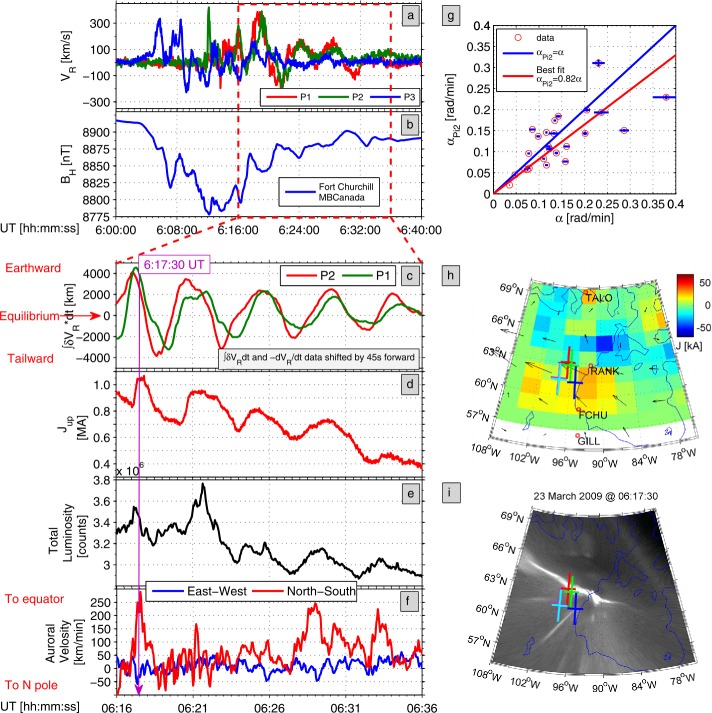
THEMIS space and ground observations on 23 March 2009 between 6:00 UT and 6:40 UT. (**a**) Radial ion velocity $V_{R}$ at P1 (red) and P2 (green) and P3 (blue) with positive values towards the Earth. (**b**) $B_{H}$ component of the magnetic field at Fort Churchill, MB, Canada. (**c**) Time-integrated oscillations of radial ion velocity $V_{R}$ at P1 (red) and P2 (green). (**d**) Total upward Jup SECS scaling factors around Rankin Inlet. (**e**) Total auroral luminosity from SNAP, RANK, and SNKQ. (**f**) Meridional (red) and longitudinal (blue) auroral velocity components. Auroral speed and velocity components were averaged over field of view of RANK. (**g**) Scatterplot of damping factors of Pi2 pulsations on the ground against damping factors of the oscillating velocity $V_{R}$ during 25 oscillatory flow braking events in the near Earth plasma sheet. Linear best fits are shown in red, assuming linear dependencies starting from ($0,0$). (**h**) Current system structure on the ground: snapshot of EICs (arrows) and the SECS scaling factors (colour: upward in reddish, and downward in bluish) at 6:17:30 UT, calculated using ground-based magnetometer array data. The footprints of THEMIS probes predicted by the AM-03 model are overplotted (red for P1, green for P2, blue for P3, cyan for P4, and magenta for P5). (**i**) Snapshot of all-sky camera observations of auroral activity at Rankin Inlet on 23 March 2009 at 6:17:30 UT. The figure panels were adapted from Panov et al. (2014a,b, 2015, 2016)

Figure 37a shows an oscillatory flow braking event on March 23, 2009 between 6:00 UT and 6:40 UT. The THEMIS probes’ footprints were identified to be between the Rankin Inlet and Fort Churchill in Canada (shown in Figs. 37h and 37i). Figure 37b shows the $B_{H}$ component of the magnetic field measured by the magnetometer at Fort Churchill which exhibits the Pi2 oscillations. Panov et al. (2014b) have compared the oscillation period and the damping factor of the plasma sheet flows with those of the Pi2 magnetic pulsations on the ground at auroral and midlatitudes near the local time of the conjugate ionospheric THEMIS footprints for 25 OFB events. Figure 37g shows scatterplot from Panov et al. (2014b) of the damping factor of the Pi2 pulsations $\alpha_{Pi2}$ against the damping factor $\alpha$ of the near-Earth oscillatory plasma flows observed by THEMIS with 95% confidence bounds and its linear best fit (red line). One can see that the best fit is close to $\alpha_{Pi2} = \alpha$ (blue line). Hence, the damping of the plasma sheet flows and of the pulsations on the ground occurs on the same time scales. Good correlation of damping factors of Pi2 pulsations on the ground with those of oscillatory flows in the near-Earth plasma sheet suggests that the oscillatory flows drive the ground Pi2 pulsations.

Further analysis of the oscillatory flow braking event on March 23, 2009 between 6:00 UT and 6:40 UT has shown that larger-amplitude ground pulsations at auroral latitudes were indeed caused by the oscillatory flow braking in the plasma sheet through alternating field-aligned currents as shown by Panov et al. (2016). Figure 37c shows $\int{\delta V_{R}dt}$—time-integrated oscillations of the radial ion velocity $V_{R}$, where $\delta$ indicates band pass filtering at periods between 10 and 500 s, and positive $V_{R}$ means earthward. The location of the oscillating magnetic flux tube with respect to its equilibrium position is indicated by $\int{\delta V _{R}dt}$.

As predicted by the AM03 model (Kubyshkina et al. 2011), the footprints of THEMIS probes P1 and P2 were at the auroral bulge location most of the time. That is, they were located between the red and blue spots of upward and downward ionospheric currents at the westward electrojet current (cf. Fig. 37h). The ground $J_{up}$ (Fig. 37d; obtained by integrating the reddish spot in Fig. 37h over surface) reveals significant (up to $15\%$ of an average magnitude) oscillations. The correlated space $\int{\delta V _{R}dt}$ and ground $J_{up}$ observations have revealed that the ionospheric current dynamics lags behind THEMIS observations by about 45 s. This time delay is about $15\%$; the observed oscillation period of $\int{\delta V_{R}dt}$ is about 5 minutes. This represents a phase lag of about 1 radian, which is consistent with Fig. 25 of Wolf et al. (2012) for a reasonable level of an average Pedersen conductance in the ionosphere of 3 S.

The intervals of positive $\int{\delta V_{R}dt}$ correspond to about $10\mbox{--}15\%$ increases in the ionospheric field-aligned currents (Fig. 37d). Every peak in the field-aligned currents corresponds to enhanced auroral luminosity (Fig. 37e) and velocity (Fig. 37f) of the auroral arcs like the one shown in Fig. 37i around RANK at 6:17:30 UT. The arcs were longitudinally oriented and moved equatorward at a velocity up to 200 km/min (with an average value of the order of 50 km/min, Fig. 37f). The velocity of the auroral activity (Fig. 37f) peaked when the magnetic flux tube moved earthward from its equilibrium position. Hence, magnetic flux tube oscillations during fast flow braking in the near-Earth plasma sheet modulated the ionospheric current and auroral dynamics during the substorm under study.

With the help of the thin filament approach (Wolf et al. 2012; Panov et al. 2015), the oscillatory flow braking between 6:00 UT and 6:40 UT on 23 March 2009 was suggested to occur in an asymmetric potential in which the thin filament oscillations appeared to be anharmonic. The force per unit magnetic flux $F_{x}$ acting on the thin filament in its most earthward position appeared to be about three times larger than $F_{x}$ in the filament’s most tailward position. Thus, the aurora brightened (field-aligned current enhanced) when the thin filament was earthward of its equilibrium position, and dimmed (field-aligned current depleted) when the thin filament was tailward of its equilibrium position.

The total energy consumption in the westward electrojet $\dot{W}_{tot}$ was estimated to be between $0.8\times10^{10}~\mbox{W}$ and $3.5\times10^{10}~\mbox{W}$). Only a tenth of the total Joule heating (about $10^{9}~\mbox{W}$) can be associated with the oscillating plasma sheet fast flows: in Fig. 37d, the amplitudes of the alternating ionospheric currents are about 10% of the DC currents.

*Key points:*

Dipolarizing flux tubes brake in the near-Earth region, overshoot their equilibrium position and oscillate around it. Their oscillations modulate particle precipitation into the ionosphere. At the same time, collisional dissipation in ionospheric plasmas provides rapid damping of those oscillations.

*Open questions:*

What is the role of the buoyancy waves for the inner magnetosphere?

How do the buoyancy waves interact with other fundamental ULF waves?

What are the relative roles of the ionospheric Joule heating and of the azimuthal spreading of buoyancy waves in substorm energy sinking?

## Similar Explosive Activity in Solar Corona and Other Physical Systems

### Solar Flares and Coronal Mass Ejections

The magnetized plasma of the solar corona is characterized by intermittent, explosive events which share some similarity to the explosive activity in the magnetotail. In the corona such activity also occurs over a wide range of scales, as evidenced by Coronal Mass Ejections (CMEs), solar flares, and perhaps the coronal heating process itself. That coronal heating may be intrinsically bursty was first proposed by Parker (1972) and is evidenced by event statistics that display power law behavior in terms of total energy, duration and peak luminosity.

Though the terms describing the phenomenology of magnetospheric and coronal activity are different, there is reason to believe that at least some of the basic physical mechanisms behind energy release are the same: indeed though the plasma density, magnetic field strength, and plasma beta are all different by orders of magnitude, the Alfvén speeds and length scales are remarkably similar in the corona and magnetosphere (Reeves et al. 2008). The source of the energy for coronal activity is in photospheric convection, stored and released in the corona by current carrying magnetic fields: how and where the total energy is stored in quasi-equilibrium configurations, the mechanism of destabilization, and the subsequent routes or channels to dissipation remain fundamental open questions, with significant analogies and some contrasts to the dynamics of the magnetotail.

Solar flares are enormous bursts of radiation, identified with the rise and fall in peak flux level of measured X-ray fluxes. They are often accompanied by the ejection of a large amount of material from the corona. Large solar flares can be seen in white light, such as the famous 1859 event recorded independently by Carrington (1859) and Hodgson (1859), one of the most powerful ever observed (see Tsurutani et al. 2003; Cliver and Dietrich 2013 for a discussion of estimates of the total energy released). Large flares release up to $10^{25}~\mbox{J}$, over a time span of about 2 to 20 minutes. Even if the power in a flare corresponds to a small fraction of the total energy emitted by the Sun ($10^{-6}$ of solar luminosity), the energy release is fast and X-ray emission and accelerated particles are detectable from the earth.

Coronal mass ejections (CMEs) are believed to occur through the sudden conversion of magnetic energy into bulk kinetic energy as well as heating and particle acceleration (Forbes 2000; Sterling and Moore 2005). Models of these events generally include a twisted flux rope, or sheared magnetic arcade, above a distribution of photospheric magnetic flux. Current carried by the flux rope or sheared arcade is the source of free magnetic energy, and eruption occurs as this energy is released through an upward expansion, and diminishment, of the current.

**Fig. 38:**
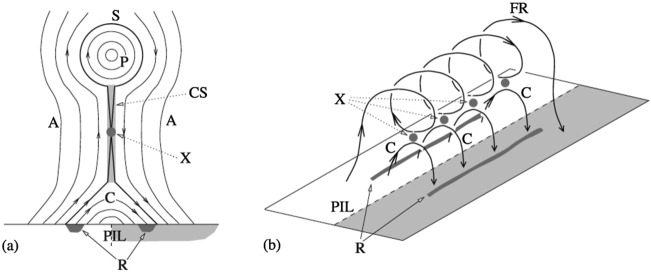
(**a**) Basic elements of the two-ribbon flare model in two dimensions and (**b**) three dimensions (3D). Here, “R” indicates the location of the flare ribbons, “CS” the current sheet, “A” the overlying arcade, “P” the erupting plasmoid, “FR” the 3D flux rope, “PIL” the polarity inversion line, “X” the site(s) of magnetic reconnection, “S” the separatrix boundary of the erupting CME flux rope, and “C” the coronal flare loops formed by magnetic reconnection (from Kazachenko et al. 2017)

The eruption is preceded by a long phase (days to week) during which the magnetic field is progressively stressed and free magnetic energy builds up. The configuration typically evolves quasi-statically (with velocities well below the Alfvén speed). At a certain point in the evolution, within a few minutes up to an hour, the system becomes very dynamic, with a global upward motion, as traced by the evolution of the cold plasma in the associated filament. Later on a flare is typically observed, with a significant release of magnetic energy. Roughly speaking if the downward magnetic tension of the covering magnetic arcade is weak enough, the erupting plasma and magnetic field are launched towards interplanetary space as a CME (for a more detailed discussion on the trigger mechanism for the CMEs see below). Figure 38 illustrates this process using a more recent rendition of the standard (“CSHKP”) model of solar flares (Carmichael 1964; Sturrock 1966; Hirayama 1974; Kopp and Pneuman 1976), which is topologically equivalent to the standard (Near-Earth-Neutral-Line) model of terrestrial substorms (e.g., Baker et al. 1996). Similar to the phases for substorms and perhaps other magnetospheric explosive events, the CME/flare phenomenon occurs in four main phases: buildup, instability, acceleration, and propagation.

CME associated flares show the presence of flare loops and twisted magnetic field lines, identified as a flux rope, formed before or during the eruption. Flare ribbons are the locations in the chromosphere/photosphere with the largest radiative emission increase from optical to soft X-rays. They are a consequence of the impact at the chromosphere of energetic particles launched from the coronal reconnection site. During such flares, a two-ribbon structure is often observed, across the magnetic polarities as illustrated in Fig. 38. The presence of two ribbons with a typical J-shape in the different magnetic polarities argues for the permanence of some shear (corresponding to a magnetotail $B_{y}$ component) in the post-flare phase. The flare ribbons are in several aspects analogous to the two auroral zones, one in each hemisphere on Earth (Akasofu 1979). In the aurora and on the Sun the cooler upflowing plasma is an important component of the flux tube population, though in both the terrestrial and solar cases this filling process takes much longer than the field line shrinkage associated with reconnection (Lin 2004).

Why the magnetic configuration erupts is still an open question. The coronal magnetic configuration becomes unstable at some point during a slow evolution, inviting an interpretation in terms of either transition to a loss of equilibrium or development of an instability. Magnetic reconnection is involved as a key mechanism for the progressive transformation of the magnetic configuration.

There are several initiation models for CMEs. Some initiation models rely on loss of equilibrium or the ideal instability of a flux rope straddling the filament channel neutral line. Slow evolution, driven either by current increase or external flux erosion, brings the flux rope to a point of either non-equilibrium or catastrophe (Heyvaerts and Kuperus 1978; Forbes and Isenberg 1991; Kliem and Török 2006; Fan and Gibson 2007; Démoulin and Aulanier 2010; Olmedo and Zhang 2010; Hassanin and Kliem 2016). Indeed, the upward acceleration phase starts before the impulsive phase of the flare in the majority of the events. The major question in this flux rope picture is that the large amount of shear, which is always present in the preflare filament channel, is an accessory rather than a requirement of this model: while field aligned structures are seen throughout all layers of the visible atmosphere, large-scale twisted structures are not observed except during eruptive solar events. Other models, which do not have a pre-formed flux rope but only an intensely sheared coronal configuration with field lines elongated along the neutral line, require reconnection to initiate the eruption and flaring of the magnetic configuration. In subsequent phases, magnetic reconnection plays a key role in all models for eruption as the peak of the upward acceleration is typically found to be correlated with the peak of the hard X-rays and of the time derivative of soft X-rays flux.

The sheared arcade may be destabilized by reconnection with the overlying field if its topology is not that of a simple bipolar arcade but contains multipolar structure harboring spine-fan structures. This is the essence of the breakout model (Antiochos et al. 1999). Shearing of an arcade within a multiple polarity structure leads to the rise and possible breakout of the structure via reconnection somewhat akin to what occurs in the dayside magnetosphere in the presence of a southward IMF.

Flux-rope models may be applicable to new, rapidly growing active regions (ARs) though it is probably impossible to correctly model a CME by the interactions between a new AR and just any nearby coronal loop system. This would produce a rearrangement of the magnetic fields at the Sun but probably not a CME. Dynamic AR fields serve as a catalyst to successive coronal magnetic field changes and, in circumstance where a filament channel exists, a CME might follow. However, there are many emerging flux regions that are not a catalyst to the occurrence of a CME.

**Fig. 39:**
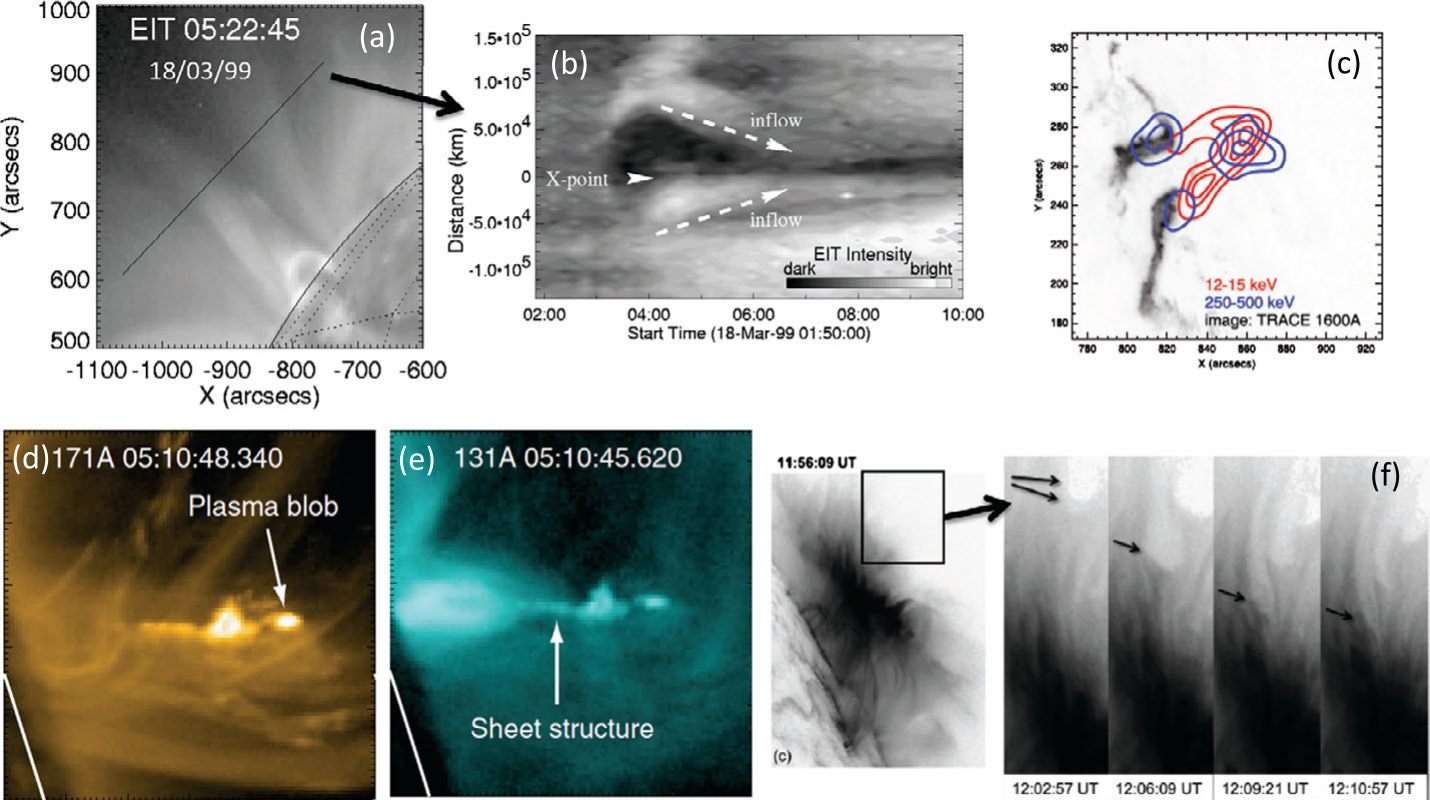
Examples of reconnection signatures in the solar corona. (**a**, **b**) Converging motions (inflows) toward a thin sheet-like region, above flare loops during the 18 March 1999 (SOL1999-03-18T04:04) event as observed by EIT/SOHO (Yokoyama et al. 2001). Panel (**b**) is the time evolution of the emission in 195 Å along the slit shown in (**a**). (**c**) HXR emissions from RHESSI during the 20 January 2005 event (SOL2005-01-20T06:30, (Krucker et al. 2008)), showing the presence of a strong emission source ($>250\text{ keV}$, blue) above the top of the loop (red contours). (**d**, **e**) Observation of a plasmoid ejection during the 18 August 2010 flare (SOL2010-08-18T05:48) and its associated reconnection region, seen within two filters of AIA/SDO instrument (Takasao et al. 2012). (**f**) Observation of a supra-arcade downflow seen as a void propagating sunward in the region above the flare loops, during the 22 October 2011 event (SOL2011-10-22T10:00) in the AIA 131 Å filter (Savage et al. 2012). Adapted from Janvier et al. (2015)

Another model, proposed by Chen and Shibata (2000), suggests that many CMEs are preceded by emerging flux with a polarity orientation favorable for magnetic reconnection between the emerging flux and the pre-existing coronal field. Considering the complexity of the solar atmosphere, the unceasing convective motions and magnetic flux emergence, several triggering factors may take effect collaboratively. No matter how the magnetic structure either reaches a non-equilibrium state or becomes unstable, the magnetic energy release must occur via reconnection (Chen 2011). Observational signatures for reconnection and current sheets in flares are summarized in Fig. 39.

Beyond the flare ribbons and the at least partial detachment of the CME, supra-arcade downflows (often referred to as SADs) are often observed to accompany flares. These are low-emission, elongated, finger-like features seen in active region coronae above post-eruption flare arcades. SADs are intertwined with bright upward growing spikes (McKenzie and Savage 2009, 2011) and are thought to result from the interaction between reconnection outflows from the overlying current sheet with the surrounding hot corona. Some models identify them directly as jet intrusions, other interpret them as Rayleigh Taylor instabilities in the exhaust of a post-eruption current sheet. The supra-arcade downflows may be analogous to the magnetospheric bursty bulk flows (the high-speed, transient earthward flows discussed in paragraph 4). Sometimes these events are also associated with plasma-depleted flux tubes (or bubbles, see Sergeev et al. 1996), as are the supra-arcade downflows observed during solar flares. In addition the downflows have measured speeds comparable to those of bursty bulk flows, $100\mbox{--}600~\mbox{km}\,\mbox{s}^{-1}$ (Sheeley et al. 2004) compared to the speeds $>400~\text{km}\,\mbox{s}^{-1}$ (Baumjohann et al. 1990; Angelopoulos et al. 1994) of bursty bulk flow events. Similar to magnetotail transients, SADs may contribute locally to the heating of plasma (Reeves et al. 2017).

The original stationary magnetic reconnection model of Sweet and Parker (SP) (Parker 1957; Sweet 1958) produced a scaling of the dissipated power with the diffusivity such that, in effect, the time-scales required for dissipation are much too long to be able to explain catastrophic events such as flares. As Parker himself stated in 1963, “The observational and theoretical difficulties with the hypothesis of magnetic-field line annihilation suggest that other alternatives for the flare must be explored”. However, many years later numerical simulations showed that the stationary Sweet-Parker solution might become unstable: Biskamp (1986) showed that the SP current sheet becomes unstable to fast reconnecting modes once a critical value of the Lundquist number $S = LV_{a}/\eta$ (based on the current sheet half-length $L$, with $V_{a}$ the Alfvén speed and $\eta$ the magnetic diffusivity) of $S \sim10^{4}$ is exceeded. A detailed examination of the stability of the SP configuration has led to the definition of the so called super-tearing or plasmoid-chain instability (Loureiro et al. 2007; Bhattacharjee et al. 2009; Huang and Bhattacharjee 2012), where the formation and ejection of a large number of plasmoids is predicted, similar in many ways to the plasmoid-induced reconnection concept and fractal reconnection model previously introduced by Shibata and Tanuma (2001).

However, the plasmoid-chain instability and its predictions led to growth rates which, paradoxically, diverge with increasing Lundquist numbers. This was the basis for the studies carried out in Pucci and Velli (2014), where, via a linear analysis of the resistive tearing instability, it was shown that an aspect ratio scaling with a fractional power of $S$, separates slowly unstable current sheets (i.e. with growth rate scaling as a negative, fractional exponent of the Lundquist number) from those so violently unstable (with a growth rate scaling as a positive exponent of the Lundquist number, including the SP configuration) that they probably should never form in the first place. The critically unstable current sheet has a growth rate, normalized to the Alfvéén time along the sheet L, of order unity and independent of the Lundquist number itself. In particular the critical current sheet is much thicker than a SP sheet, up to 100 times so for $S \simeq10^{12}$, as typical of astrophysical plasmas. Tenerani et al. (2015) extended this work by studying the dynamics of a collapsing current sheet in two dimensions. They showed that for a 2D collapsing sheet, the $x$-points formed by fast, ideal tearing, tend to also collapse, leading to current sheet elongation and reconnection that follows a quasi-self-similar path, with subsequent collapse, elongation, destabilization starting from the X-points formed in the original sheet. As scales become smaller, and the effective Lundquist numbers decrease, the dynamical time-scales decrease, leading to explosive behavior very similar to that suggested by the fractal reconnection scenario (Shibata and Tanuma 2001).

In the solar corona, where inter-species collisions usually provide the dominant dissipation mechanism for reconnection, the Lundquist numbers are $S\simeq10^{12}\mbox{--}10^{14}$. For instance, for $S=10^{13}$ the critical inverse aspect ratio is—for a current sheet half-thickness $a$, corresponding to the Harris sheer $L_{z}$ defined for the CS or TCS in the magnetosphere in previous sections—$(a/L)_{c}\sim S^{-1/3} \simeq5\times10^{-5}$ (Pucci and Velli 2014). For a loop structure of half-length $L\simeq10^{7}\text{ m}$ the critical thickness would be $2a\simeq1000\text{ m}$, with an inner, tearing singular layer half-thickness $\delta$ of $\delta\simeq3\text{ m}$, intermediate between the ion inertial length $d_{i}$, $d_{i}\simeq10~\mbox{m}$, and the ion Larmor radius, $\rho_{i}\simeq10\text{ cm}$, arguing for a study inclusive of the Hall effect. The latter modifies the critical aspect ratio condition as shown in Pucci et al. (2017), Del Sarto et al. (2016).

In the case of the magnetotail, typical conditions in the plasma sheet during a substorm growth phase give $L= 10^{9}\mbox{--}10^{10}~\mbox{cm}$, $n=0.1~\text{cm}^{-3}$, $B=10^{-4}~\mbox{G}$, $T_{\mathrm{e}}=5~\mbox{MK}$, $T_{\mathrm{i}}=50~\mbox{MK}$ (e.g., Kivelson and Russell 1995; Sergeev et al. 1998; Angelopoulos et al. 2013), so the Lundquist number is in the range $S= 10^{15}\mbox{--}10^{17}$, consistent with other estimates (e.g., Ji and Daughton 2011). However, considering the effects of anomalous resistivity, produced by the scattering of lower hybrid drift (LHD) waves with particles (Eastwood et al. 2009), the Lundquist number can go down to $S=10^{10}\mbox{--}10^{11}$. Again, the corresponding tearing mode singular layer thickness now approaches the ion inertial scale $d_{i} \simeq10^{8}~\text{cm}$, implying that reconnection enters the collisionless regime, so that considering following (Del Sarto et al. 2016) the EMHD model, an estimated critical half-thickness would be about $a \simeq5 \times10^{8}~\text{cm}$.

Though there are strong analogies between the onset of a fast tearing-reconnecting instability in the magnetosphere and in the corona, there are also important differences: tail current sheet reconnection is intrinsically collisionless in nature, while collisional effects may be relevant at initiation in the corona (Pucci and Velli 2014). The tail current sheet has a non-vanishing normal component that requires a generalization of the critical aspect ratio calculations for fast tearing: a comprehensive theory connecting fluid to kinetic fast tearing triggering in this case would provide an important step towards clarification (Pucci et al. 2018b). In the corona, similar non-neutral current sheets may be found before the rising prominences transform into CMEs at the onset of eruption, but the guide field in the tail case is small while in the coronal case me be extremely large, and in the latter case photospheric line-tying effects (Velli and Hood 1989; Velli et al. 1990) may also play a relevant role.

The thicknesses discussed for coronal reconnection remain below the resolution of solar telescopes. Yet there is indirect evidence of the type of dynamics discussed above. It is not only in imaging, as illustrated in Fig. 39, but also in the shape of the energy spectra of energetic electron and ions, where the observed power law seem to require some kind of Fermi mechanism readily available in fragmented (plasmoid dominated) current sheets.

*Key points:*

(1) Although the plasma density, magnetic field strength, and plasma beta in the corona and magnetosphere are all different by orders of magnitude, the Alfvén speeds and length scales of the corresponding explosive processes (dipolarizations and plasma ejections) are remarkably similar. (2) Similar to the onset phases for magnetospheric explosive events, the CME phenomenon in the corona occurs in four main phases: buildup, instability, acceleration, and propagation. (3) In the aurora and on the Sun the cooler upflowing plasma is an important component of the flux tube population, though in both the terrestrial and solar cases this filling process takes much longer than the field line shrinkage associated with reconnection. (4) A significant distinction of the onset mechanism of coronal explosions is a substantial shear, associated with the reconnection guide field and relatively low plasma beta. (5) In the solar corona the processes perhaps most similar to magnetotail dipolarizations are the supra-arcade downflows. (6) An important advance in the theory of coronal explosions has been due to the discovery of the plasmoid-chain instability and the so-called “ideal tearing” regimes of magnetic reconnection, when the tearing growth rate is independent of the Lundquist number. Finding similar regimes in practically collisionless plasmas of the magnetotail might be an interesting challenge, especially in the context of the MMS mission observations.

*Open questions:*

How can observations and theories of magnetotail explosions be generalized to explain similar properties in the corona, such as supra-arcade downflows and solar flares?

Does photospheric flux rearrangement in the photosphere provide analogs of the magnetospheric dynamics including closed magnetic flux depletion (CMFD) and/or open magnetic flux accumulation (OMFA)?

What processes might be analogs of the coronal heating in the terrestrial magnetosphere?

What is the role of the $B_{z}$ component (here used in the magnetospheric context and describing the magnetic field normal to the CS plane) in stabilizing the multi-plasmoid instability and are there analogs in the slow filament rise to the formation of non-monotonic $B_{z}$ gradients (with height, rather than tailward)?

### Laboratory Experiments for the Magnetosphere and Corona

While there are a number of dedicated magnetic reconnection experiments (see, e.g. Yamada et al. 2010, 2016, for reviews) we focus here on recent work of the Magnetic Reconnection Experiment (MRX) in Princeton, which allows study of reconnection in current sheets of variable collisionality. In the MRX a well-defined reconnection layer is generated in a controlled manner. The effect of collisions can be reduced to reveal Hall effects provided by different motions of electrons and ions (Ren et al. 2005), and the dynamics of the reconnection layer can be studied extensively, including the features of both the electron diffusion layer and the ion diffusion layer. Even if reconnection in the magnetotail is driven by the solar wind and the Lundquist numbers are much larger than in laboratory plasmas ($S \sim10^{15}$ vs. $S \sim10^{3}$ (Yamada et al. 2006, 2014)), the fundamental processes should be similar, while the laboratory boundary conditions are adjusted to mimic space plasma configurations.

Due to multiple ($\sim100$) sensors, similar to separate space probes (e.g., Dorfman et al. 2014), the MRX provides an opportunity of the comprehensive investigation of the energy conversion in collisionless plasmas, and in particular an inventory of the energy conversion in collisionless magnetic reconnection. Studies of energy partition have been carried out with MRX experiment (Yamada et al. 2014) in comparison with PIC simulations (VPIC) and Cluster data (Eastwood et al. 2010, 2013). The latter analyses focused on the vicinity of the diffusion region, before the outflow jets interact with the dipolar field region, which is the same area that MRX can investigate entirely. The MRX analysis showed that, consistent with Cluster observations in the magnetotail, more than half of the magnetic energy flux is converted to the particle energy flux, which is dominated by the ion enthalpy flux, with smaller contributions from both the electron enthalpy and heat flux. The half length of the tail reconnection layer was estimated to be $2000\mbox{--}4000~\text{km}$, namely $3\mbox{--}6\,d_{i}$, very similar to the MRX case, $L \sim3\, d_{i}$. In analogy with (Birn and Hesse 2005), the energy conversion rate to electrons and ions is independently calculated by integrating $\mathbf{{J}_{s}} \cdot\mathbf{E}$ (where s stands for species). Half of the incoming magnetic energy is converted to particle energy at a relatively large reconnection rate $\sim0.2\, v_{A}\, L\, B^{2}$ compared with the $\sim S^{-1/2}\, v_{A}\,L\,B^{2}=0.03\, v_{A}\,L\,B ^{2}$, predicted by MHD models (in particular for phase diagrams which discuss the fluid regimes for magnetic reconnection see Ji and Daughton 2011; Pucci et al. 2017).

About $1/3$ of the energy goes to electrons ($15\%$ of magnetic energy) and $2/3$ to ions ($25\mbox{--}30\%$ of magnetic energy, consistent with what has been measured in reversed field pinch). In the 2.5D simulation study using the VPIC code, a similar result is obtained. The conversion of magnetic energy in the experiment occurs across a broad region, much larger than considered before. The energy deposition rate on electrons $\mathbf{{J}_{e}}\cdot\mathbf{E}$, is concentrated near the X-point in a wider region than predicted by 2D numerical simulations (Pritchett 2010; Ji et al. 2008) so that a notable rise of electron temperature (up to $50\%$) is measured over an area that is much wider than the electron diffusion region. We want to remark that as shown in Le et al. (2013) the current layer and so the ohmic diffusion region ($\mathbf{J}\cdot\mathbf{E}$ different from zero) shows a strong dependence on the mass ratio even if, as mentioned in Sect. 3.2 the reconnection rate seems to be independent of the mass ratio itself. The electron thermal energy is transferred to the exhaust by parallel heat conduction while the energy deposited on the ions is converted to thermal and flow energy with substantial conduction and convection losses. In the case of asymmetric reconnection instead, the electron energy gain is comparable to the ion energy gain and both ions and electron gains are dominated by the thermal component (Yoo et al. 2017).

Recent measurements from MRX (Fox et al. 2018), in agreement with MMS observations (Eriksson et al. 2016; Wilder et al. 2017), show that higher guide fields lead to a higher contribution of parallel energy transfer $E_{\parallel }\cdot J_{\parallel}$, with respect to perpendicular energy transfer $E_{\perp}\cdot J_{\perp}$, to the total energy transfer $\mathbf{E \cdot J}$. Parallel energy transfer becomes dominant in the MRX experiment already at normalized guide field values $B_{g}/B_{0} = 0.8$, suggesting a transition from perpendicular to parallel dominated energy transfer between $B_{g}/B_{0} = 0$ and $B_{g}/B_{0} = 0.8$. Both driven simulations with open boundary conditions (Pucci et al. 2018a) as well as periodic spontaneous reconnection simulations (Li et al. 2018) show a qualitative agreement with this result, confirming the $E_{\parallel }\cdot J_{\parallel}$ transfer to be a characteristic feature of guide field reconnection.

**Fig. 40:**
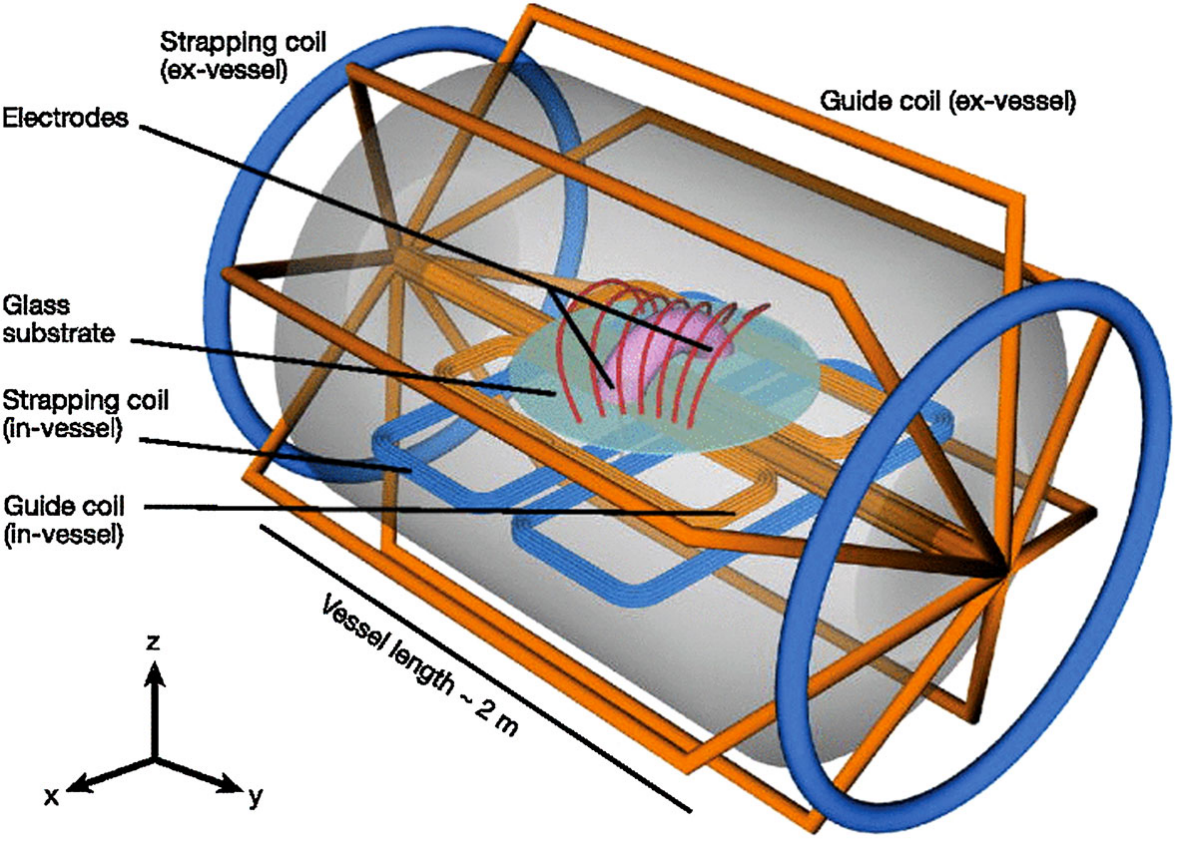
A plasma arc (pink) is maintained between two electrodes that are mounted on a glass substrate. The electrodes, which serve as the flux rope footpoints, are horizontally separated by $2x_{f}=36\text{ cm}$, and they have a minor radius of $a_{f}=7.5\text{ cm}$. The plasma current flows mainly along the arc discharge. Note that the $(x, y, z)$ coordinate system used in these experiments differs from the local reconnection coordinate system used in previous sections. The vertical distance from these footpoints to the vessel wall is $z_{w}=70\text{ cm}$. Four magnetic field coil sets (two inside the vessel, two outside) work in concert to produce a variety of vacuum magnetic field configurations. More specifically, the two orange coil sets are used to produce the guide vacuum field, while the two blue coil sets are used to produce the strapping vacuum field. Reprinted with permission from Myers et al. (2015)

**Fig. 41:**
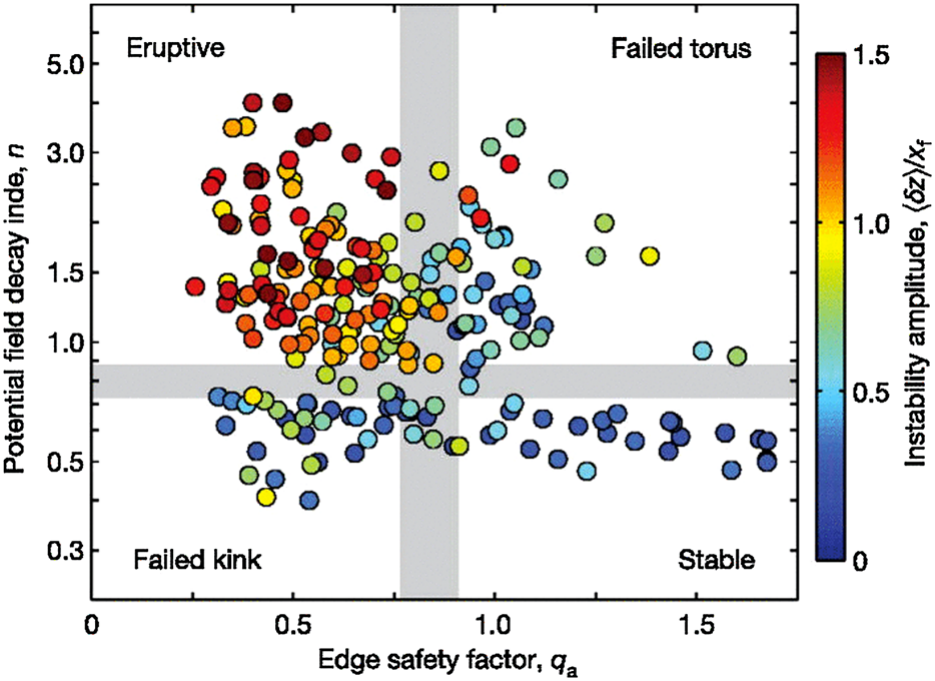
Experimentally measured torus versus kink instability parameter space. The $x$-axis represents the kink instability through the edge safety factor $q_{a}$ (the inverse magnetic twist), while the $y$-axis represents the torus instability through the potential field decay index $n$. Each data point is the mean of 2–5 flux rope plasma discharges with the same experimental parameters. A total of 806 flux rope plasma discharges are represented. The metric used here to quantify the eruptivity of each flux rope is the normalized spatial instability amplitude $\langle\delta z\rangle/x_{f}$. The shaded boundaries, which are empirically identified, delineate the four distinct instability parameter regimes described in the text. Reprinted with permission from Myers et al. (2015)

Modifications of the MRX experiment have also been carried out to study the main physical processes potentially leading to eruptions in an attempt to recreate a solar coronal condition analog, and specifically the instability of a current carrying flux rope (see Myers et al. 2015 and Fig. 40). These specific experiments have shown how both kink and torus instabilities lead to eruption of a line-tied flux tube, but more specifically have pointed to the possible effects required to explain the many failed eruptions which are also seen on the Sun. While the outward forces and current profiles required for the kink and torus instability are well understood, Myers et al. (2015) show that in failed eruption a fundamental attractive force is the tension force associated with the poloidal current and the toroidal field. This force, whose differential effect would be to expand the flux tube itself, when integrated over the cross section and the full toroidal field (made up of both an outer coil induced field and the self-consistent one) is shown to become dominant if the imposed toroidal field is strong enough, as shown in Fig. 41. This is an interesting finding, though the question remains as to whether it is really applicable to the complex geometry of solar prominence configurations.

*Key points:*

(1) Laboratory experiments on magnetic reconnection reached collisionless regimes, typical for Earth’s magnetotail, as is seen from the observed Hall effects. They also provide correlated observations using up to hundred probes, which is similar to recent multi-probe missions. (2) Recent MRX observations of the energy partition in magnetic reconnection match the corresponding analysis using Cluster observations in the magnetotail. Namely, more than half of the magnetic energy flux is converted to the particle energy flux, which is dominated by the ion enthalpy flux, with smaller contributions from both the electron enthalpy and heat flux. (3) Modification of the MRX experiment aimed to model solar coronal conditions reveals the importance of kink and torus instabilities leading to eruption of a line-tied flux tube, which may have similarities with MFRI, B/I and FM instabilities of the magnetotail.

*Open questions:*

What should be the parameters of laboratory experiments, such as MRX and FLARE, to mimic characteristic features of the magnetotail structure? In particular, what is the desired aspect ratio, collisionality, ion to electron temperature ratio, magnetic field line stretching and CS thickness?

How can one reproduce key magnetotail pre-onset processes, OMFA and CMFD, in laboratory experiments?

What experimental setup is necessary to reproduce key magnetotail instabilities (tearing, B/I, flapping, LHD and whistler), transients (DFs, BBFs, ADFs) and near-Earth flux tube oscillations?

What is the laboratory experiment setup to distinguish between EDMR and IDMR reconnection onset scenarios?

Can we distinguish in the laboratory between the Landau damping and nonlinear dissipation effects (e.g. crescent electron orbits near the X-line)?

How may the magnetotail observations, especially the most recent micro-scale MMS studies, help advance the corresponding magnetic reconnection experiments?

## Conclusions

In this paper we attempted to summarize the current understanding of explosive magnetotail processes. The discussion was focused on the recent-decade multi-probe observations, mainly from THEMIS, ARTEMIS, Cluster, POES and MMS missions, as well as the corresponding new findings in theory and simulations. It updates a similar review by Sharma et al. (2008), and complements and expands more recent reviews and book chapters devoted to different aspects of the magnetotail, such as current sheet thinning, magnetic reconnection and particle acceleration (Birn et al. 2012, 2019; Petrukovich et al. 2015, 2016). In particular, an important new aspect of the pre-onset tail current sheet reconfiguration, which is described here in addition to its thinning, is redistribution of the magnetic flux resulting in the formation of regions with the tailward gradient of the equatorial magnetic field $B_{z}$. Their formation is now confirmed by the remote-sensing analysis using electron precipitation features, as well as the empirical reconstruction of the geomagnetic field on substorm scales with data mining techniques. It is also reproduced in global MHD and kinetic RCM simulations. It is explained in theory by the earthward plasma convection from the pre-existing X-line, and perhaps more readily available process of the magnetic flux depletion on near-Earth closed field lines due to the dayside magnetopause reconnection.

Current sheet thinning down to electron scales results in onset of magnetic reconnection with the formation of an EDR, as well as generation of earthward and tailward flows from a new X-line. A new finding is that the reconnection process may also start as the MFRI instability in an ion-scale CS being “internally driven” by fast earthward flows, which are generated spontaneously in the regions with the tailward $B_{z}$ gradient. The latter are also prone to ballooning/interchange and flapping instabilities resulting in a rich 3-D picture of magnetotail transients. The 2-D image of a part of this 3-D picture, corresponding upward FACs, is well resolved in the ionosphere. One of the most promising for future studies and applications beyond the magnetosphere is the discovery of the ideal-MHD regimes of the MFRI, which suggest that similar processes may also occur in the solar wind and corona.

A distinctive feature of transient dipolarizations is the formation and earthward propagation of sharp 1 s-long increases of the $B_{z}$ field coined dipolarization fronts, which is typically more rapid than the associated flow speed increase. The discoveries of their abundance, ion scales and structural stability in their propagation through the tail, as well as the important role of flow channels in populating the inner magnetosphere by energetic plasma have become important findings in the past decade. DFs and following them NFTE/DFB/RFT/FPR structures with elevated $B_{z}$ and reduced plasma density become sources of secondary plasma instabilities, such as the LHDI and mirror instability. They also provide acceleration of plasma particles due to their sharp spatial gradients (e.g., ion reflection from DFs and betatron acceleration of electrons inside DFBs). Braking of dipolarizing flux bundles may have the form of damping oscillations with the main dissipation provided due to their connection with collisional ionospheric plasmas. Similar distributions, and in particular the dawn-dusk asymmetry of earthward and tailward transients suggest that their ultimate source mechanism is the global reconfiguration of the magnetotail via magnetic reconnection.

It is noteworthy that though the mesoscale (BBF-scale) properties of magnetotail dipolarizations are reproduced in many details by global MHD simulations, the non-MHD mechanisms of their formation and near-Earth braking remain topics of continuing debates. As a result, the global picture, including the specific mechanisms of substorms and their contribution to ionospheric dissipations and ring current buildup remains elusive, because it requires kinetic adjustments in the existing global MHD models.

Perhaps the most similar to magnetotail dipolarizations processes in the solar corona are the supra-arcade downflows. At the same time, plasma heating processes in SADs are less obvious and they are much more difficult to investigate. Moreover, while some of the parameters in the coronal dipolarizations, such as their spatial scales and propagation speed are similar to the magnetotail, other parameters, such as the magnetic field and plasma density are larger by several orders. Thus of particular interest for modeling SADs might be MHD models of magnetotail transients. On the other hand, the use of models of explosive processes in the corona, such as for instance, the multi-plasmoid instability, in the magnetotail requires taking into account the stabilizing role of the $B_{z}$ magnetic field, which is now well developed with applications to the terrestrial magnetotail.

Laboratory experiments of collisionless magnetic reconnection are particularly relevant for magnetotail studies because they allow one to investigate the most plausible driver of dipolarizations, magnetic reconnection in a controlled manner and with several dozens of probes, which are still not available in space. This controlled experimental setup allows one to investigate in detail the energy partition of magnetic reconnection as well as roles of various non-reconnection instabilities and turbulent regimes.

## Abbreviations

AACGLat: Altitude-adjusted-corrected geomagnetic latitude
ADF: Anti-dipolarization front
AGSM: Aberrated geocentric solar magnetospheric coordinate system
AR: Active region
ASI: All-sky imager
BBF: Bursty bulk flow
B/I: Ballooning/interchange
CME: Coronal mass ejection
CMFD: Closed magnetic flux depletion
CPCP: Cross polar cap potential
CS: Current sheet
CSHKP: Carmichael-Sturrock-Hirayama-Kopp-Pneuman model
DF: Dipolarization front
DFB: Dipolarizing flux bundle
EDMR: Electron demagnetization-mediated reconnection
EDR: Electron diffusion region
FAC: Field-aligned current
FM: Flapping motion
FPR: Flux pileup region
GEM: Geospace Environment Modeling
GS: Grad-Shafranov
GSM: Geocentric solar magnetospheric coordinate system
IDMR: Ion demagnetization-mediated reconnection
IMF: Interplanetary magnetic field
LC: Loss cone
LFM: Lyon-Fedder-Mobarry
LHDI: Lower-hybrid drift instability
MFRI: Magnetic flux release instability
MHD: Magnetohydrodynamics
MLT: Magnetic local time
MRX: Magnetic Reconnection Experiment
NFTE: Nightside flux transfer event
OFB: Oscillatory flow braking
OMFA: Open magnetic flux accumulation
OpenGGCM: Open Geospace General Circulation Model
PIC: Particle-in-cell
PSBL: Plasma sheet boundary layer
RCM: Rice convection model
RFT: Rapid flux transport region
SAD: Supra-arcade downflow
SAPS: SubAuroral Polarization Streams
SEA: Superposed epoch analysis
TCS: Thin current sheet
WKB: Wentzel-Kramers-Brillouin condition
